# Synthesis and Biological Properties of 5-(1*H*-1,2,3-Triazol-4-yl)isoxazolidines: A New Class of *C*-Nucleosides

**DOI:** 10.3390/molecules20045260

**Published:** 2015-03-24

**Authors:** Salvatore V. Giofrè, Roberto Romeo, Caterina Carnovale, Raffaella Mancuso, Santa Cirmi, Michele Navarra, Adriana Garozzo, Maria A. Chiacchio

**Affiliations:** 1 Dipartimento Scienze del Farmaco e Prodotti per la Salute, Università di Messina, Via S.S. Annunziata, Messina 98168, Italy; E-Mails: ccarnovale@unime.it (C.C.); scirmi@unime.it (S.C.); mnavarra@unime.it (M.N.); 2 Dipartimento di Chimica e Tecnologie Chimiche, Università della Calabria, Via P. Bucci, 12/C, Arcavacata di Rende (CS) 87036, Italy; E-Mail: raffaella.mancuso@unical.it; 3 Dipartimento di Scienze Bio-mediche, Università di Catania, Via Androne 81, Catania 95124, Italy; E-Mail: agar@unict.it; 4 Dipartimento Scienze del Farmaco, Università di Catania, Viale A. Doria 6, Catania 95125, Italy; E-Mail: ma.chiacchio@unict.it

**Keywords:** vinyl triazoles, *C*-Nucleosides, 1,3-dipolar cycloaddition, antiproliferative activity, microwave

## Abstract

A novel series of *C*-nucleosides, featuring the presence of a 1,2,3-triazole ring linked to an isoxazolidine system, has been designed as mimetics of the pyrimidine nucleobases. An antiproliferative effect was observed for compounds **17a** and **17b**: the growth inhibitory effect reaches the 50% in HepG2 and HT-29 cells and increases up to 56% in the SH-SY5Y cell line after 72 h of incubation at a 100 µM concentration.

## 1. Introduction

Structural modification of natural *N*-nucleosides on either the sugar unit or the nucleobase has led to the discovery of a variety of new therapeutic agents, which includes antiviral and anticancer agents [1,2,3,4,5,6,7,8,9]. In this context, the family of *C*-nucleosides constitutes a group of molecules characterized by the link of the sugar unit to a carbon atom of the pyrimidine/purine nucleobase: some of these compounds have been reported to exhibit significant antibacterial, antiviral and antitumoral activities [10].

**Figure 1:**
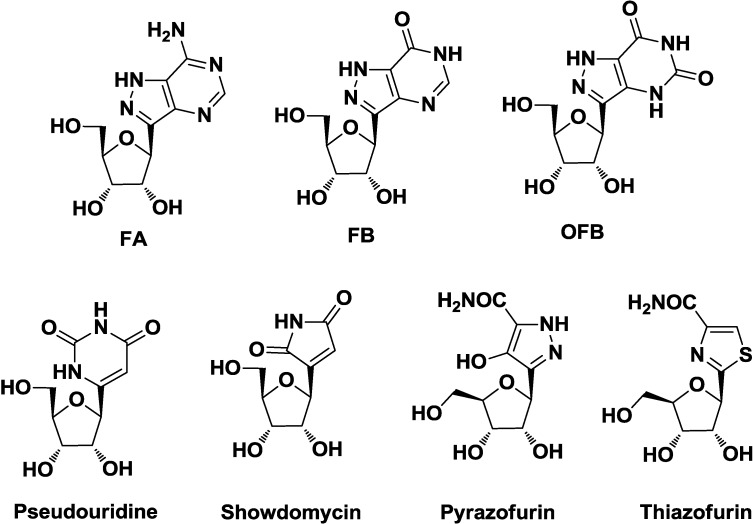
Examples of *C*-Nucleosides.

While natural and synthetic *N*-nucleosides are vulnerable to enzymatic and acid-catalyzed hydrolysis of nucleosidic bond, the *C*-analogues are much more stable. Many natural and synthetic *C*-nucleosides, also containing modified heterocyclic bases, biologically active, have been described in literature. Natural antibiotics formycins [11] (in particular **FA**, **FB**, and oxoformycin B **OFB**) have been known since the early 1960s to possess antibiotic and cytotoxic properties; their antiparasitic activity (antimalarial, antischisostoma, *etc.*) was unraveled later. Pseudouridine is the most abundant natural *C*-nucleoside present in most tRNA and rRNA structures [12], where it has been shown to stabilize RNA duplex [13,14]. Showdomycin [15] possesses well known antibiotic and cytotoxic properties, involving inhibition of nucleoside transport into the cell [16]. Pyrazofurin [17] and tiazofurin [18] have been shown to possess a wide range of medicinal properties, including antibiotic, antiviral, and antitumor activity (Figure 1).

**Figure 2:**
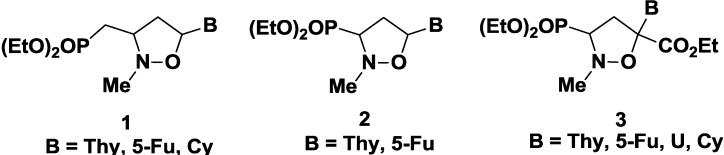
Phosphonated *N,O*-nucleosides.

As part of ongoing efforts to identify new antiviral/antitumor agents, we were interested in the investigation of *N,O*-nucleosides, where the ribose moiety has been replaced by an isoxazolidine system, as mimetic of the sugar unit [19,20,21,22,23]. Phosphonated carbocyclic 2'-oxa-3'-azanucleosides **1** have shown to be potent inhibitors of RT of different retroviruses, following incubation with human PBMCs crude extract [24,25,26]; truncated phosphonated azanucleosides **2** are able to inhibit HIV and HTLV-1 viruses at concentration in the nanomolar range [27]; truncated phosphonated *N,O*-psiconucleosides **3** inhibit HIV *in vitro* infection with low or absent cytotoxicity (Figure 2) [28].

New base-modified *N,O*-nucleosides have been also synthesized. *N,O*-pseudouridine **4** [29] and *N,O-*tiazofurin **5** [30] are characterized by a C-C bond between the isoxazolidine and the heterocyclic systems.

**Figure 3:**
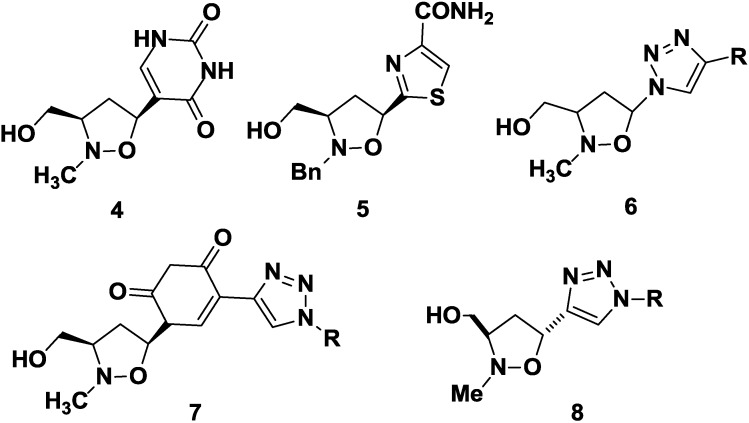
Examples of *N,O*-Nucleosides.

Recently, 1,2,3-triazolyl *N,O*-nucleosides have been designed: 3-hydroxymethyl-5-(1H-1,2,3-triazol)isoxazolidine **6** are able to inhibit proliferation of follicular and anaplastic human thyroid cancer cell lines, with IC_50_ values ranging from 3.87 to 8.76 mM (Figure 3) [31]. In the same context, novel 1,2,3-triazole-appended *N,O*-nucleoside analogs **7** were developed [32]: some of these compounds show a good anticancer activity against the follicular (FTC-133), the anaplastic (8305C) human thyroid cancer cell lines, and especially on the U87MG human primary glioblastoma cell line (Figure 3).

According to these promising results, we have developed a novel series of *C*-nucleosides featured by the presence of a 1,2,3-triazole ring, linked to an isoxazolidine system, as mimetic of the pyrimidine nucleobases. We report in this paper the synthesis of 5-(1H-1,2,3-triazol-4-yl)isoxazolidines **8** and their biological activities as antiviral and/or antitumoral agents.

## 2. Results and Discussion

**Scheme 1:**
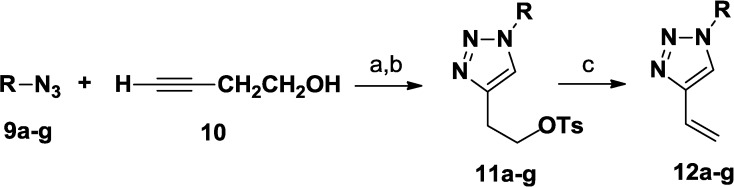
Synthesis of *C*-Vinyl triazoles **12a**–**g**.

*C*-Vinyl triazoles **12a**–**g** have been prepared according to Scheme 1 [33]. Thus, 3-butyn-1-ol **10** was reacted with a variety of azides **9a**–**g**, by click chemistry reactions, performed in H_2_O/*tert*-BuOH (1:1) in the presence of sodium ascorbate, copper(II) sulfate and TEA at room temperature. The obtained triazole derivatives have been tosylated and then converted into vinyl triazoles **12a**–**g** by reaction with potassium *tert*-butoxide in *tert*-butanol (78%–90% yields).

*Reagents and conditions*: (a) CuSO_4_·5H_2_O, sodium ascorbate, TEA, 4 h, rt; (b) tosyl chloride, TEA, CH_2_Cl_2_, rt, 12 h; (c) *t*-BuOK, *t*-BuOH, 40 °C, 12 h.

**Scheme 2:**
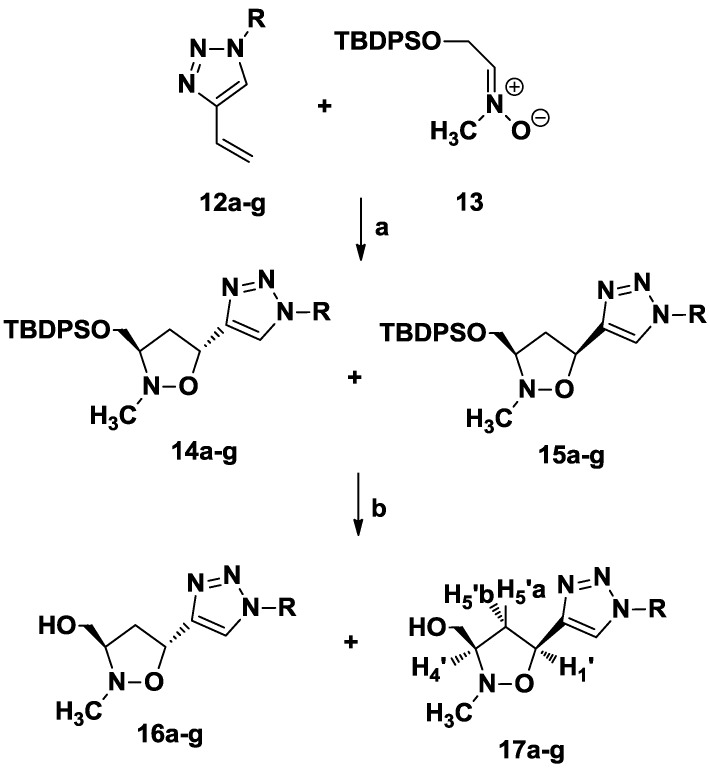
Synthesis of isoxazolidinyl-triazoles **16a**–**g** and **17a**–**g** by 1,3-dipolar cycloaddition.

The 1,3-dipolar cycloaddition of 1-substituted-4-vinyl-1,2,3-triazoles **12a**–**g** with *C-*[(*tert-*butyldiphenylsilyl)oxy]-*N*-methylnitrone **13** [34,35], at 150 W, 80 °C for 2 h in CHCl_3_, proceeded with a good yield and a complete regioselectivity to give a mixture of *trans*/*cis* isoxazolidines **14a**–**g** and **15a**–**g** in a 1:1.3 relative ratio (global yield 80%–85%). Removal of the TBDPS protecting group was accomplished under standard conditions, by treating the diastereomeric mixture with TBAF in THF, to afford the triazolyl nucleosides **16a**–**f** and **17a**–**f**, which were separated by silica gel chromatography (Scheme 2, Table 1).

The diastereomeric ratio of the products was determined by ^1^H NMR spectroscopy of the crude reaction mixture, whereas the relative configuration was assigned by NOEDS spectra. In particular, in the *cis* derivative **17a**, chosen as model compound, a positive NOE effect observed for H-4' and H-5'b (the downfield resonance of protons at C-5', 2.91 ppm) upon irradiation of H-1'(δ = 5.25 ppm), is clearly indicative of their *cis* relationship. Analogously, irradiation of H-4'(δ = 3.25 ppm) in the same compound gives rise to an enhancement in the signals corresponding to H-1' and H-5'b (δ = 5.25 and 2.91 ppm, respectively). On the contrary, no NOE effect was detected between H-4' and H-1' in compound **16a**.

The absence of *cis/trans* diastereoselectivity can be rationalized by assuming that the *E*-endo attack of the dipolarophile on the nitrone, which leads to *cis* adducts, competes efficiently with the *E*-exo attack, the preferred reaction pathway (steric control) leading to *trans* adducts, because of secondary orbital interactions exerted by the triazole ring. This behavior is also in agreement with literature data [36].

*Reagents and conditions*: (a) CHCl_3_, 150 W, 80 °C, 2 h, 85%; (b) TBAF, THF, r.t., 4–5 h, global yield 93%–96% ratio *trans*/*cis* 1:1.3 or 1:1.

**Table 1:** Vinyl triazoles **12a**–**g** and isoxazolidinyl-triazoles **16a**–**g** and **17a**–**g** produced via Scheme 1 and Scheme 2.

R	Vinyl-Triazole	Yield %	Product	Ratio *trans*:*cis*	Yield % ^a^
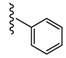	12a	88	16a 17a	1:1.3	92
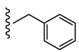	12b	85	16b 17b	1:1.3	94
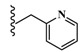	12c	78	16c 17c	1:1.3	93
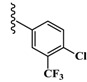	12d	82	16d 17d	1:1	93
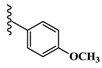	12e	85	16e 17e	1:1.3	93
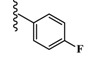	12f	81	16f 17f	1:1.3	95
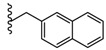	12g	90	16g 17g	1:1	96

^a^ Combined yield.

## 3. Experimental Section

### 3.1. General Information

Solvents and reagents were used as received from commercial sources. Melting points were determined with a Kofler apparatus (Fisher Scientific, Loughborough, UK). Elemental analyses were performed with a Perkin–Elmer elemental analyzer (PerkinElmer, Waltham, MA, USA). NMR spectra (^1^H-NMR recorded at 500 MHz, ^13^C-NMR recorded at 125 MHz) were obtained with a Varian instrument (Agilent Technologies, Palo Alto, CA, USA), and data are reported in ppm relative to tetramethylsilane. Thin-layer chromatographic separations were carried out on Merck silica gel 60-F254 precoated aluminum plates (Merk, Darmstadt, Germany). Flash chromatography was carried out using Merck silica gel (200–400 mesh). Preparative separations were carried out using an Büchi C-601 MPLC instrument (BUCHI Italia S.r.l., Milano, Italy) using Merck silica gel 0.040–0.063 mm, and the eluting solvents were delivered by a pump at the flow rate of 3.5–7.0 mL/min. *C*-[(*tert*-Butyldiphenylsilyl)oxy]-*N*-methyl nitrone was prepared according to described procedures [34,35]. Benzyl/alkyl and aromatic azides were synthesized according to literature procedures [32].

### 3.2. General 1,3-Dipolar Cycloaddition Procedure

A solution of **12a** (0.50 g, 2.92 mmol) and nitrone **13** (1.10 g, 3.50 mmol) in CHCl_3_ (5 mL) was put in a sealed tube and irradiated under microwave conditions at 150 W, 80 °C, for 2 h. The removal of the solvent *in vacuo* afforded a crude material which, after flash chromatography purification by using as eluent a mixture of cyclohexane/ethyl acetate 7:3, gave the unseparable mixture (*trans/cis*) of compound **14a** and **15a**, as yellow oil, that was used for the next reaction, yield 1.24 g (85%). The ^1^H-NMR spectrum of the crude reaction mixture shows the presence of *trans* and *cis* isomers in 1:1.3 ratio, respectively. Compounds **14b**–**g** and **15b**–**g** were prepared by the 1,3-dipolar cycloaddition procedure in 80%–85% yield as yellow oil and then used for the next reaction.

### 3.3. General Desilylation of the Hydroxymethyl Group Procedure: Synthesis of **16** and **17**

A solution of compounds **14a** and **15a** (1.24 g, 2.49 mmol) and TBAF (0.90 mL, 3.73 mmol) in freshly distilled THF (30 mL) was stirred until desilylation was completed (TLC, 4–5 h). Volatiles were flash evaporated, and the residue was purified by MPLC (CH_2_Cl_2_/MeOH, 98:2) to afford **17a**–**g** first eluted isomer (*trans*) and **16a**–**g** second eluted isomer (*cis*) in 92% global yield.

*((3RS,5RS)-2-Methyl-5-(1-phenyl-1H-1,2,3-triazol-4-yl)isoxazolidin-3-yl) methanol* (**16a**): White solid, 52% yield, mp = 57–59 °C. ^1^H-NMR (CDCl_3_): δ = 8.00 (s, 1H), 7.70 (d, *J* = 7.7 Hz, 2H), 7.50 (t, *J* = 7.8 Hz, 2H), 7.42 (t, *J* = 7.4 Hz, 1H), 5.52 (dd, *J* = 8.5, 6.6 Hz, 1H), 3.66–3.52 (m, 2H), 3.31–3.19 (m, 1H), 2.94 (dt, *J* = 12.8, 8.5 Hz, 2H), 2.81 (s, 3H), 2.36 (ddd, *J* = 12.8, 6.6, 4.8, 1H). ^13^C-NMR (CDCl_3_): δ = 148.50, 137.07, 129.86, 128.95, 120.66, 119.84, 71.06, 69.53, 63.42, 44.94, 36.62; Anal. Calcd for C_13_H_16_N_4_O_2_: C, 59.99; H, 6.20; N, 21.52; found C, 60.04; H, 6.27; N, 21.60.

*((3RS,5SR)-2-Methyl-5-(1-phenyl-1H-1,2,3-triazol-4-yl)isoxazolidin-3-yl) methanol* (**17a**): White solid, 40% yield, mp = 100–102 °C. ^1^H-NMR (CDCl_3_): δ = 7.97 (s, 1H), 7.71 (d, *J* = 7.8 Hz, 2H), 7.51 (t, *J* = 7.8 Hz, 2H), 7.43 (t, *J* = 7.4 Hz, 1H), 5.25 (t, *J* = 8.0 Hz, 1H), 3.68 (ddd, *J* = 17.2, 11.4, 4.8 Hz, 2H), 3.25–3.18 (m, 1H), 2.98–2.85 (m, 1H), 2.83 (s, 3H), 2.62 (ddd, *J* = 12.7, 8.0, 5.0 Hz, 1H). ^13^C-NMR (CDCl_3_): δ = 148.15, 137.06, 129.89, 128.99, 120.72, 120.49, 72.39, 69.36, 62.35, 45.70, 36.78; Anal. Calcd for C_13_H_16_N_4_O_2_: C, 59.99; H, 6.20; N, 21.52; found C, 60.08; H, 6.26; N, 21.48.

*((3RS,5RS)-5-(1-Benzyl-1H-1,2,3-triazol-4-yl)-2-methylisoxazolidin-3-yl) methanol* (**16b**): Compound **16b** was prepared by the general desilylation procedure in 53.1% yield as yellow oil. ^1^H-NMR (CDCl_3_): δ = 7.46 (s, 1H), 7.38–7.30 (m, 3H), 7.26–7.23 (m, 2H), 5.51–5.43 (m, 2H), 5.40 (dd, *J* = 8.2, 7.0 Hz, 1H), 3.55–3.46 (m, 2H), 3.23–3.14 (m, 1H), 2.84 (dt, *J* = 12.8, 8.4 Hz, 1H), 2.74 (s, 3H), 2.26 (ddd, *J* = 12.8, 6.7, 4.7, 1H), 2.15 (bs, 1H). ^13^C-NMR (CDCl_3_): δ = 147.87, 134.48, 129.21, 128.88, 128.24, 121.55, 71.00, 69.48, 63.37, 54.32, 44.90, 36.44; Anal. Calcd for C_14_H_18_N_4_O_2_: C, 61.30; H, 6.61; N, 20.42; found C, 61.36; H, 6.67; N, 20.45.

*((3RS,5SR)-5-(1-Benzyl-1H-1,2,3-triazol-4-yl)-2-methylisoxazolidin-3-yl) methanol* (**17b**): Compound **17b** was prepared by the general desilylation procedure in 40.9% yield as white solid, mp = 117–119 °C. ^1^H-NMR (CDCl_3_): δ = 7.43 (s, 1H), 7.39–7.33 (m, 3H), 7.28–7.24 (m, 2H), 5.54–5.44 (m, 2H), 5.12 (t, *J* = 7.9 Hz, 1H), 3.63 (ddd, *J* = 17.2, 11.4, 4.8, 2H), 3.10–3.18 (m, 1H), 2.82 (dt, *J* = 12.7, 8.3, 1H), 2.76 (s, 3H), 2.52 (ddd, *J* = 12.7, 7.9, 5.0, 1H). ^13^C-NMR (CDCl_3_): δ = 147.72, 134.52, 129.24, 128.92, 128.26, 122.10, 72.35, 69.28, 62.31, 54.31, 45.55, 36.77; Anal. Calcd for C_14_H_18_N_4_O_2_: C, 61.30; H, 6.61; N, 20.42; found C, 61.38; H, 6.68; N, 20.47.

*((3RS,5RS)-2-Methyl-5-(1-(pyridin-2-ylmethyl)-1H-1,2,3-triazol-4-yl) isoxazolidin-3-yl)methanol* (**16c**): Compound **16c** was prepared by the general desilylation procedure in 52.6% yield as yellow oil. ^1^H-NMR (CDCl_3_): δ = 7.71 (s, 1H), 7.65 (td, *J* = 7.8, 1.7 Hz, 1H), 7.23 (dd, *J* = 7.0, 5.1 Hz, 1H), 7.16 (d, *J* = 7.8 Hz, 1H), 5.65–5.54 (m, 2H), 5.41 (dd, *J* = 8.5, 6.8, 1H), 3.58–3.48 (m, 2H), 3.23–3.15 (m, 1H), 2.84 (dt, *J* = 12.8, 8.5, 1H), 2.74 (s, 3H), 2.39 (bs, 1H), 2.28 (ddd, *J* = 12.8, 6.5, 4.8 Hz, 1H). ^13^C-NMR (CDCl_3_): δ = 154.31, 149.82, 147.92, 137.49, 123.55, 122.58, 122.37, 70.93, 69.50, 63.32, 55.68, 44.86, 36.42; Anal. Calcd for C_13_H_17_N_5_O_2_: C, 56.71; H, 6.22; N, 25.44; found C, 56.76; H, 6.27; N, 25.48.

*((3RS,5SR)-2-Methyl-5-(1-(pyridin-2-ylmethyl)-1H-1,2,3-triazol-4-yl) isoxazolidin-3-yl)methanol* (**17c**): Compound **17c** was prepared by the general desilylation procedure in 40.4% yield as yellow solid, mp = 76–78 °C. ^1^H-NMR (CDCl_3_): δ = 8.66–8.56 (m, 1H), 7.72 (s, 1H), 7.70 (dt, *J* = 7.7, 1.7 Hz, 1H), 7.33–7.26 (m, 1H), 7.23 (d, *J* = 7.8 Hz, 1H), 5.73–5.59 (m, 2H), 5.18 (t, *J* = 7.8 Hz, 1H), 3.66 (ddd, *J* = 17.2, 11.4, 4.8 Hz, 2H), 3.17 (s, 1H), 2.93–2.81 (m, 1H), 2.80 (s, 3H), 2.62–2.52 (m, 1H). ^13^C-NMR (CDCl_3_): δ = 154.31, 149.82, 137.49, 123.55, 122.58, 122.37, 70.93, 69.50, 63.32, 55.68, 44.86, 36.42; Anal. Calcd for C_13_H_17_N_5_O_2_: C, 56.71; H, 6.22; N, 25.44; found C, 56.78; H, 6.30; N, 25.50.

*((3RS,5RS)-5-(1-(4-Chloro-3-(trifluoromethyl)phenyl)-1H-1,2,3-triazol-4-yl)-2-methyl isoxazolidin-3-yl)methanol* (**16d**): Compound **16d** was prepared by the general desilylation procedure in 46.5% yield as yellow solid, mp = 94–96 °C. ^1^H-NMR (CDCl_3_): δ = 8.07 (s, 1H), 8.06 (d, *J* = 2.6 Hz, 1H), 7.86 (dd, *J* = 8.7, 2.6 Hz, 1H), 7.65 (d, *J* = 8.7 Hz, 1H), 5.49 (dd, *J*=8.7, 6.3 Hz, 1H), 3.58 (d, *J* = 5.7 Hz, 2H), 3.26–3.18 (m, 1H), 2.94 (dt, *J* = 12.8, 8.7, 1H), 2.79 (s, 3H), 2.35 (ddd, *J* = 12.8, 6.3, 4.9, 1H). ^13^C-NMR (CDCl_3_): δ = 149.50, 135.51, 133.11, 132.57, 130.06 (q, *J* = 32.4 Hz), 124.38, 123.24, 121.07, 119.73, 119.64 (q, *J* = 5.5 Hz), 70.94, 69.55, 63.25, 44.92, 36.78, 29.78; Anal. Calcd for C_14_H_14_ClF_3_N_4_O_2_: C, 46.36; H, 3.89; N, 15.45; found C, 46.41; H, 3.93; N, 15.48.

*((3RS,5SR)-5-(1-(4-Chloro-3-(trifluoromethyl)phenyl)-1H-1,2,3-triazol-4-yl)-2-methyl isoxazolidin-3-yl)methanol* (**17d**): Compound **17d** was prepared by the general desilylation procedure in 46.5% yield as yellow thick oil. ^1^H-NMR (CDCl_3_): δ = 8.08 (d, *J* = 2.5 Hz, 1H), 8.00 (s, 1H), 7.89 (dd, *J* = 8.6, 2.5 Hz, 1H), 7.69 (d, *J* = 8.6 Hz, 1H), 5.26 (t, *J* = 7.9, 1H), 3.69 (ddd, *J*=17.1, 11.4, 4.7, 2H), 3.25–3.18 (s, 1H), 2.90 (dt, *J* = 12.8, 8.0, 1H), 2.84 (s, 3H), 2.66 (ddd, *J* = 12.8, 8.0, 5.0, 1H). ^13^C-NMR (CDCl_3_): δ = 149.02, 135.55, 134.94, 133.22, 132.77, 130.11, 127.85, 124.51, 119.77 (q, *J* = 5.3), 72.29, 69.31, 62.28, 45.62, 36.84; Anal. Calcd for C_14_H_14_ClF_3_N_4_O_2_: C, 46.36; H, 3.89; N, 15.45; found C, 46.40; H, 3.95; N, 15.50.

*((3RS,5RS)-5-(1-(4-Methoxyphenyl)-1H-1,2,3-triazol-4-yl)-2-methylisoxazolidin -3-yl)methanol* (**16e**): Compound **16e** was prepared by the general desilylation procedure in 52.6% yield as white solid, mp = 57–59 °C. ^1^H-NMR (CDCl_3_): δ = 7.91 (s, 1H), 7.56 (dd, *J* =8.9, 1.3 Hz, 2H), 6.96 (dd, *J* = 8.9, 1.3 Hz, 2H), 5.48 (dd, *J* = 8.3, 6.8 Hz, 1H), 3.82 (s, H), 3.62–3.53 (m, 2H), 3.25–3.18 (m, 1H), 2.94–2.85 (m, 1H), 2.78 (s, 3H), 2.37–2.29 (m, 1H). ^13^C-NMR (CDCl_3_): δ = 159.89, 148.21, 130.45, 122.21, 120.01, 114.81, 70.99, 69.53, 63.38, 55.67, 44.91, 36.64; Anal. Calcd for C_14_H_18_N_4_O_3_: C, 57.92; H, 6.25; N, 19.30; found C, 57.96; H, 6.28; N, 19.27.

*((3RS,5SR)-5-(1-(4-Methoxyphenyl)-1H-1,2,3-triazol-4-yl)-2-methyl isoxazolidin-3-yl)methanol* (**17e**): Compound **17e** was prepared by the general desilylation procedure in 40.4% yield as white solid, mp = 103–105 °C. ^1^H-NMR (CDCl_3_): δ = 7.88 (s, 1H), 7.60 (d, *J* = 8.9 Hz, 2H), 7.00 (d, *J* = 8.9 Hz, 2H), 5.24 (t, *J* = 7.8 Hz, 1H), 3.85 (s, 3H), 3.67 (ddd, *J*=17.1, 11.4, 4.8 Hz, 2H), 3.22–3.15 (m, 1H), 2.97–2.86 (m, 1H), 2.82 (s, 3H), 2.76 (bs, 1H), 2.60 (ddd, *J* = 12.7, 7.9, 5.0 Hz, 1H). ^13^C-NMR (CDCl_3_): δ = 160.00, 147.89, 130.51, 122.36, 120.66, 114.89, 72.39, 69.35, 55.74, 45.68, 36.79; Anal. Calcd for C_14_H_18_N_4_O_3_: C, 57.92; H, 6.25; N, 19.30; found C, 57.99; H, 6.30; N, 19.33.

*((3RS,5RS)-5-(1-(4-Fluorophenyl)-1H-1,2,3-triazol-4-yl)-2-methylisoxazolidin-3-yl)methanol* (**16f**): Compound **16f** was prepared by the general desilylation procedure in 53.7% yield as white solid, mp = 68–70 °C. ^1^H-NMR (CDCl_3_): δ = 7.96 (s, 1H), 7.71–7.64 (m, 2H), 7.20 (t, *J* = 8.5 Hz, 2H), 5.51 (dd, *J* = 8.5, 6.6 Hz, 1H), 3.58 (d, *J* = 5.7 Hz, 2H), 3.31–3.18 (m, 1H), 2.94 (dt, *J* = 12.8, 8.5 Hz, 1H), 2.80 (s, 3H), 2.36 (ddd, *J* = 12.8, 6.2, 4.9, 1H). ^13^C-NMR (CDCl_3_): δ = 163.56, 161.57, 148.73, 133.37, 122.65 (d, *J* = 8.7 Hz), 120.00, 116.84 (d, *J* = 23.2 Hz), 71.04, 69.51, 63.38, 44.93, 36.65; Anal. Calcd for C_13_H_15_FN_4_O_2_: C, 56.11; H, 5.43; N, 20.13; found C, 56.14; H, 5.49; N, 20.17.

*((3RS,5SR)-5-(1-(4-Fluorophenyl)-1H-1,2,3-triazol-4-yl)-2-methylisoxazolidin-3-yl)methanol* (**17f**): Compound **17f** was prepared by the general desilylation procedure in 41.3% yield as white solid, mp = 102–104 °C. ^1^H-NMR (CDCl_3_): δ = 7.93 (s, *J* = 1.0, 1H), 7.73–7.63 (m, 2H), 7.24–7.14 (m, 2H), 5.24 (t, *J* = 7.8 Hz, 1H), 3.68 (ddd, *J* = 17.2, 11.5, 4.8 Hz, 2H), 3.24–3.17 (m, 1H), 2.97–2.86 (m, 1H), 2.82 (s, 3H), 2.66–2.56 (m, 1H). ^13^C-NMR (CDCl_3_): δ = 163.58, 161.59, 148.26, 133.34, 122.70 (d, *J* = 8.5 Hz), 120.67, 116.86 (d, *J* = 23.2 Hz), 72.30, 69.38, 62.32, 45.68, 36.79; Anal. Calcd for C_13_H_15_FN_4_O_2_: C, 56.11; H, 5.43; N, 20.13; found C, 56.15; H, 5.47; N, 20.19.

*((3RS,5RS)-2-Methyl-5-(1-(naphthalen-2-ylmethyl)-1H-1,2,3-triazol-4-yl) isoxazolidin-3-yl)methanol* (**16g**): Compound **16g** was prepared by the general desilylation procedure in 48.0% yield as white solid, mp = 95–97 °C. ^1^H-NMR (CDCl_3_): δ = 7.83–7.80 (m, 3H), 7.74 (s, 1H), 7.51 (dd, *J* = 6.2, 3.3 Hz, 2H), 7.48 (s, 1H), 7.33 (dd, *J* = 8.4, 1.4 Hz, 1H), 5.70–5.56 (m, 2H), 5.41 (dd, *J* = 8.3, 7.0 Hz, 1H), 3.51 (d, *J* = 6.2 Hz, 2H), 3.21–3.17 (m, 1H), 2.84 (dt, *J* = 12.8, 8.3 Hz, 1H), 2.74 (s, 3H), 2.31–2.22 (m, 1H), 1.91 (bs, 1H). ^13^C-NMR (CDCl_3_): δ = 148.00, 133.32, 131.83, 129.32, 128.07, 127.90, 127.67, 126.87, 125.51, 71.06, 69.44, 63.40, 54.57, 44.89, 36.40; Anal. Calcd for C_18_H_20_N_4_O_2_: C, 66.65; H, 6.21; N, 17.27; found C, 66.68; H, 6.25; N, 17.30.

*((3RS,5RS)-2-Methyl-5-(1-(naphthalen-2-ylmethyl)-1H-1,2,3-triazol-4-yl) isoxazolidin-3-yl)methanol* (**17g**): Compound **17g** was prepared by the general desilylation procedure in 48.0% yield as white solid, mp = 115–117 °C. ^1^H-NMR (CDCl_3_): δ = 7.88–7.79 (m, 3H), 7.74 (s, *J* = 6.8 Hz, 1H), 7.55–7.48 (m, 2H), 7.46 (s, 1H), 7.34 (d, *J* = 8.3 Hz, 1H), 5.72–5.60 (m, 2H), 5.12 (t, *J* = 7.9 Hz, 1H), 3.62 (ddd, *J* = 17.2, 11.5, 4.8, 2H), 3.08–3.16 (m, 1H), 2.90–2.76 (m, 1H), 2.75 (s, 3H), 2.56–2.47 (m, 1H). ^13^C-NMR (CDCl_3_): δ = 147.79, 133.29, 131.85, 129.29, 128.03, 127.89, 127.62, 126.87, 126.84, 125.49, 122.15, 72.35, 69.27, 62.28, 54.50, 45.54, 36.80; Anal. Calcd for C_18_H_20_N_4_O_2_: C, 66.65; H, 6.21; N, 17.27; found C, 66.70; H, 6.27; N, 17.32.

### 3.4. Biological Tests 

#### 3.4.1. Antiviral Activity

Compounds **16**–**17** were evaluated for their ability to inhibit the replication of a variety of DNA and RNA viruses, using the following cell-based assays: (a) Vero cell cultures: poliovirus 1, human echovirus 9, coxsackievirus B4, adenovirus type 2, herpes simplex type 1 (HSV-1), herpes simplex type 2 (HSV-2); (b) human embryonic lung fibroblast cells (MRC-5): cytomegalovirus (CMV: VR-538); (c) African green monkey kidney cells (BS-C-1): varicella-zoster virus (VZV). Acyclovir was used as the reference compounds. Unfortunately, no inhibitory activity against any virus was detected for the evaluated compounds (data not shown).

#### 3.4.2. Antiproliferative Activity

**Figure 4:**
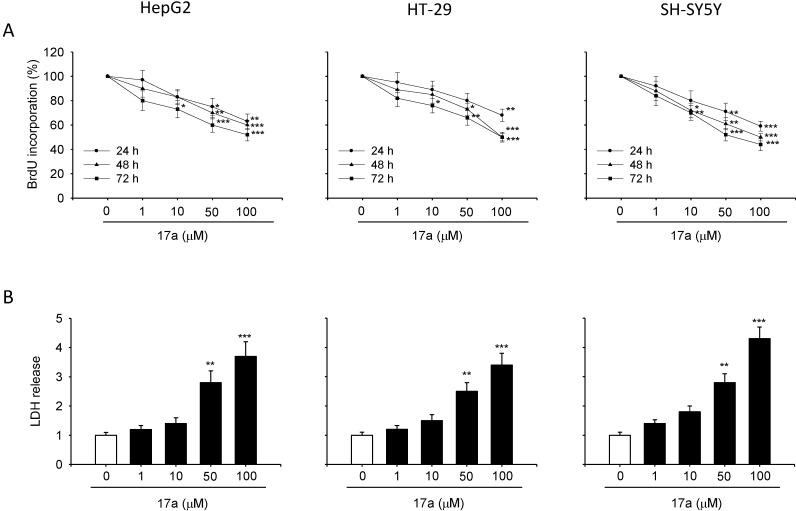
Compound **17a** reduces cell proliferation and induce LDH release. HepG2, HT-29 and SH-SY5Y cells were exposed to increased concentration of **17a** compound for 24, 48 and 72 h. (**A**) Proliferation rate assessed by BrdU assay. (**B**) Cytotoxic effect assessed in terms of LDH release after 24 h of exposure. Each value is the mean ± S.E.M. of three experiments performed eight times (BrdU) or in triplicate (LDH) and repeated three different times. * *p* < 0.05 *vs.* ctrl, ** *p* < 0.01 *vs.* ctrl, *** *p* < 0.001 *vs.* ctrl.

**Figure 5:**
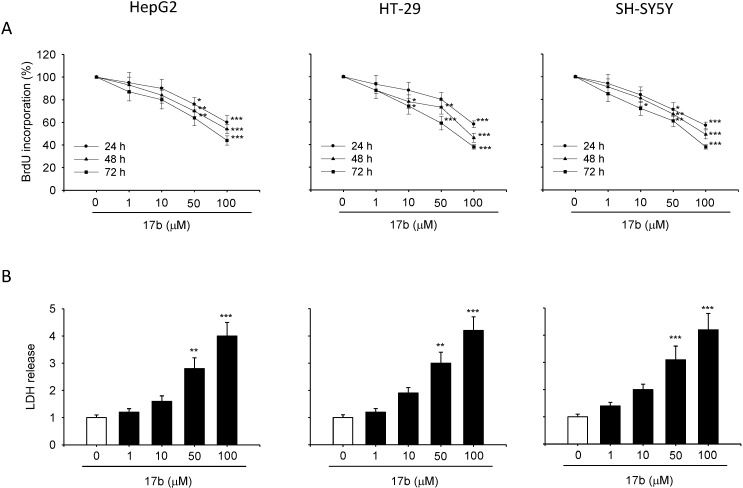
Compound **17b** inhibit cell growth and determine LDH release. (**A**) Proliferation rate assessed by BrdU assay after 24–72 h of treatment. (**B**) Cytotoxic activities evaluated by the test of LDH after 24 h of incubation. Each value is the mean ± S.E.M. of three experiments performed in eight times (BrdU) or in triplicate (LDH) and repeated three different times. * *p* < 0.05 *vs.* ctrl, ** *p* < 0.01 *vs.* ctrl, *** *p* < 0.001 *vs.* ctrl.

The antiproliferative activity of all the synthesized *C*-Nucleosides was evaluated. An antiproliferative effect was observed for compounds **17a** and **17b**, while other derivatives show a IC_50_ in the range 150–200 μM. In particular, as shown in Figure 4A and Figure 5A cell culture incubation with increasing concentration of **17a** and **17b**, ranging from 1 µM to 100 µM for 24, 48 and 72 h, reduced the proliferation in all the cancer cell lines with a similar trend.

In particular, data of the BrdU assay shows that the growth inhibitory effect induced by **17a** reaches the 50% in both HepG2 and HT-29 cells and increases up to 56% in the SH-SY5Y cell line (*p* < 0.001 *vs.* control) after 72 h of incubation at a 100 µM concentration. Reduction of cell proliferation was also observed when the cells were exposed for 48 (*p* < 0.001 *vs.* control) and 24 hours (*p* < 0.01 *vs.* control in HepG2 and HT-29 cells and *p* < 0.001 in SH-SY5Y cell line; Figure 4A) to 100 µM concentration of the compound **17a**.

Lesser antiproliferative effect was observed treating the cells with compound **17a** at concentrations of 50 and 10 µM (expecially for longer time of exposure). Similar results were obtained by the compound **17b** (56% of cell growth inhibition for the HepG2, 62% for the HT-29 and the SH-SY5Y cell lines at the 100 µM concentration for 72 h; Figure 5A). Results of the BrdU assay show very close alignment with those of MTT test performed after 24, 48 and 72 h of incubation with both **17a** and **17b** (see Supplementary Materials Figures S1A and S2A).

#### 3.4.3. Cytoxicity Evaluation

The cytotoxic effect induced by **17a** and **17b** was evaluated through either LDH and trypan blue dye exclusion assays. Figure 4B and Figure 5B show that both **17a** and **17b** caused increase of the LDH release at 50 and 100 µM concentration in all the investigated cell lines. Furthermore, LDH release was accompanied by a significant increase of cell death, as detected by the trypan blue test (see Supplementary Materials Figures S1B and S2B), underlying the cytotoxic activity of the compounds **17a** and **17b**.

#### 3.4.4. Cell Culture and Treatments

HepG2 (hepatocellular carcinoma), HT-29 (colorectal adenocarcinoma) and SH-SY5Y (neuroblastoma) cells were obtained originally from ATCC (Rockville, MD, USA). These cell lines, maintained in RPMI supplemented with 10% heat-inactivated fetal bovine serum, 1 mM sodium pyruvate, 2 mM glutamine, penicillin/streptomycin (100 units/mL and 100 µg/mL, respectively), were grown at 37 °C and 5% CO_2_ conditions. All the reagents for cell cultures were from Gibco (Life Technologies, Monza, Italy). For biological investigations, 100 mM stock solutions were prepared dissolving the tested compounds in dimethyl sulfoxide (DMSO). Small aliquots were stored at −20 °C and were diluted in culture media to the final concentration, ranging from 1 to 100 µM, just prior the use. The highest DMSO concentration used in this study (0.1%) did not have any appreciable effect on cell proliferation or cytotoxicity.

#### 3.4.5. Antiproliferative Activity

The antiproliferative activity of all the synthesized compounds was evaluated by either BrdU and MTT assays. For the first assay, the cells were seeded in 96-well plates at a density of 10 × 10^4^ cell/well (HT-29 cells and SH-SY5Y) and 12 × 10^4^ (HepG2) and allowed to stand overnight. Then, the culture medium was replaced with clean media containing the tested compounds at a concentrations ranging from 1 to 100 µM or with media with equivalent dilutions of DMSO (control cultures). After 24, 48 or 72 h incubation, BrdU assay was performed as described [37]. BrdU is a uridine derivative, structural analog of thymidine, which can be used as a marker for proliferation, because it is incorporated into DNA during the synthesis-phase of the cell cycle as a substitute for thymidine. Cells marked by BrdU incorporation may be detected after addition of goat anti-mouse IgG-peroxidase conjugated secondary antibody. Results are expressed as percentages of the absorbance measured in control cells.

Cell growth was also detected by the MTT assay as reported with modification [38]. The cells were seeded onto 96-well plates at a density of 5 × 10^3^ cells/well (HT-29 cells and SH-SY5Y) or 6 × 10^3^ cells/well (HepG2). The following day, cells were treated with the test compounds as described above. At the end of the exposure time, the plates were centrifuged at 1200 rpm for 10 min, the supernatants were replaced with clean medium without phenol red containing 0.5 mg/mL of 3-(4,5-dimethylthiazole-2-yl)-2,5-diphenyltetrazolium bromide (MTT; Sigma-Aldrich, Milan, Italy), and the plates were returned in the incubator for 4 h. Then, the solutions were removed from the wells and crystals of formazan (MTT metabolic product) were solubilised by 100 µL HCl/isopropanol 0.1 N lysis buffer. The latter were spectrophotometrically quantified at a wavelength of 595 nm (iMark™ microplate reader, Bio-Rad Laboratories, Milan, Italy). Results of the cell proliferation assays are expressed as percentages in untreated cultures. The tests were performed in triplicate (BrdU) or eight times (MTT) and repeated three different times.

#### 3.4.6. Detection of Cell Viability

Lactate dehydrogenase is a stable cytosolic enzyme which is released into the surrounding culture medium when the plasma membrane is damaged, and can be considered a marker of cytotoxicity. The released LDH can be quantified by a coupled enzymatic reaction. First, LDH catalyzes the conversion of lactate to pyruvate via reduction of NAD^+^ to NADH. Second, diaphorase uses NADH to reduce a tetrazolium salt to a red formazan product. Therefore, the level of formazan formation is directly proportional to the amount of released LDH in the medium. HepG2, HT-29 and SH-SY5Y cells were seeded in 96-well plates in a number of 15 × 10^3^ cells/well. The following day, cells were incubated with the synthesized *C*-Nucleosides for 24 h. Then the LDH concentration was measured as reported [39] by using commercial LDH kit (CytoTox 96^®^ Non-Radioactive Cytotoxicity Assay, Promega, Milan, Italy), according to the manufacturer’s protocol. LDH levels are extrapolated as the values detected in control cells, which are arbitrarily expressed as 1.

Cell viability in presence of the tested compounds was assessed also by the trypan blue dye (0.4% w/v) exclusion test [40] and cell death was reported as the percentage of stained (non-viable) *vs.* total cells counted. All the experiments were carried out in triplicate and repeated three times.

#### 3.4.7. Statistical Analysis

Data were expressed as mean ± S.E.M. and statistically evaluated for differences using one-way analysis of variance (ANOVA), followed by Turkey-Kramer multiple comparisons test (GraphPAD Software for Science, GraphPad Software, Inc., La Jolla, CA, USA).

## 4. Conclusions

We report in this paper the synthesis of 5-(1*H*-1,2,3-triazol-4-yl)isoxazolidines, a novel series of *C*-nucleosides, featured by the presence of a 1,2,3-triazole ring linked to an isoxazolidine system, as mimetic of the pyrimidine nucleobases. The synthesized compounds have been evaluated for their ability to inhibit the replication of a variety of DNA and RNA viruses: unfortunately, no inhibitory activity against any virus was detected for the evaluated compounds. The antiproliferative activity of all the synthesized *C*-Nucleosides was also tested. An antiproliferative effect was observed for compounds **17a** and **17b**: the induced growth inhibitory effect reaches the 50% in HepG2 and HT-29 cells and increases up to 56% in the SH-SY5Y cell line (*p* < 0.001 *vs.* control) after 72 h of incubation at a 100 µM concentration.

## Supplementary Materials

Supplementary materials can be accessed at: http://www.mdpi.com/1420-3049/20/04/5260/s1.
