# Supplementary material for: Synthesis and Biological Properties of 5-(1H-1,2,3-Triazol-4-yl)isoxazolidines: A New Class of C-Nucleosides
Source: Molecules. 2015 Mar 24;20(4):5260–75. doi: 10.3390/molecules20045260 (PMC6272400; doi:10.3390/molecules20045260)
Supplement: Supplementary file 1 [file molecules-20-05260-s001.pdf]

Supplementary Materials

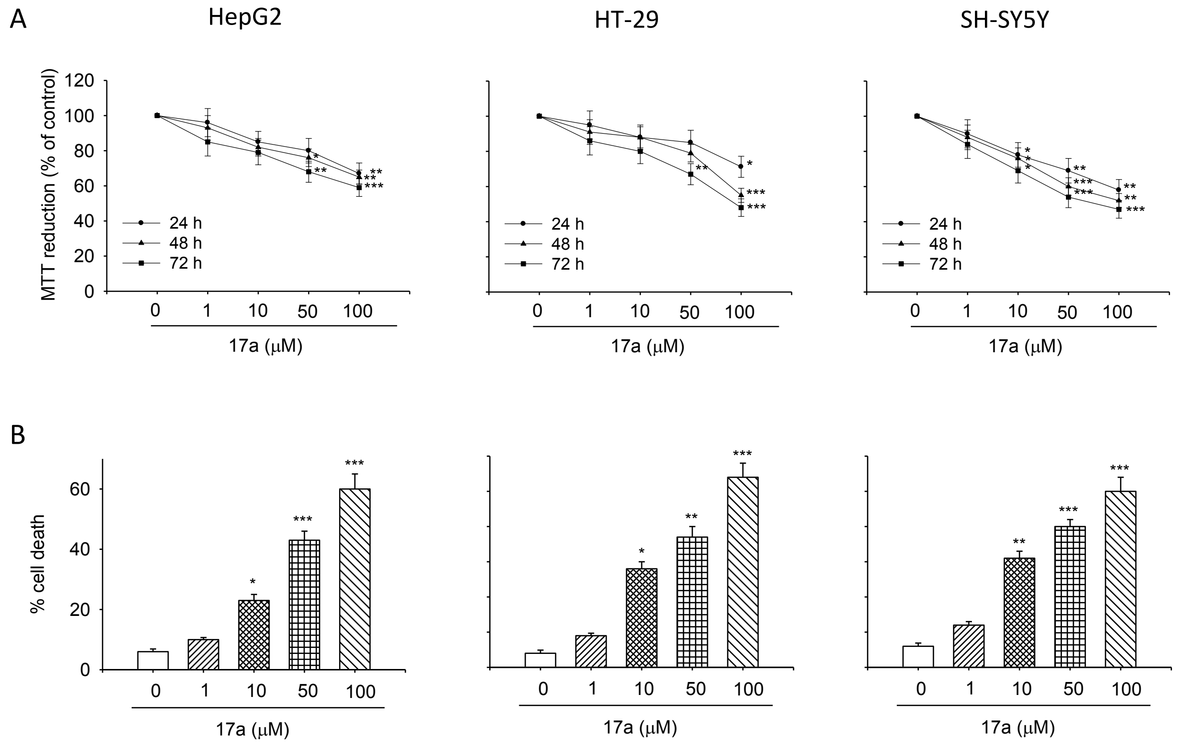

Figure S1. Effect of **17a** compound on cell growth and viability. (**A**) The HepG-2, HT-29 and SH-SY5Y cells were exposed to increased concentration of **17a** compound at the indicated concentrations for 24, 48 and 72 h. Cell proliferation was assessed by the MTT assay. Results are expressed as percentages of growth rates of treated cells compared to control cultures. (**B**) The cells were treated with the **17a** compound at concentrations ranging from 1 to 100 μM for 24 h. Then, cytotoxic effect was evaluated in terms of cell death by the trypan blue test, and was reported as the percentage of stained (non viable) *vs.* total cells counted. Each value is the mean ± S.E.M. of three experiments. * $p < 0.05$ *vs.* ctrl, ** $p < 0.01$ *vs.* ctrl, *** $p < 0.001$ *vs.* ctrl.

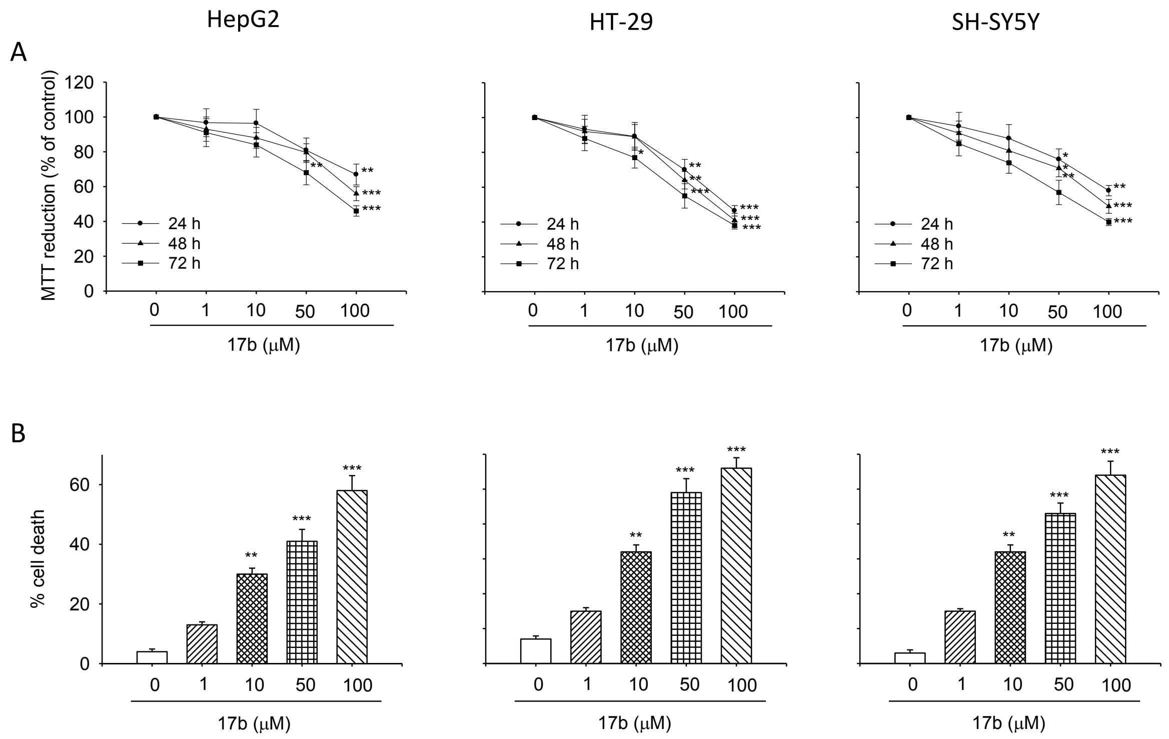

Figure S2. The compound 17b reduces both cell growth and viability. Results of MTT (**A**) and trypan blue (**B**) assays. * $p < 0.05$ *vs.* ctrl, ** $p < 0.01$ *vs.* ctrl, *** $p < 0.001$ *vs.* ctrl.

Chemistry

General Click Chemistry Procedure

To a solution of 3-butyn-1-ol **10** (1.2 equiv.) in *tert*-BuOH (40 mL) and H_2O (40 mL), $CuSO_4{\cdot}5H_2O$ (0.25 equiv.), sodium ascorbate (0.5 equiv.), aryl/alkyl azide **9** (1.1 equiv.) and triethylamine (1.0 equiv.) were added. The mixture was stirred at r.t. under N_2 for 4 h, and then concentrated *in vacuo*. The residue was extracted with CH_2Cl_2 (3 × 30 mL). The organic layer was dried over $MgSO_4$, filtered, and concentrated under reduced pressure. The obtained triazoles were used for the next reaction without further purification.

General Tosylation Procedure

To a solution of **9a** (2.0 g, 10.57 mmol) and TEA (3.24 mL, 23.25 mmol) in DCM (40 mL), tosyl chloride (2.42 g, 12.68 mmol) was added and the resulting mixture was stirred at r.t. for 12 h. Then, the mixture was diluted with water and extracted with ethyl acetate (3 × 30 mL). The organic layer was dried over $MgSO_4$, filtered and concentrated at reduced pressure. The crude product was purified via flash column chromathography (7:3 cyclohexane/ethyl acetate) to yield 3.08 g (85%) of **11a** as white powder, mp = 130–132 °C.

2-(1-Phenyl-1H-1,2,3-triazol-4-yl)ethyl 4-methylbenzensulphonate (**11a**): ^{1}H-NMR (500 MHz, $CDCl_3$): δ = 7.80 (s, 1H), 7.75–7.71 (m, 2H), 7.70–7.66 (m, 2H), 7.55–7.49 (m, 2H), 7.47–7.40 (m, 1H), 7.32–7.27 (m, 2H), 4.36 (t, *J* = 6.4, 2H), 3.17 (t, *J* = 6.4, 2H), 2.39 (s, 3H). ^{13}C-NMR (126 MHz, $CDCl_3$) δ = 145.10, 143.67, 137.09, 132.86, 130.01, 129.87, 128.85, 127.95, 120.52, 120.34, 69.13, 26.04, 21.72. Anal. Calcd for $C_{17}H_{17}N_3O_3S$: C, 59.46; H, 4.99; N, 12.24; found: C, 59.50; H, 5.05; N, 12.31.

2-(1-Benzyl-1H-1,2,3-triazol-4-yl)ethyl 4-methylbenzensulphonate (**11b**): Compound 11b was prepared by the general tosylation procedure in 87% yield as yellow oil. ^{1}H-NMR (500 MHz, $CDCl_3$) δ = 7.72–7.51 (m, 3H), 7.38–7.26 (m, 6H), 7.06 (d, *J* = 7.8 Hz, 1H), 5.45 (s, 2H), 4.24 (t, *J* = 6.6 Hz, 2H), 3.04 (t, *J* = 6.6 Hz, 2H), 2.41 (s, 3H). ^{13}C-NMR (126 MHz, $CDCl_3$) δ = 145.01, 13.61, 134.71, 130.19, 129.96, 129.30, 129.18, 129.05, 128.82, 128.28, 128.09, 127.91, 125.88, 69.02, 54.17, 25.93, 21.33. Anal. Calcd for $C_{18}H_{19}N_3O_3S$: C, 60.49; H, 5.36; N, 11.76; found C, 60.55; H, 5.41; N, 11.81.

2-(1-(pyridin-2-yl-methyl)-1H-1,2,3-triazol-4-yl)ethyl4-methylbenzensulphonate (**11c**): Compound 11c was prepared by the general tosylation procedure in 82% yield as yellow oil. ^{1}H-NMR (500 MHz, $CDCl_3$) δ = 8.57 (d, *J* = 4.3 Hz, 1H), 7.67–7.64 (m, 1H), 7.54 (s, 1H), 7.29 –7.23 (m, 3H), 7.14 (d, *J* = 7.8 Hz, 1H), 7.02 (d, *J* = 7.9 Hz, 2H), 5.59 (s, 2H), 4.26 (t, *J* = 6.6 Hz, 2H), 3.06 (t, *J* = 6.6 Hz, 2H), 2.41 (s, 3H). ^{13}C-NMR (126 MHz, $CDCl_3$) δ = 154.43, 149.77, 145.01, 139.57, 137.54, 129.97, 128.80, 127.94, 125.86, 123.54, 122.83, 69.03, 55.54, 25.96, 21.72. Anal. Calcd for $C_{17}H_{18}N_4O_3S$: C, 56.97; H, 5.06; N, 15.63; found: C, 57.01; H, 5.11; N, 15.70.

2-(1-(4-Chloro-3-(trifluoromethyl)phenyl)-1H-1,2,3-triazol-4-yl)ethyl-4-methylbenzen-sulphonate (**11d**): Compound 11d was prepared by the general tosylation procedure in 88% yield as yellow oil. ^{1}H-NMR (500 MHz, $CDCl_3$) δ = 8.05 (d, *J* = 2.6 Hz, 1H), 7.89 (s, 1H), 7.85 (dd, *J* = 8.7, 2.6 Hz, 1H), 7.74 (d, *J* = 8.3 Hz, 2H), 7.68 (d, *J* = 8.6 Hz, 1H), 7.31 (d, *J* = 8.1 Hz, 2H), 4.36 (t, *J* = 6.2 Hz, 2H), 3.18 (t, *J* = 6.2 Hz, 2H), 2.41 (s, 3H). ^{13}C-NMR (126 MHz, $CDCl_3$) δ = 145.26, 135.55, 133.14, 132.80, 132.51, 130.06, 129.95, 128.68, 127.96, 125.27, 124.29, 119.61 (q, *J* = 5.5 Hz), 68.85, 26.02, 21.73; anal calcd for $C_{18}H_{15}ClF_3N_3O_3S$: C, 48.49; H, 3.39; N, 9.42; found: C, 48.55; H, 3.46; N, 9.49.

2-(1-(4-Methoxyphenyl)-1H-1,2,3-triazol-4-yl)ethyl 4-methylbenzensulphonate (**11e**): Compound 11e was prepared by the general tosylation procedure in 81% yield as yellow solid, mp = 84–86 °C. ^{1}H-NMR (500 MHz, $CDCl_3$) δ = 7.74 (s, 1H), 7.73–7.71 (m, 2H), 7.57 (d, *J* = 8.9 Hz, 2H), 7.32–7.27 (m, 2H), 7.01 (d, *J* = 8.8 Hz, 2H). 4.34 (t, *J* = 6.4 Hz, 2H), 3.86 (s, 3H), 3.15 (t, *J* = 6.4, 2H), 2.40 (s, 3H). ^{13}C-NMR (126 MHz, $CDCl_3$) δ = 159.91, 145.09, 143.41, 132.86, 130.57, 130.01, 127.96, 122.16, 120.51, 114.88, 69.18, 55.76, 26.04, 21.74. Anal. Calcd for $C_{18}H_{19}N_3O_4S$: C, 57.89; H, 5.13; N, 11.25; found: C, 57.93; H, 5.18; N, 11.31.

2-(1-(4-Fluorophenyl)-1H-1,2,3-triazol-4-yl)etil 4-methylbenzensulphonate (**11f**): Compound 11f was prepared by the general tosylation procedure in 80% yield as yellow solid, mp = 92–94 °C. ^{1}H-NMR (500 MHz, $CDCl_3$) δ = 7.78 (s, 1H), 7.74 (d, *J* = 8.2 Hz, 2H), 7.70–7.63 (m, 2H), 7.30 (d, *J* = 8.2 Hz, 2H), 7.21 (t, *J* = 8.5 Hz, 2H), 4.35 (t, *J* = 6.3 Hz, 2H), 3.17 (t, *J* = 6.3 Hz, 2H), 2.40 (s, 3H). ^{13}C-NMR

(126 MHz, $CDCl_3$) δ = 163.49, 161.51, 146.14, 143.83, 132.84, 130.03, 127.96, 122.52 (d, J = 8.6 Hz), 120.56, 116.83 (d, J = 23.3 Hz), 69.06, 26.03, 21.74. Anal. Calcd for: $C_{17}H_{16}FN_3O_3S$: C, 56.50; H, 4.46; N, 11.63; found: C, 56.58; H, 4.52; N, 11.69.

2-(1-(Naphthalen-2-yl-metyl)-1H-1,2,3-triazol-4-yl)ethyl 4-methylbenzensulphonate (**11g**): Compound 11g was prepared by the general tosylation procedure in 89% yield as white solid, mp = 114–116 °C.. ^{1}H-NMR (500 MHz, $CDCl_3$) δ = 7.87–7.80 (m, 3H), 7.74 (s, 1H), 7.67 (d, J = 8.2 Hz, 2H), 7.52 (dd, J = 6.1, 3.3 Hz, 2H), 7.36–7.31 (m, J = 5.3, 2H), 7.21 (d, J = 8.1 Hz, 2H), 5.63 (s, 2H), 4.26 (t, J = 6.6 Hz, 2H), 3.06 (t, J = 6.6 Hz, 2H), 2.38 (s, 3H). ^{13}C-NMR (126 MHz, $CDCl_3$) δ = 145.00, 143.37, 133.34, 133.30, 132.82, 132.10, 129.95, 129.27, 128.09, 127.92, 127.48, 126.89, 126.87, 125.42, 122.14, 69.01, 54.43, 26.03, 21.71. Anal. Calcd for $C_{22}H_{21}N_3O_3S$: C, 64.85; H, 5.19; N, 10.31; found: C, 64.91; H, 5.23; N, 10.38.

General Elimination Procedure

To a solution of 11a (1.0 g, 2.91 mmol) in *tert*-BuOH (20 mL), *tert*-BuOK (0.49 g, 4.36 mmol) was added and the resulting mixture was stirred at 40 °C for 12 h. The mixture was diluted with water and extracted with DCM (3 × 10 mL). The organic layer was dried over $MgSO_4$, filtered and concentrated at reduced pressure to yield 438 mg (88%) of 12a as white solid, mp = 75–77 °C.

1-phenyl-4-vinyl-1H-1,2,3-triazole (**12a**): ^{1}H-NMR (500 MHz, $CDCl_3$) δ = 7.95 (s, 1H), 7.70–7.66 (m, 2H), 7.50–7.44 (m, 2H), 7.41–7.36 (m, 1H), 6.75 (dd, J = 17.7, 11.2 Hz, 1H), 5.97 (dd, J = 17.7, 1.2 Hz, 1H), 5.37 (dd, J = 11.2, 1.2 Hz, 1H). ^{13}C-NMR (126 MHz, $CDCl_3$) δ = 146.90, 136.91, 129.74, 128.72, 125.27, 120.41, 118.39, 116.77.Anal. Calcd for $C_{10}H_9N_3$: C, 70.16; H, 5.30; N, 24.54; found: C, 70.21; H, 5.39; N, 24.59.

1-benzyl-4-vinyl-1H-1,2,3-triazole (**12b**): Compound 12b was prepared by the general elimination procedure in 85% yield as colorless oil. ^{1}H-NMR (500 MHz, $CDCl_3$) δ = 7.41 (s, 1H), 7.37–7.30 (m, 3H), 7.29–7.23 (m, 2H), 6.67 (dd, J = 17.8, 11.2 Hz, 1H), 5.85 (dd, J = 17.8, 1.3 Hz, 1H), 5.50 (s, 2H), 5.31 (dd, J = 11.2, 1.3 Hz, 1H). ^{13}C-NMR (126 MHz, $CDCl_3$) δ = 146.90, 134.74, 129.23, 128.87, 128.15, 125.71, 120.20, 116.24, 54.21. Anal. Calcd for $C_{11}H_{11}N_3$: C, 71.33; H, 5.99; N, 22.69; found: C, 71.39; H, 6.05; N, 22.73.

2-((4-vinyl-1H-1,2,3-triazol-1-yl)methyl)pyridine (**12c**): Compound 12c was prepared by the general elimination procedure in 78% yield as yellow oil. ^{1}H-NMR (500 MHz, $CDCl_3$) δ = 8.58–8.48 (m, 1H), 7.66 (s, 1H), 7.66–7.60 (m, 1H), 7.23–7.19 (m, 1H), 7.14 (dd, J = 7.8, 0.9 Hz, 1H), 6.66 (ddd, J = 17.7, 11.2, 1.2 Hz, 1H), 5.84 (dt, J = 17.8, 1.2 Hz, 1H), 5.58 (s, 2H), 5.28 (dt, J = 11.2, 1.2 Hz, 1H). ^{13}C-NMR (126 MHz, $CDCl_3$) δ = 154.41, 149.74, 146.76, 137.42, 125.56, 123.47, 122.45, 120.93, 116.23, 55.54. Anal. Calcd for $C_{10}H_{10}N_4$: C, 64.50; H, 5.41; N, 30.09; found: C, 64.56; H, 5.46; N, 30.13.

1-(4-Chloro-3-(trifluoromethyl)phenyl)-4-vinyl-1H-1,2,3-triazole (**12d**): Compound 12d was prepared by the general elimination procedure in 82% yield as white solid, mp = 108–110 °C. ^{1}H-NMR (500 MHz, $CDCl_3$) δ = 8.08 (d, J = 2.6 Hz, 1H), 7.97 (s, 1H), 7.91 (dd, J = 8.7, 2.6 Hz, 1H), 7.69 (d,

J = 8.7 Hz, 1H),, 6.78 (dd, *J* = 17.7, 11.2 Hz, 1H), 6.05 (dd, *J* = 17.7, 1.1 Hz, 1H), 5.47 (dd, *J* = 11.2, 1.1 Hz, 1H). ^{13}C-NMR (126 MHz, $CDCl_3$) δ = 147.68, 135.78, 133.17, 129.90, 127.72, 124.89, 124.34, 119.35 (q, *J* = 5.5 Hz), 118.01, 117.87. Anal. Calcd for $C_{11}H_7ClF_3N_3$: C, 48.28; H, 2.58; N, 15.36; found: C, 48.32; H, 2.64; N, 15.39.

1-(4-Methoxyphenyl)-4-vinyl-1H-1,2,3-triazole (**12e**): Compound 12e was prepared by the general elimination procedure in 85% yield as white solid, mp = 77–79 °C. ^{1}H-NMR (500 MHz, $CDCl_3$) δ = 7.85 (s, 1H), 7.61 (d, *J* = 9.1 Hz, 2H), 7.00 (d, *J* = 9.1 Hz, 2H), 6.77 (dd, *J* = 17.7, 11.2 Hz, 1H), 5.97 (dd, *J* = 17.7, 1.2 Hz, 1H), 5.39 (dd, *J* = 11.2, 1.2 Hz, 1H), 3.85 (s, 3H). ^{13}C-NMR (126 MHz, $CDCl_3$) δ = 159.92, 146.88, 130.55, 125.56, 122.24, 118.56, 116.62, 114.88, 55.75. Anal. Calcd for $C_{11}H_{11}N_3O$: C, 65.66; H, 5.51; N, 20.88; found: C, 65.69; H, 5.58; N, 20.92.

1-(4-fluorophenyl)-4-vinyl-1H-1,2,3-triazole (**12f**): Compound 12f was prepared by the general elimination procedure in 81% yield as white solid, mp = 113–115 °C. ^{1}H-NMR (500 MHz, $CDCl_3$) δ = 7.90 (s, 1H), 7.72–7.66 (m, 2H), 7.23–7.18 (m, 2H), 6.76 (ddd, *J* = 17.6, 11.2, 3.1 Hz, 1H), 6.00 (dd, *J* = 17.7, 2.1 Hz, 1H), 5.41 (dd, *J* = 11.2, 3.1 Hz, 1H). ^{13}C-NMR (126 MHz, $CDCl_3$) δ = 161.49, 147.14, 133.32, 129.64, 125.22, 122.53 (d, *J* = 8.6 Hz), 118.54, 117.09, 116.79 (d, *J* = 23.3 Hz). Anal. Calcd for $C_{10}H_8FN_3$: C, 63.49; H, 4.26; N, 22.21; found: C, 63.52; H, 4.29; N, 22.27.

1-(Naphthalen-2-yl-methyl)-4-vinyl-1H-1,2,3-triazole (**12g**): Compound 12g was prepared by the general elimination procedure in 90% yield as white solid, mp = 86–88 °C. ^{1}H-NMR (500 MHz, $CDCl_3$) δ = 8.01–7.94 (m, 3H), 7.71 (s, 1H), 7.50 (dd, *J* = 6.1, 3.2, 2H), 7.44 (s, 1H), 7.32 (d, *J* = 8.3 Hz, 1H), 6.68 (dd, *J* = 17.7, 11.2 Hz, 1H), 5.85 (d, *J* = 17.7 Hz, 1H), 5.62 (s, 2H), 5.30 (d, *J* = 11.2 Hz, 1H). ^{13}C-NMR (126 MHz, $CDCl_3$) δ = 146.85, 133.23, 133.20, 132.04, 129.18, 127.96, 127.82, 127.36, 126.78, 126.76, 125.64, 125.34, 120.32, 116.20, 54.28. Anal. Calcd for $C_{15}H_{13}N_3$: C, 76.57; H, 5.57; N, 17.86; found: C, 76.61; H, 5.63; N, 17.89.

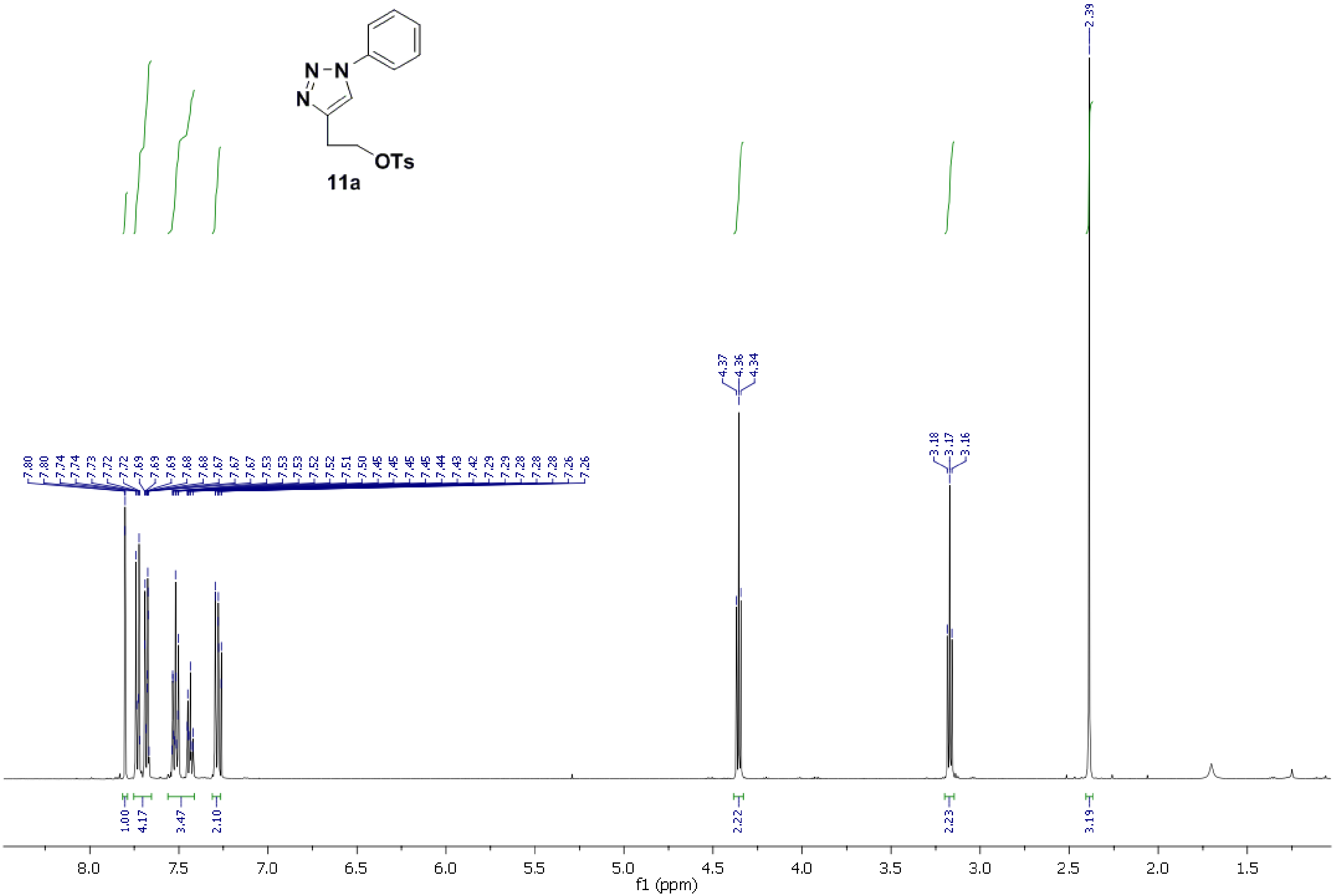

Figure S3. ^{1}H-NMR (500 MHz, $CDCl_3$) of compound **11a**.

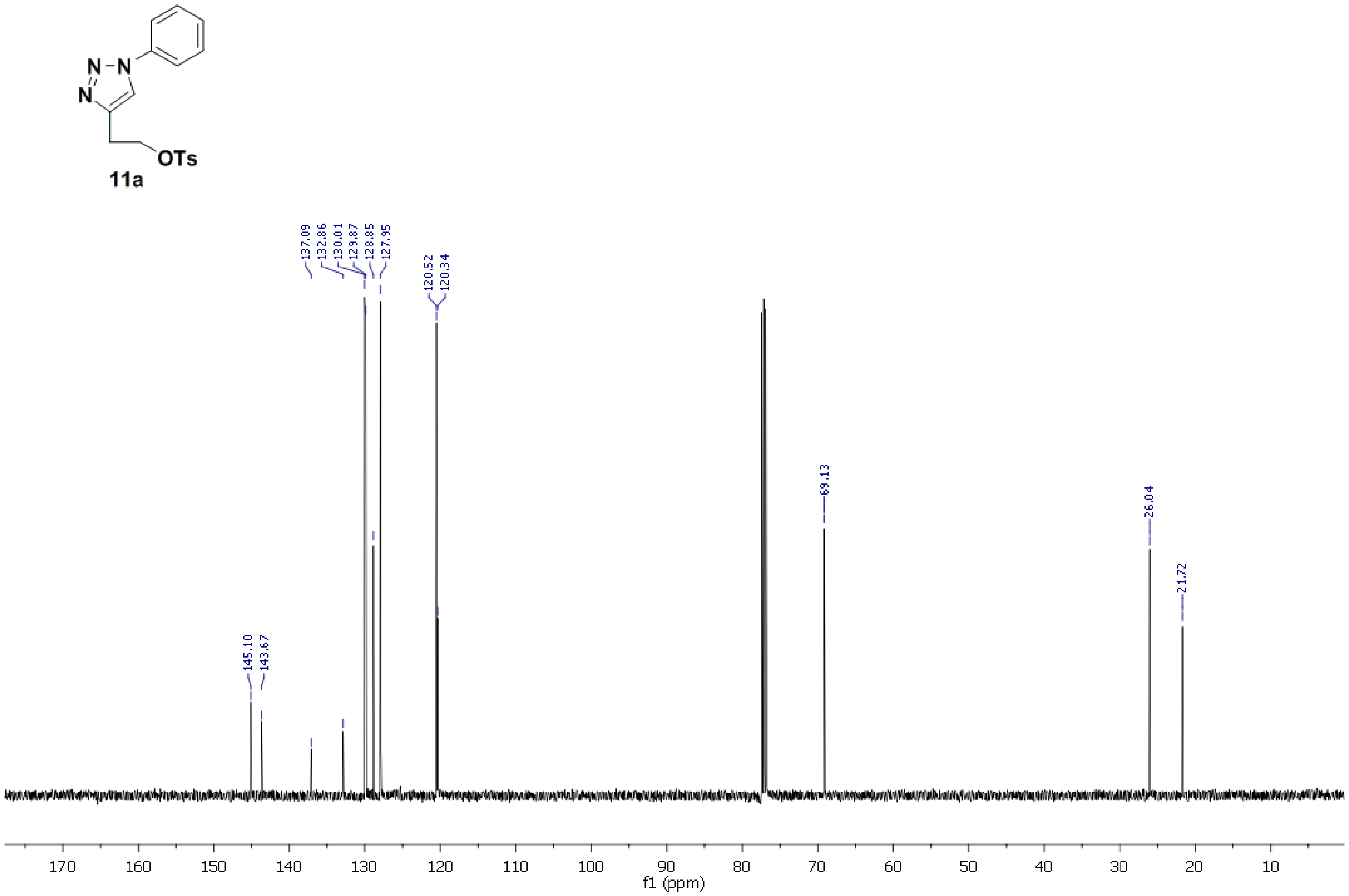

Figure S4. ^{13}C-NMR (125 MHz, $CDCl_3$) of compound **11a**.

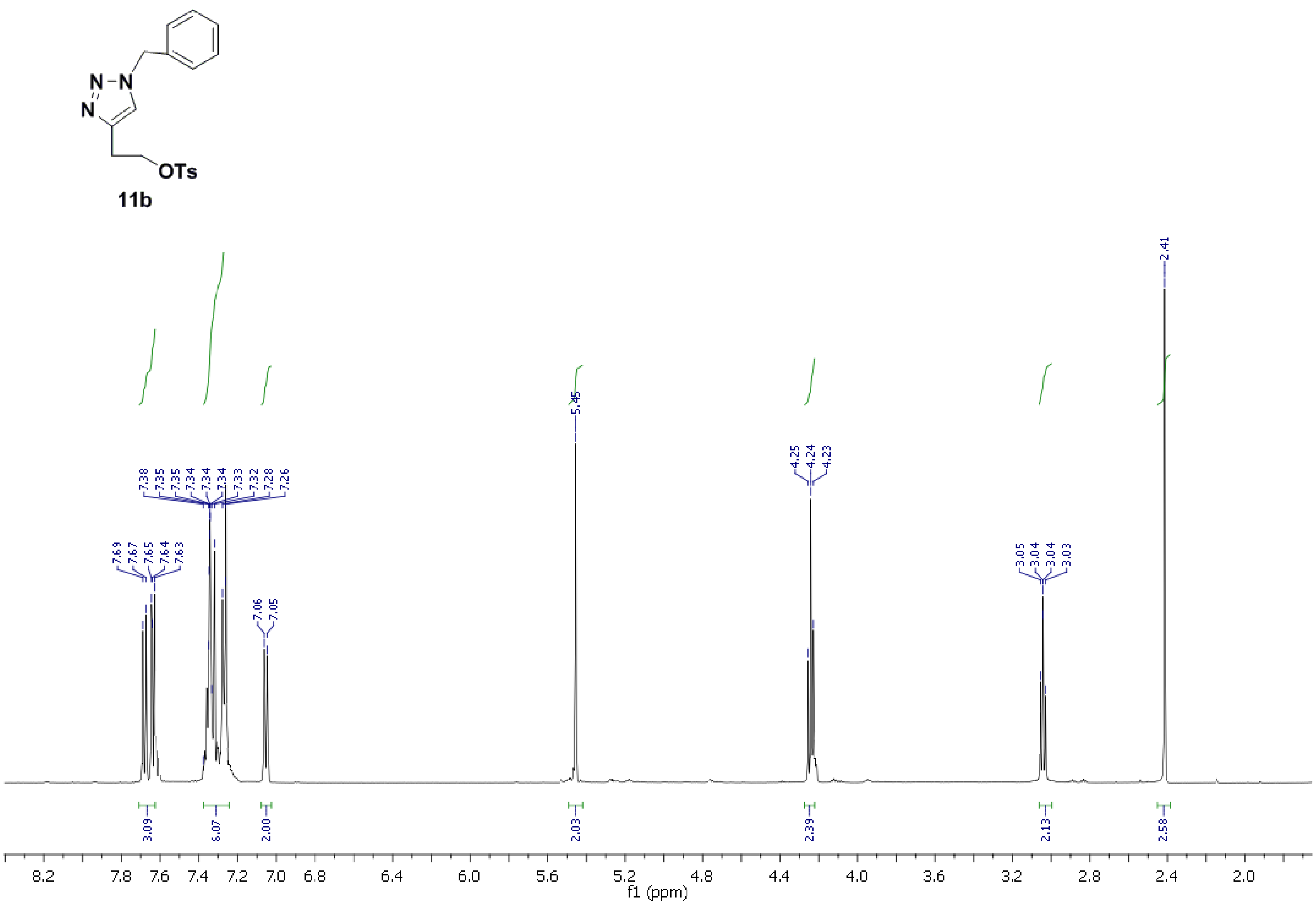

Figure S5. ^{1}H-NMR (500 MHz, $CDCl_3$) of compound **11b**.

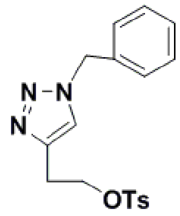

11b

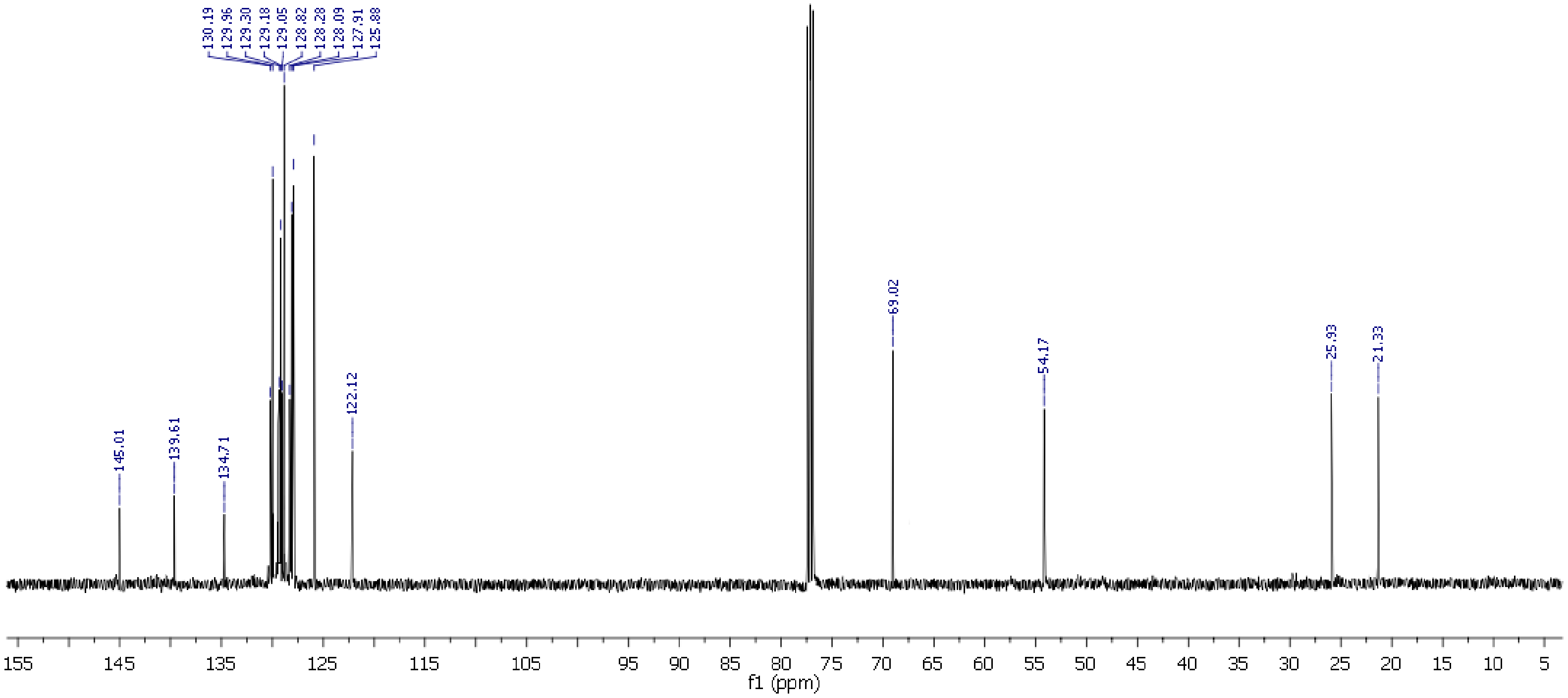

Figure S6. ^{13}C-NMR (125 MHz, $CDCl_3$) of compound **11b**.

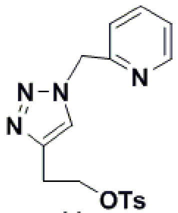

11c

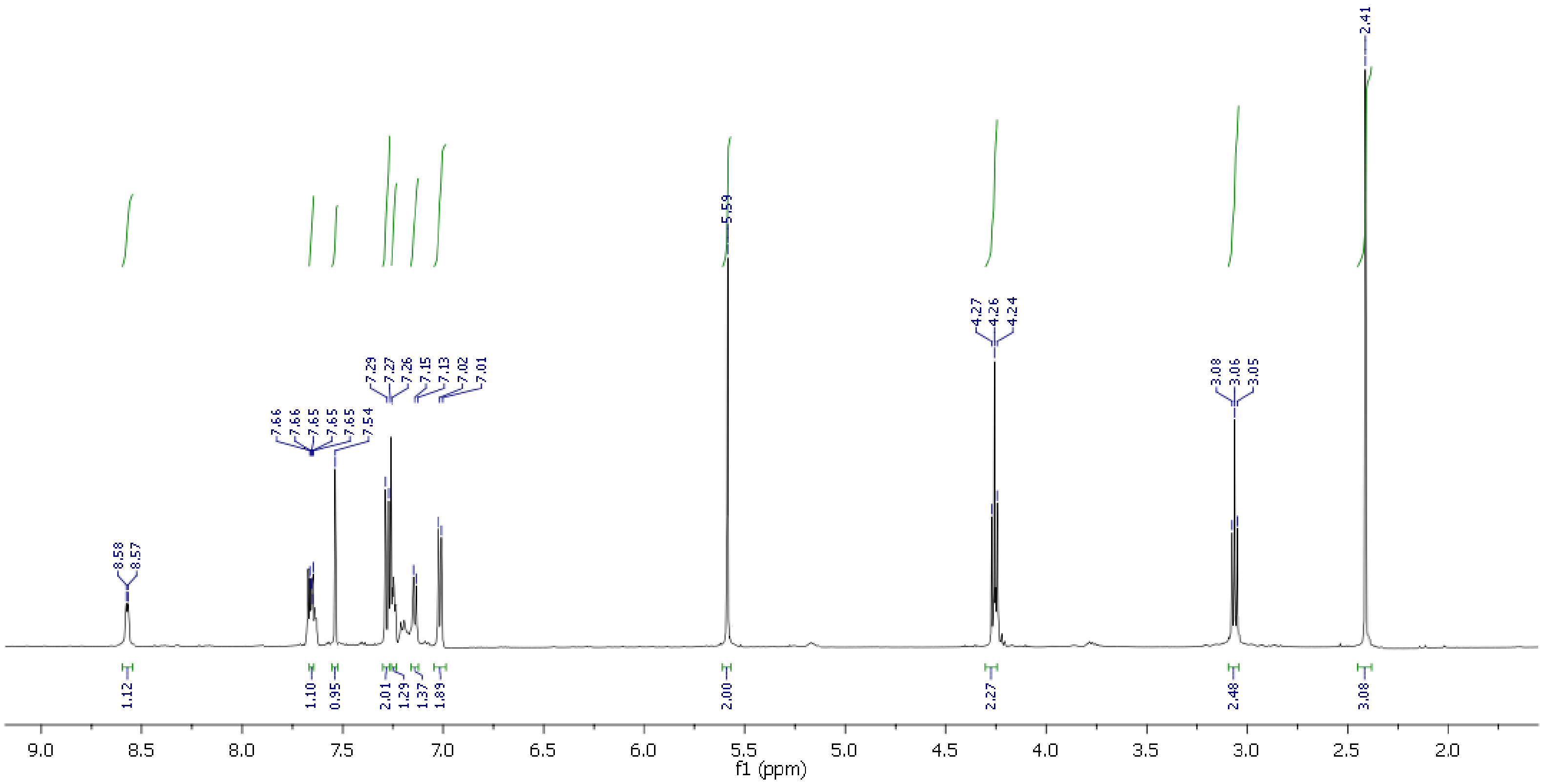

Figure S7. ^{1}H-NMR (500 MHz, $CDCl_3$) of compound **11c**.

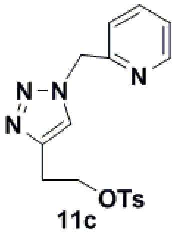

11c

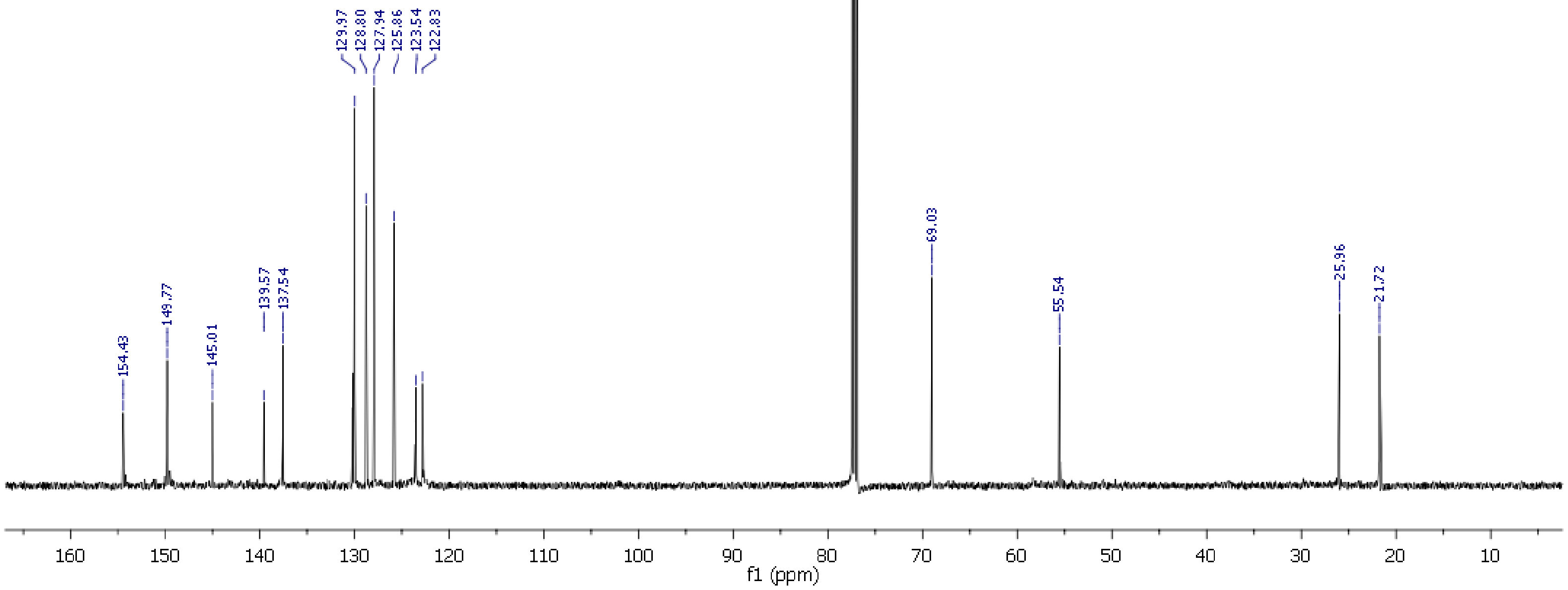

Figure S8. ^{13}C-NMR (125 MHz, $CDCl_3$) of compound **11c**.

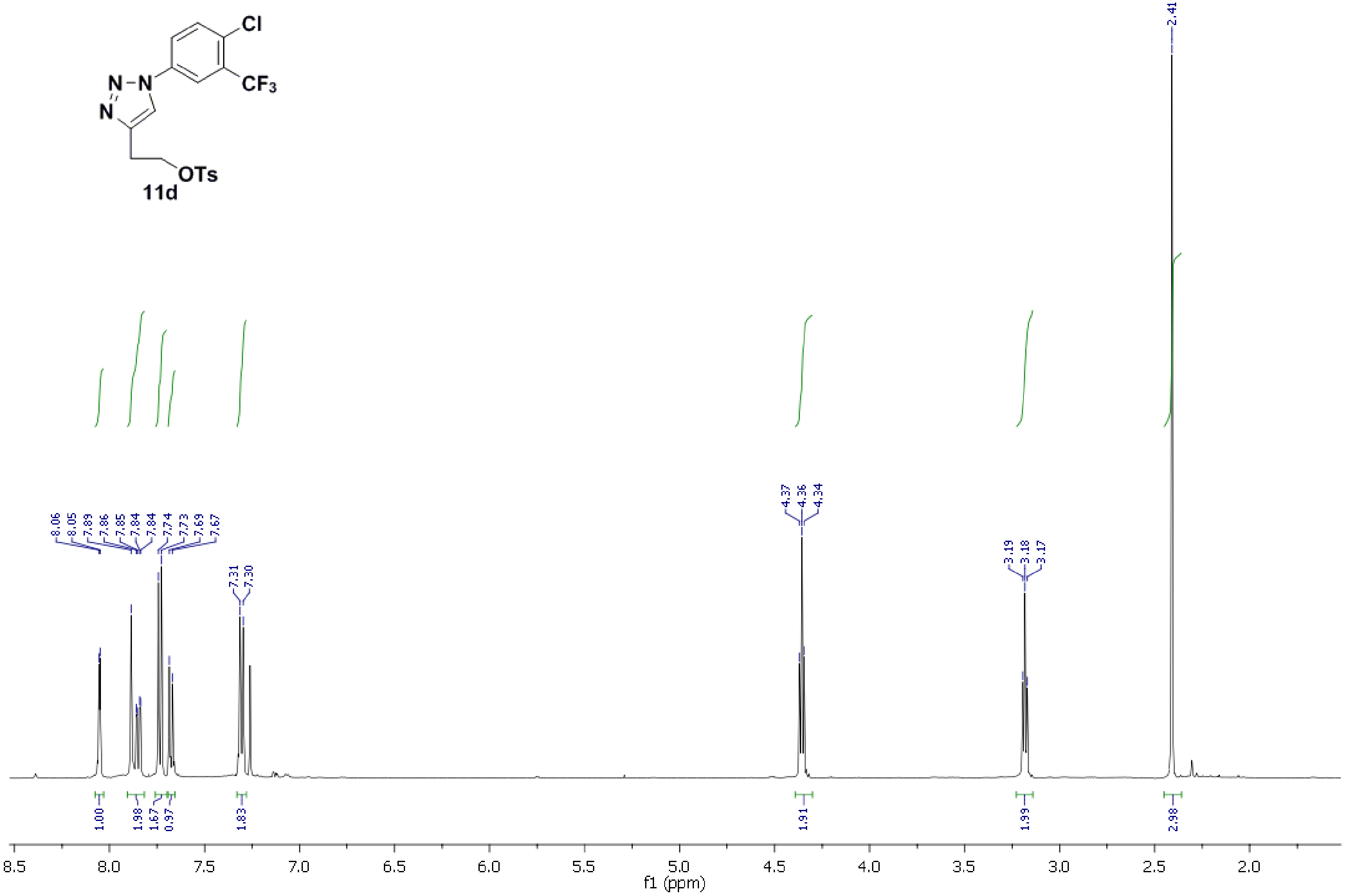

Figure S9. ^{1}H-NMR (500 MHz, $CDCl_3$) of compound **11d**.

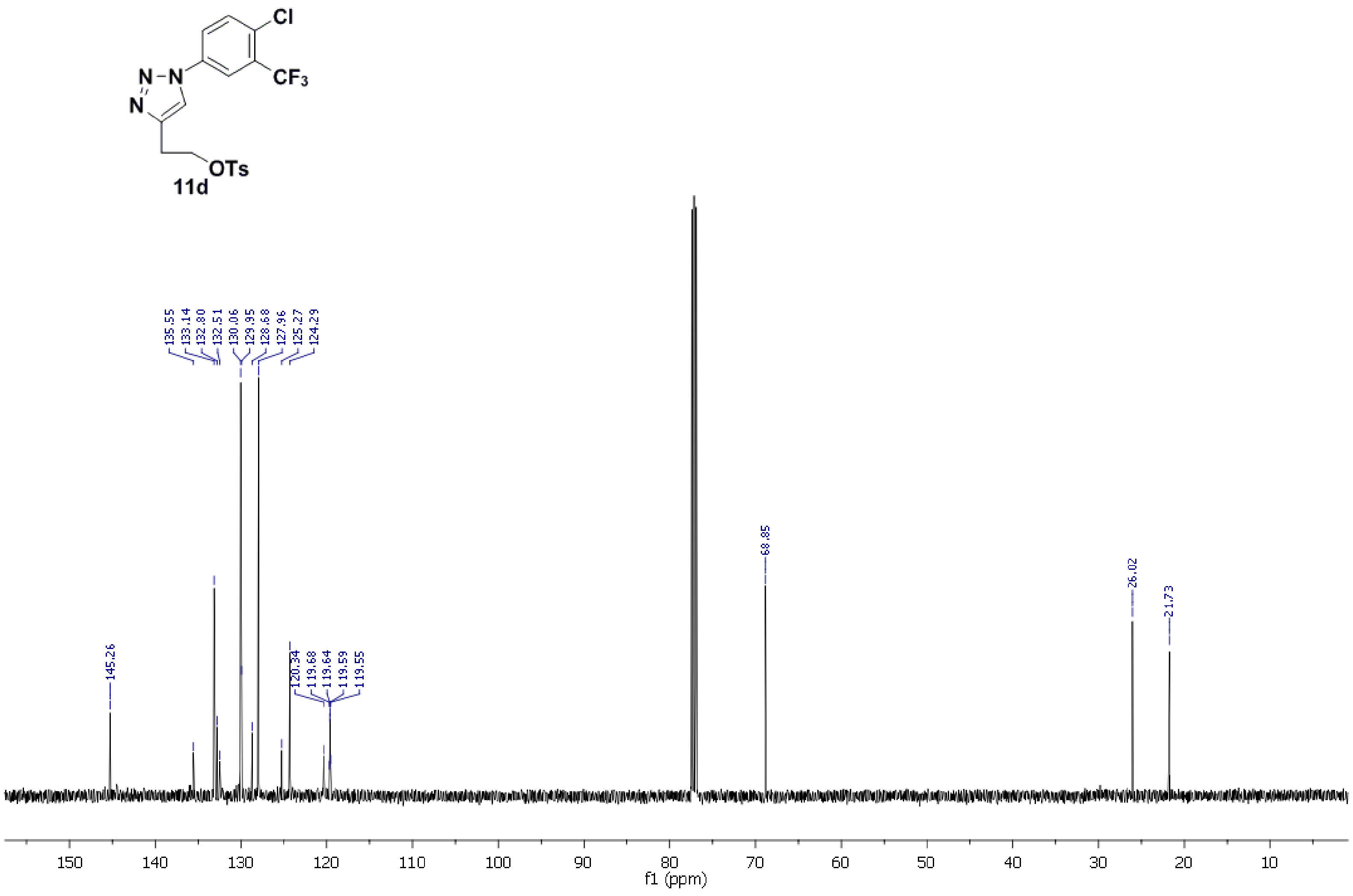

Figure S10. ^{13}C-NMR (125 MHz, $CDCl_3$) of compound **11d**.

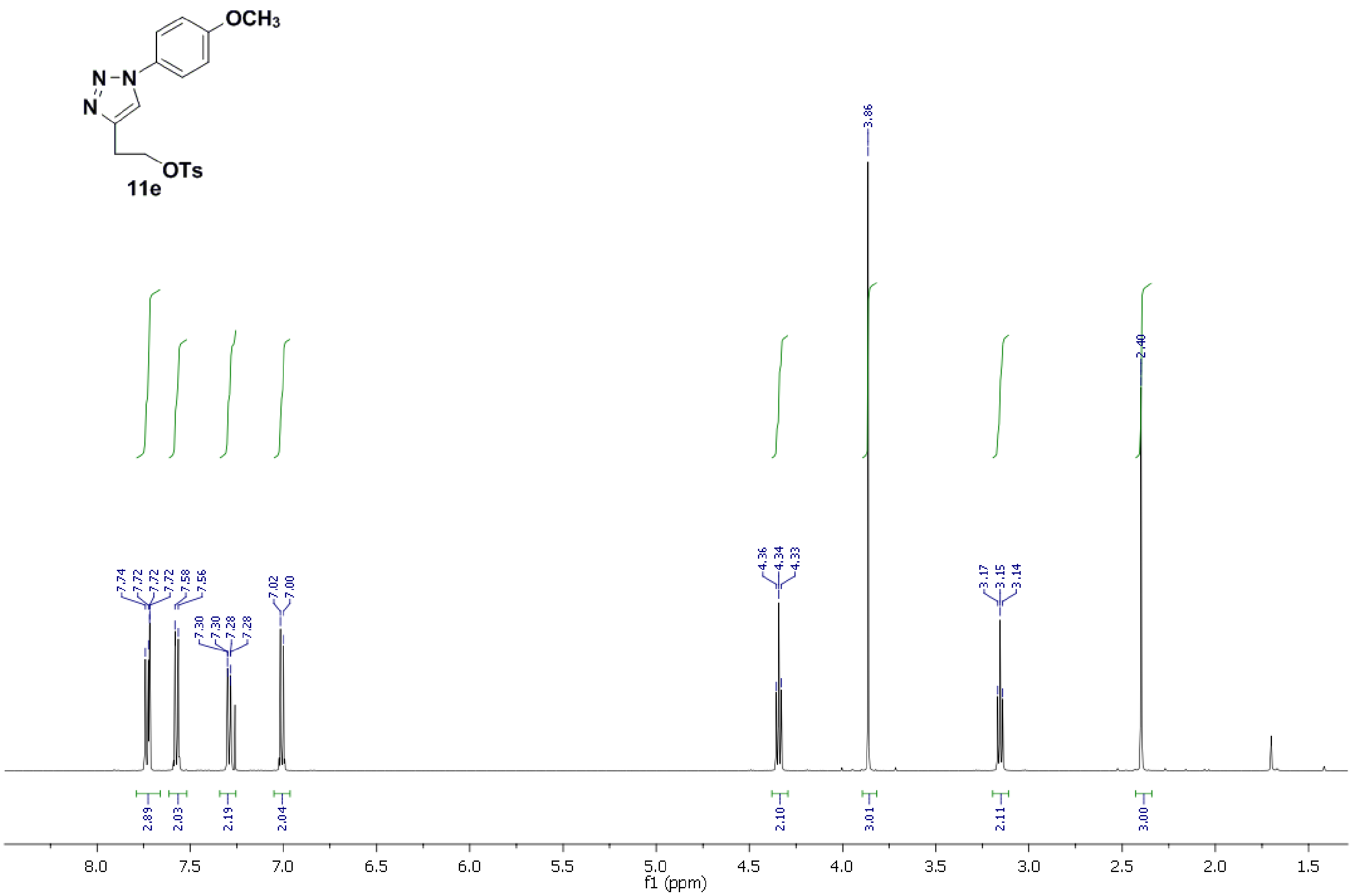

Figure S11. ^{1}H-NMR (500 MHz, $CDCl_3$) of compound **11e**.

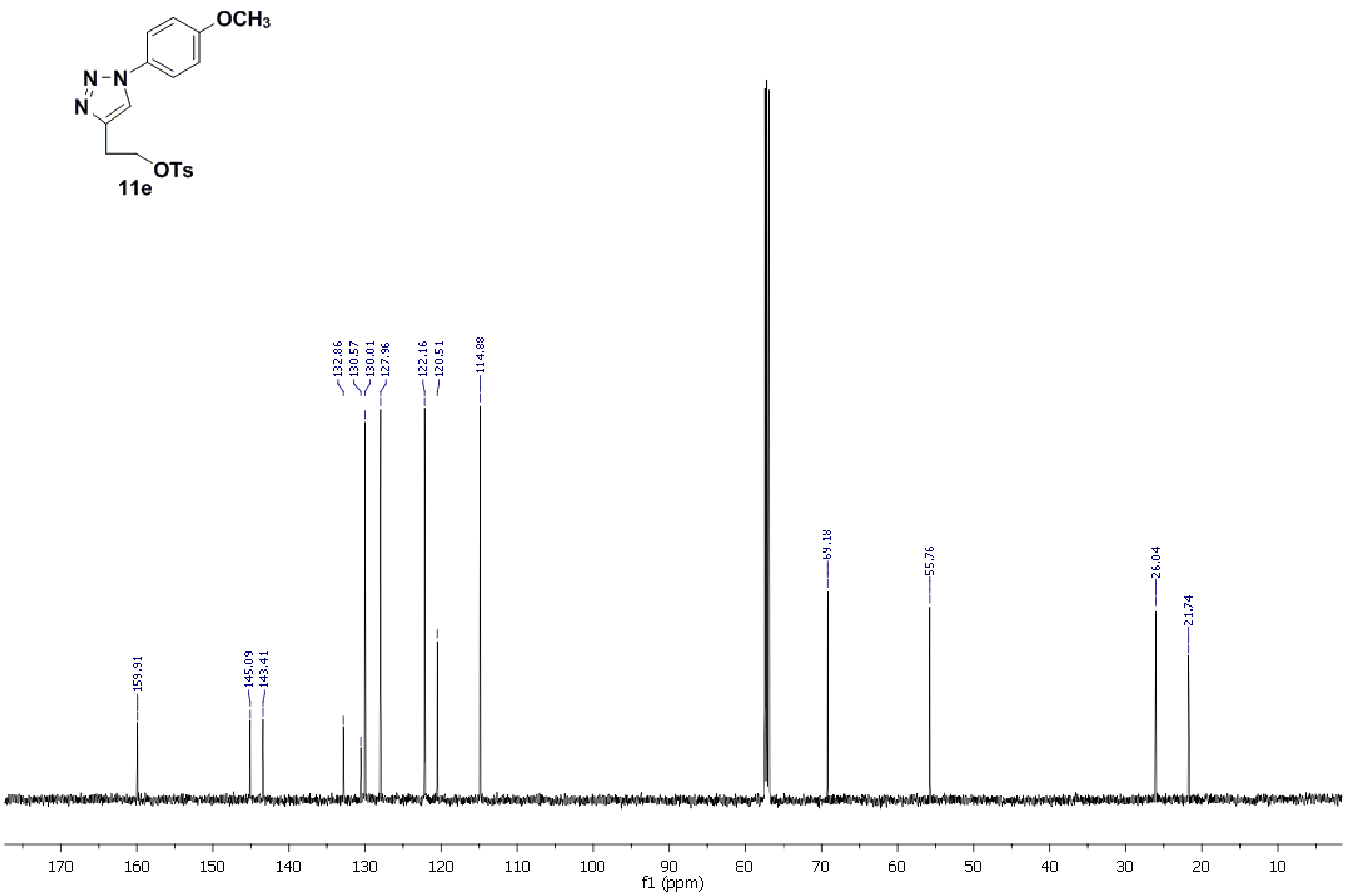

Figure S12. ^{13}C-NMR (125 MHz, $CDCl_3$) of compound **11e**.

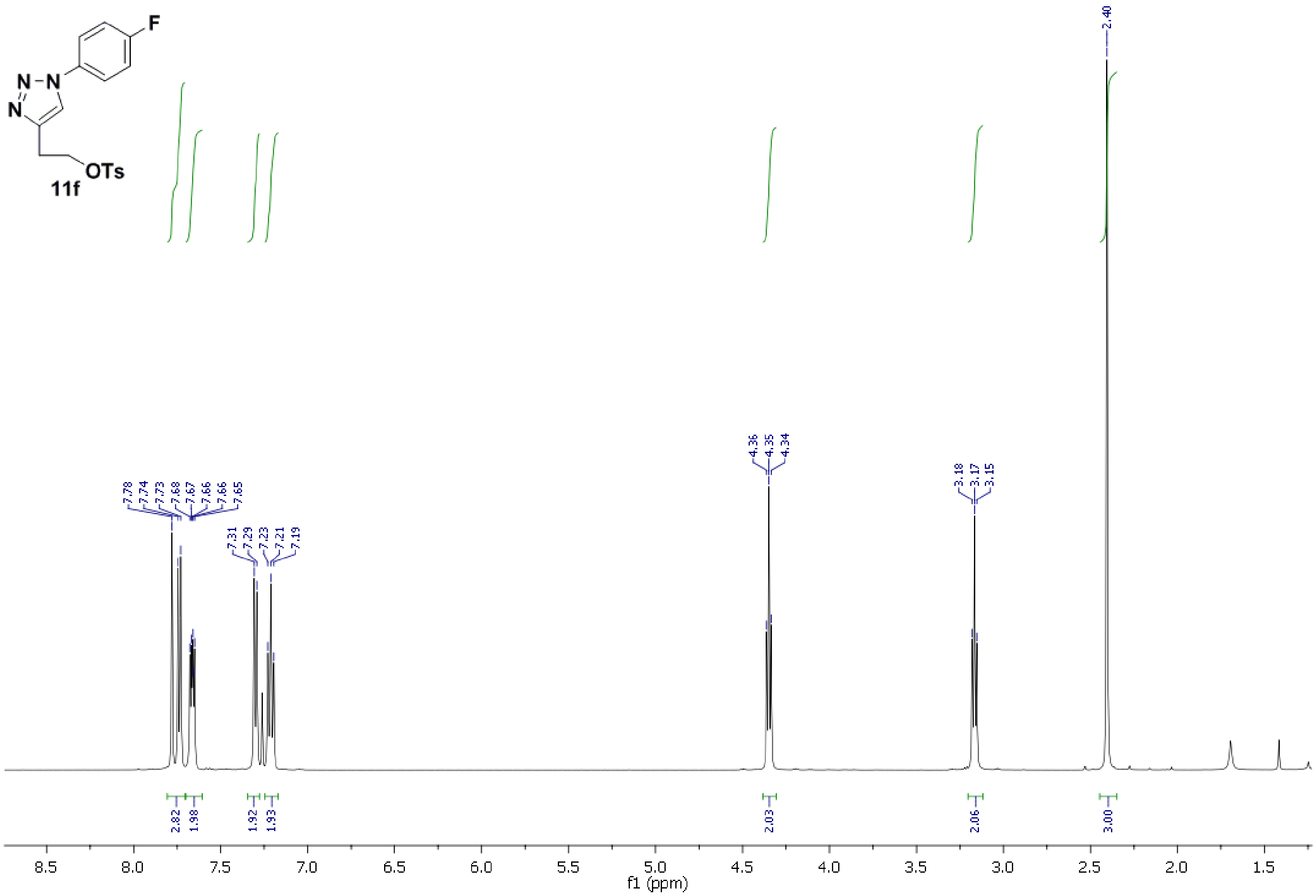

Figure S13. ^{1}H-NMR (500 MHz, $CDCl_3$) of compound **11f**.

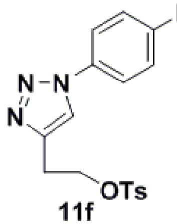

11f

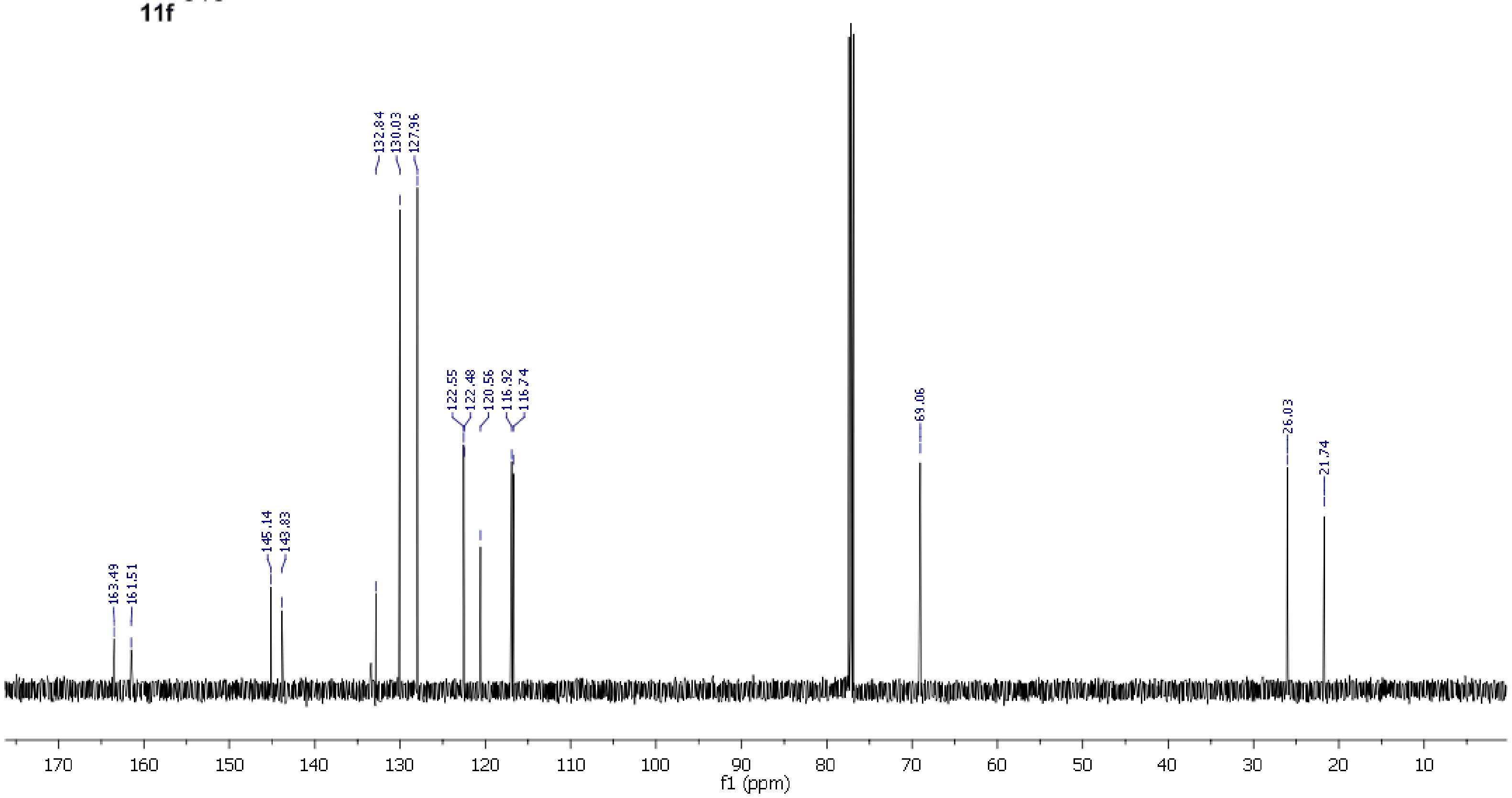

Figure S14. ^{13}C-NMR (125 MHz, $CDCl_3$) of compound **11f**.

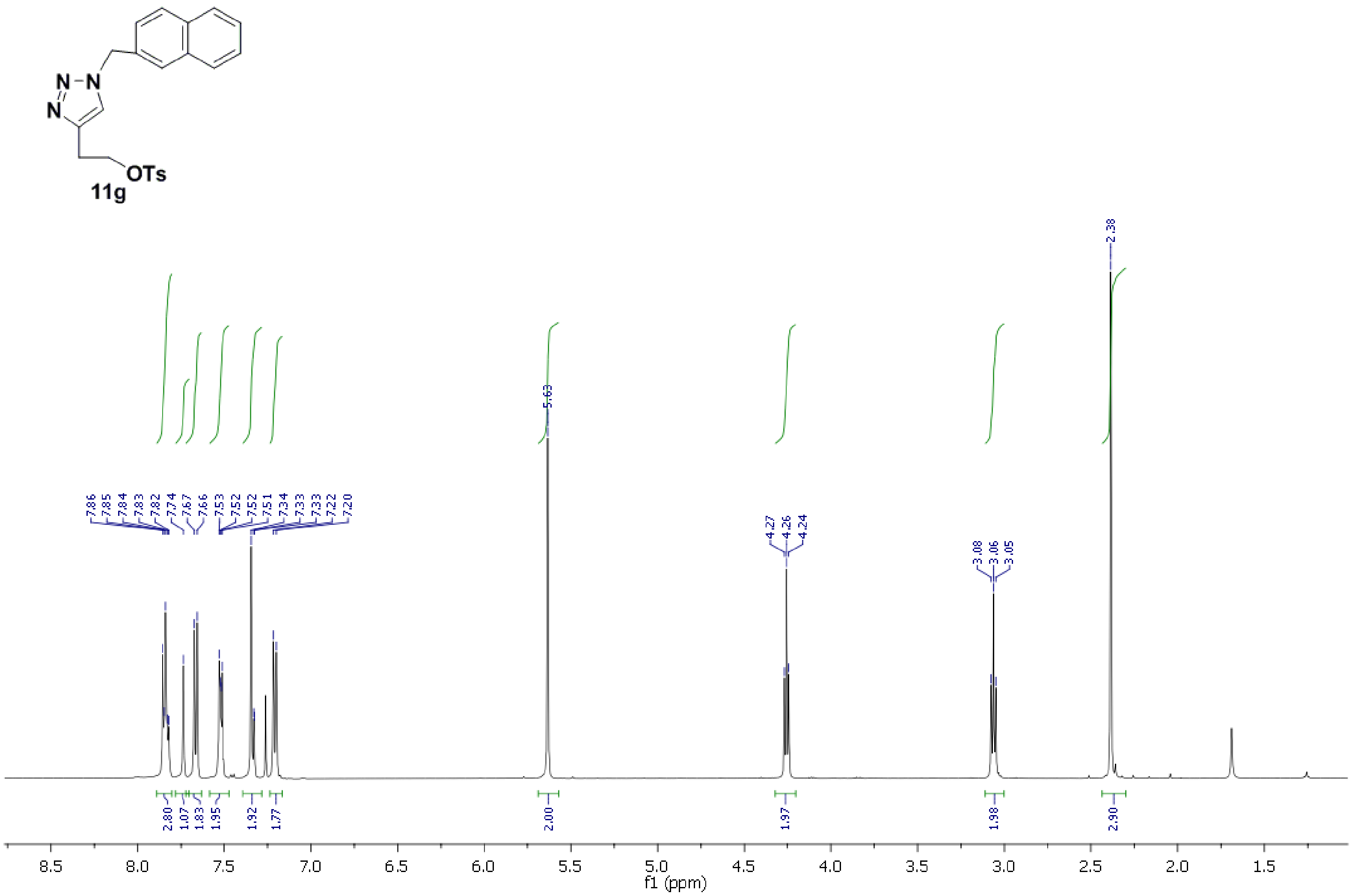

Figure S15. ^{1}H-NMR (500 MHz, $CDCl_3$) of compound **11g**.

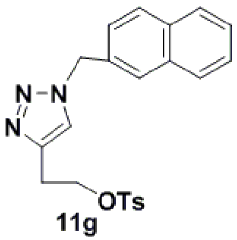

11g

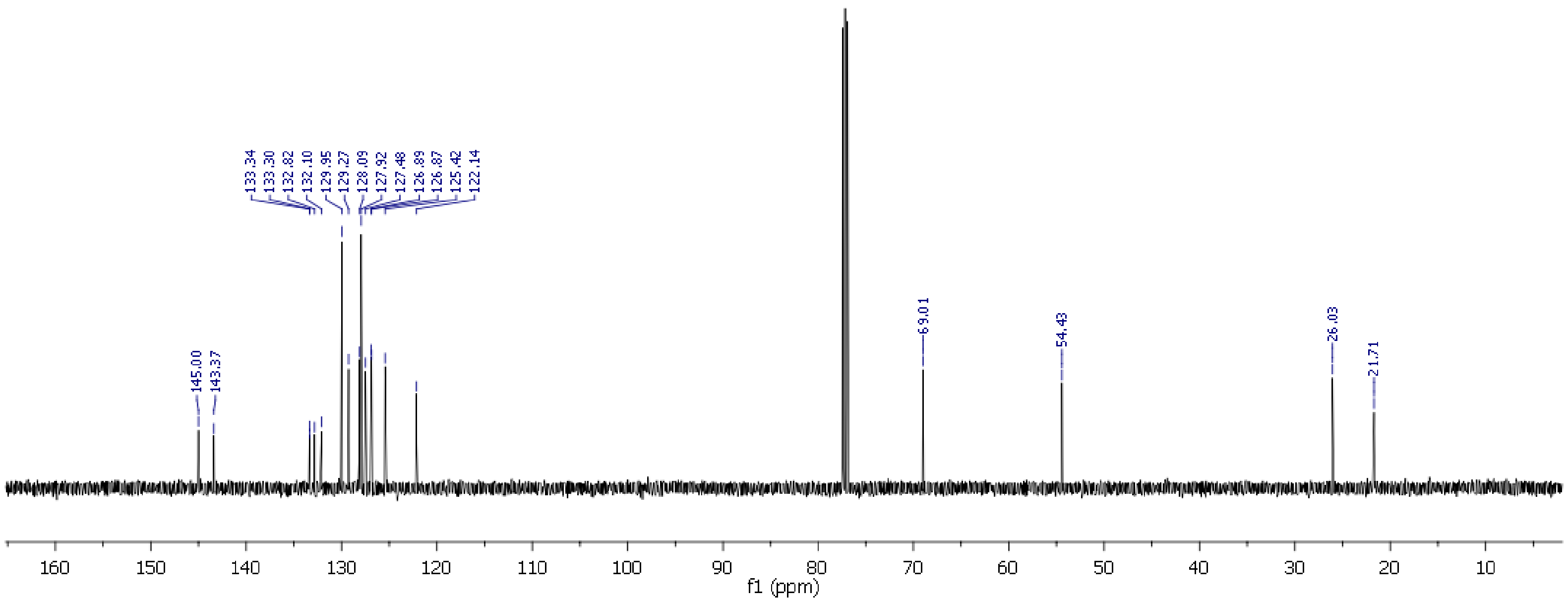

Figure S16. ^{13}C-NMR (125 MHz, $CDCl_3$) of compound **11g**.

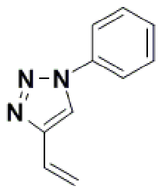

12a

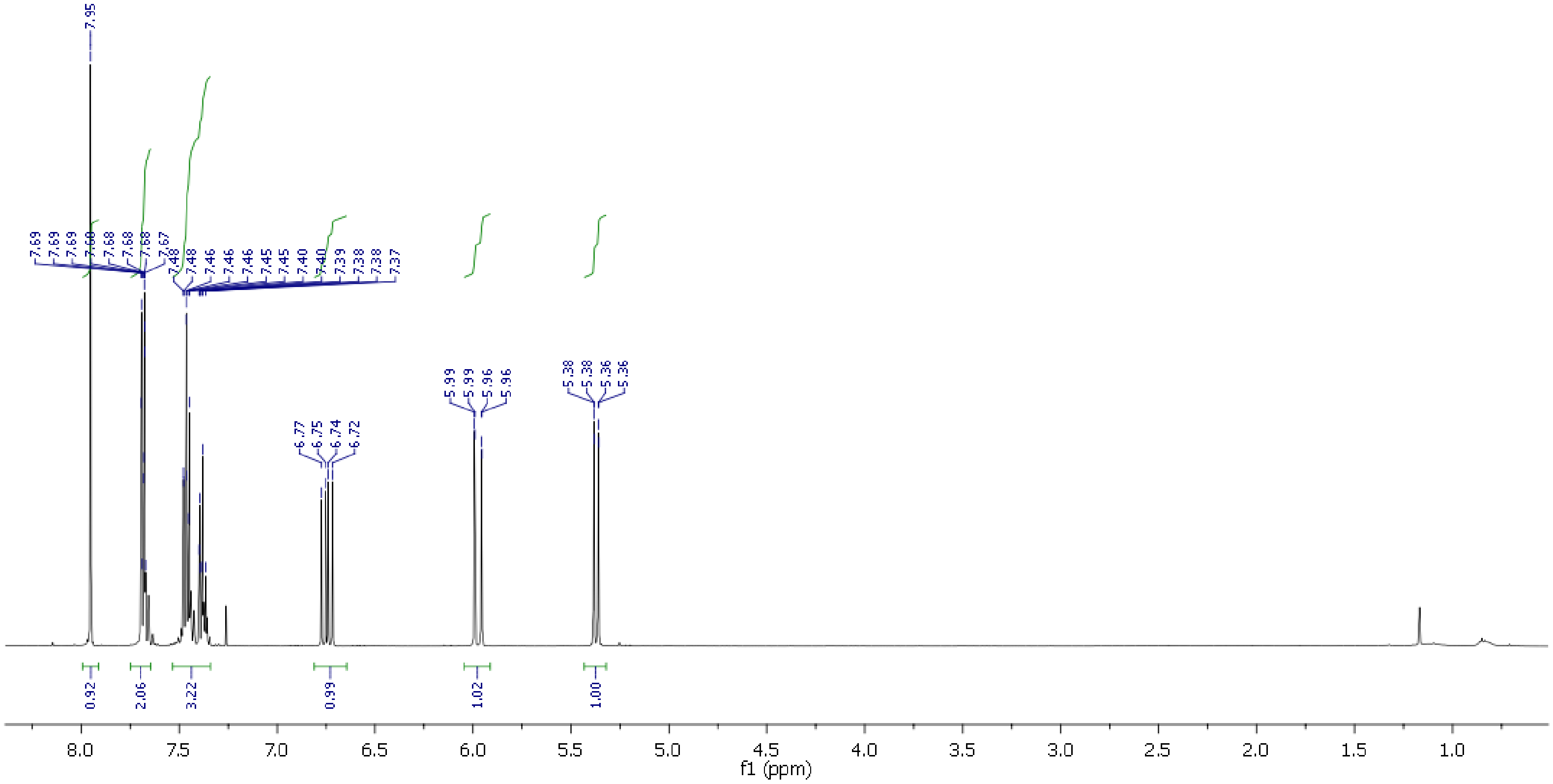

Figure S17. ^{1}H-NMR (500 MHz, $CDCl_3$) of compound **12a**.

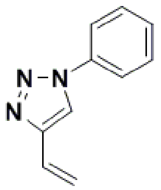

12a

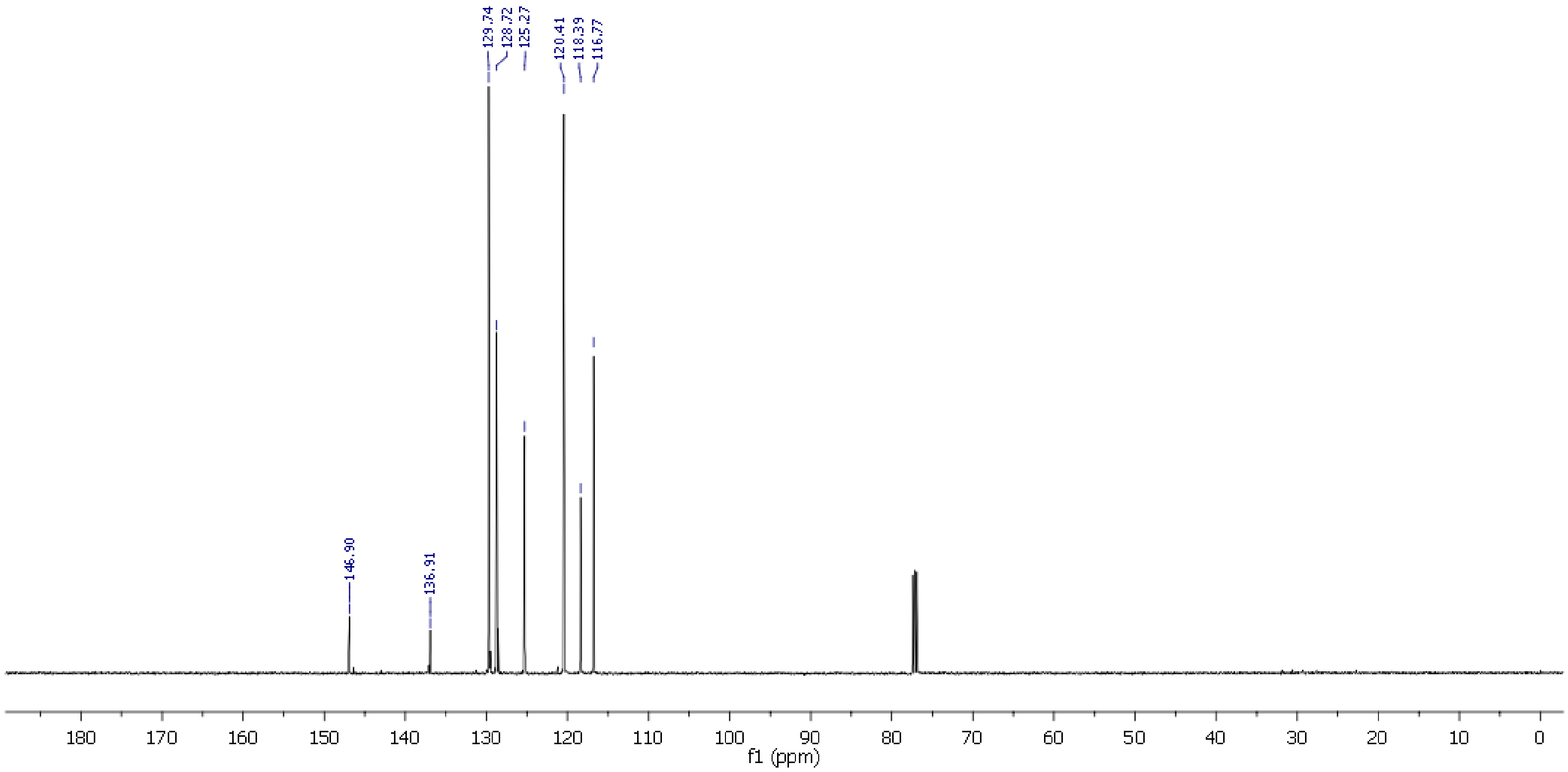

Figure S18. ^{13}C-NMR (125 MHz, $CDCl_3$) of compound **12a**.

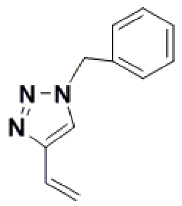

12b

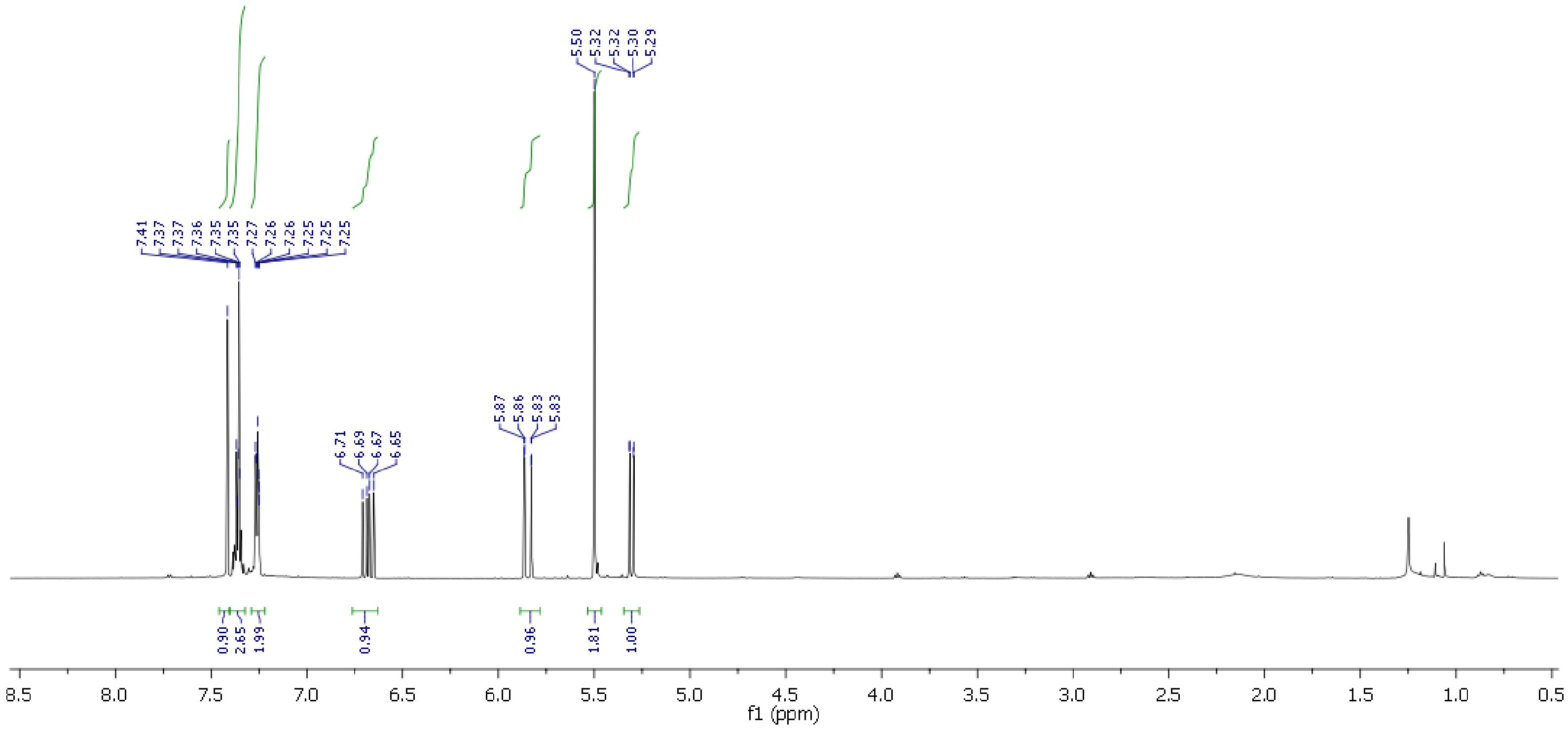

Figure S19. ¹H-NMR (500 MHz, $CDCl_3$) of compound **12b**.

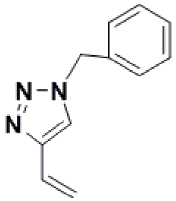

12b

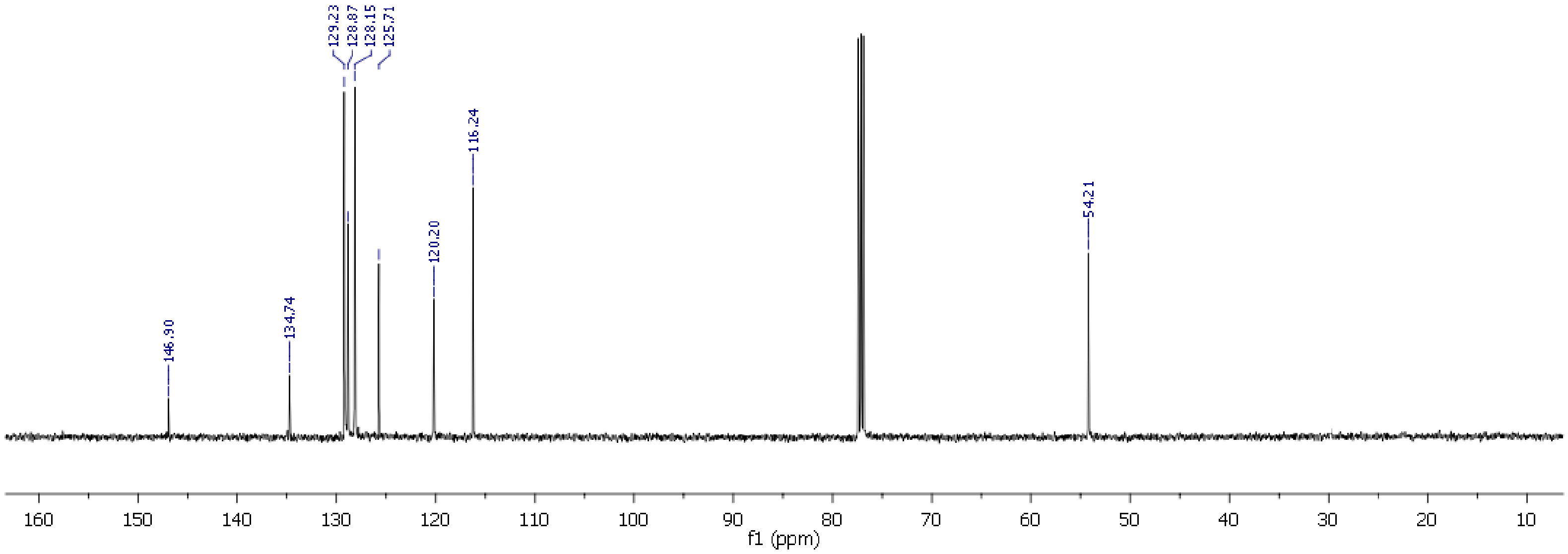

Figure S20. ^{13}C-NMR (125 MHz, $CDCl_3$) of compound **12b**.

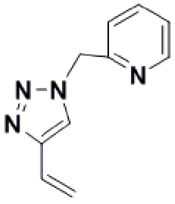

12c

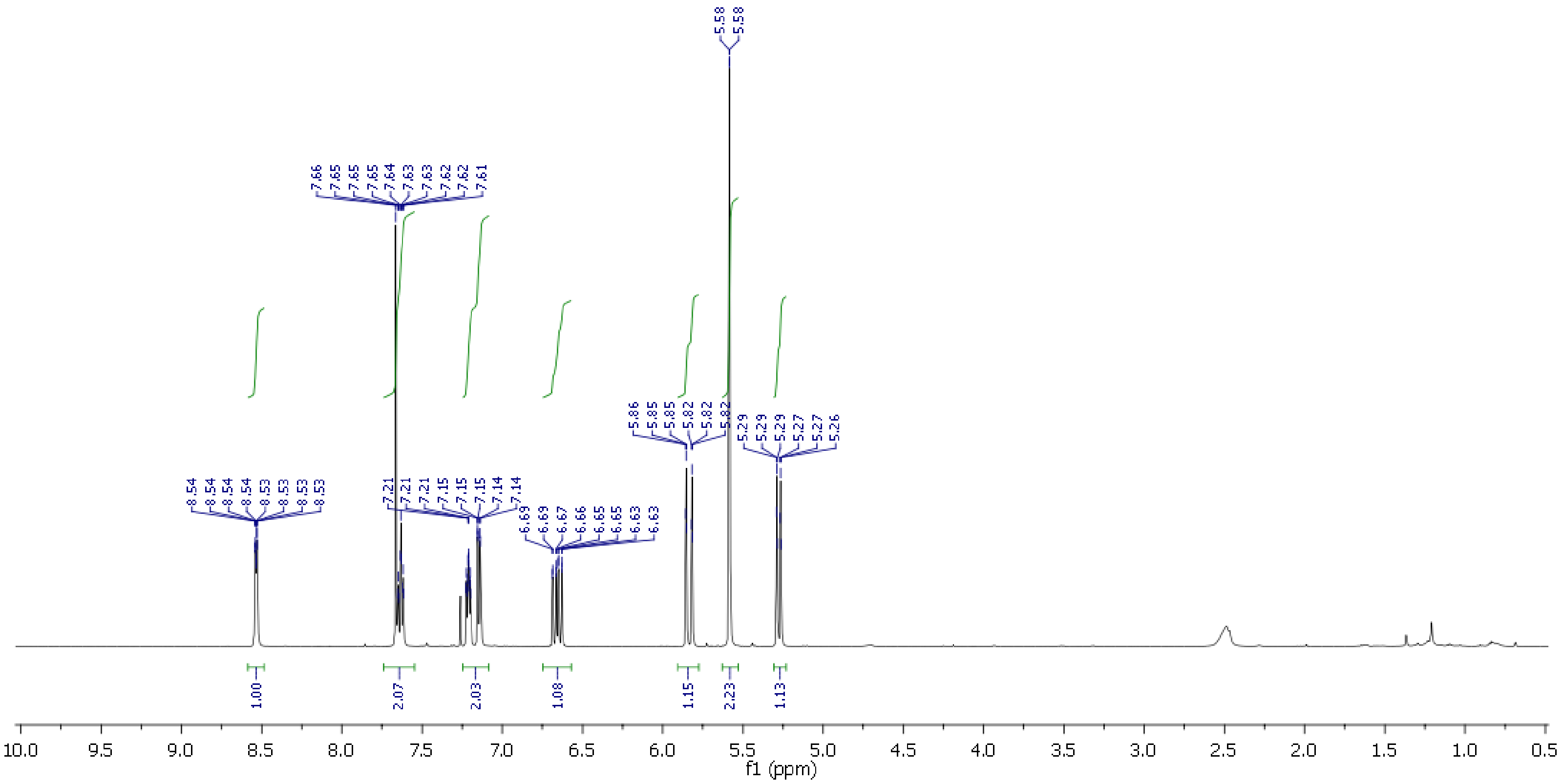

Figure S21. ^{1}H-NMR (500 MHz, $CDCl_3$) of compound **12c**.

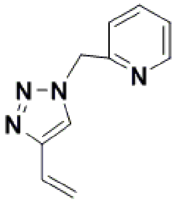

12c

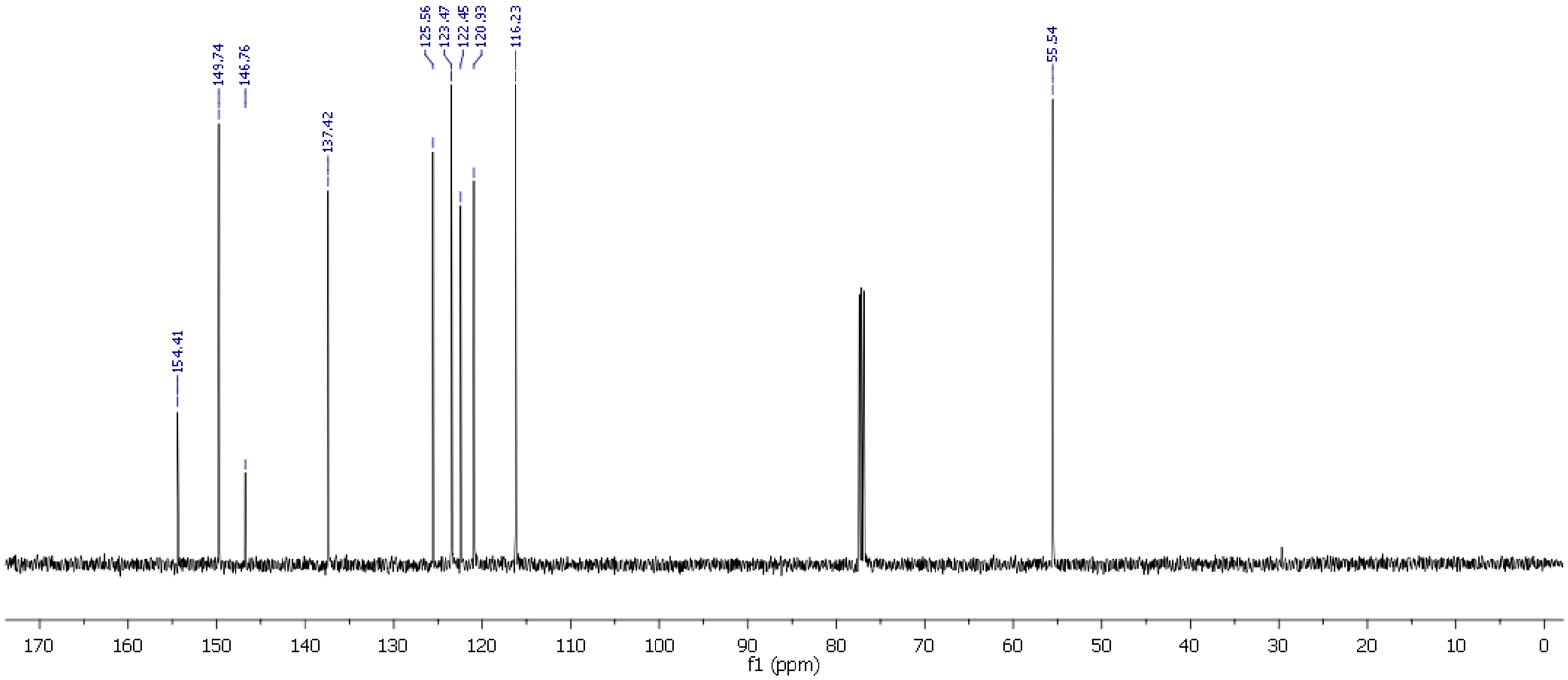

Figure S22. ^{13}C-NMR (125 MHz, $CDCl_3$) of compound **12c**.

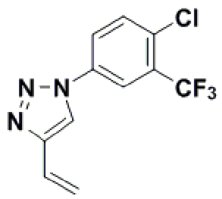

12d

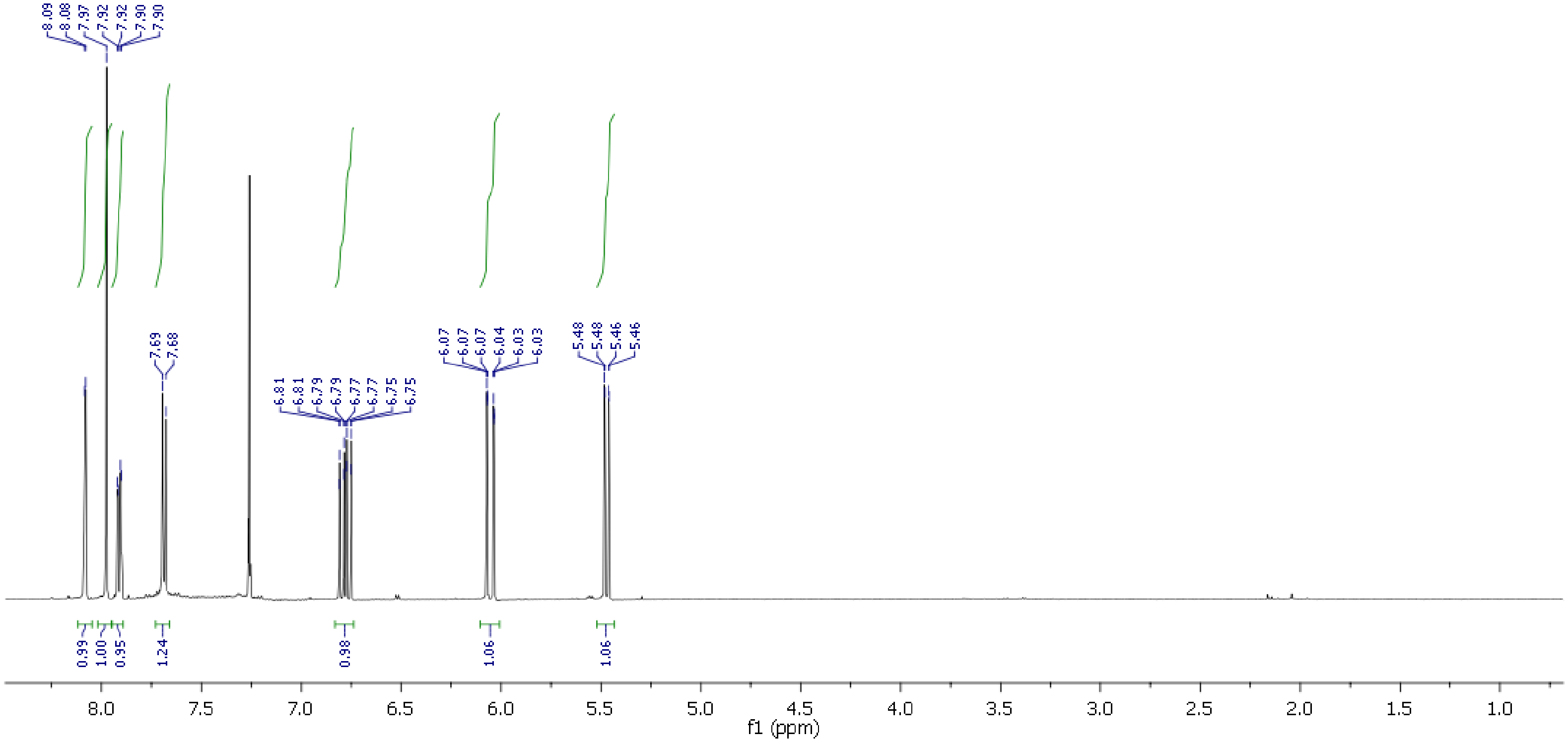

Figure S23. ^{1}H-NMR (500 MHz, $CDCl_3$) of compound **12d**.

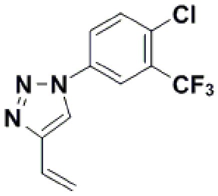

12d

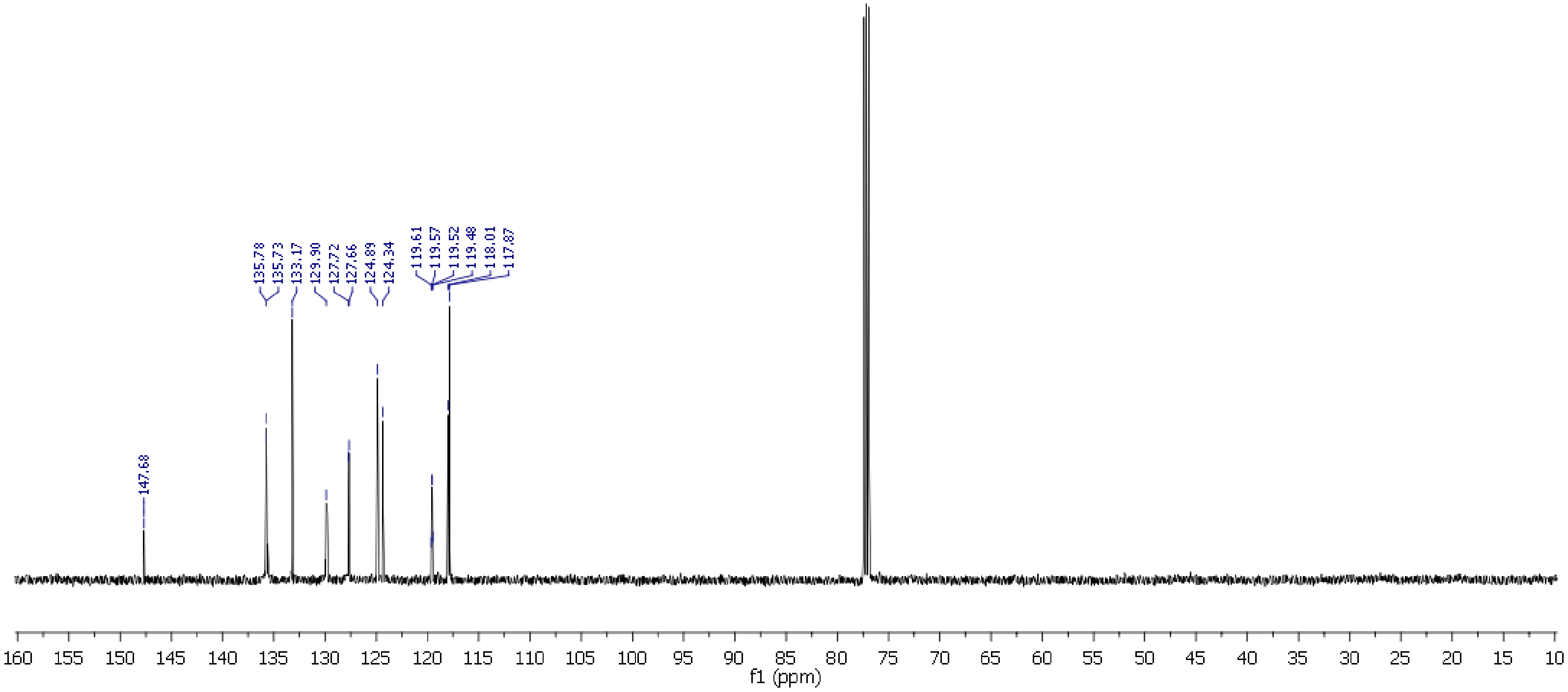

Figure S24. ^{13}C-NMR (125 MHz, $CDCl_3$) of compound **12d**.

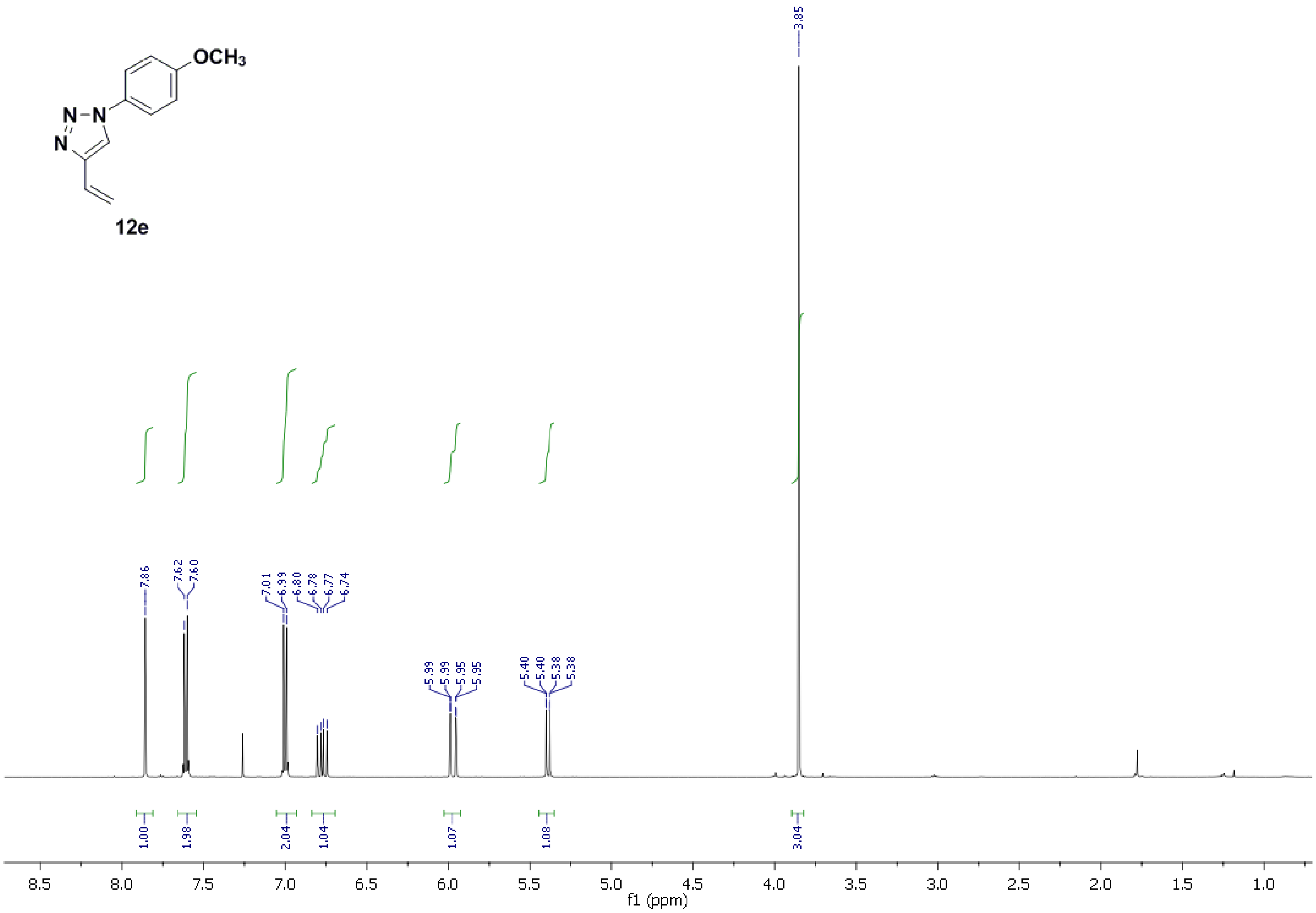

Figure S25. ^{1}H-NMR (500 MHz, $CDCl_3$) of compound **12e**.

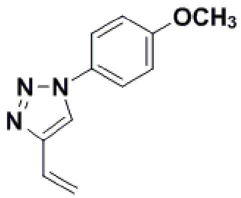

12e

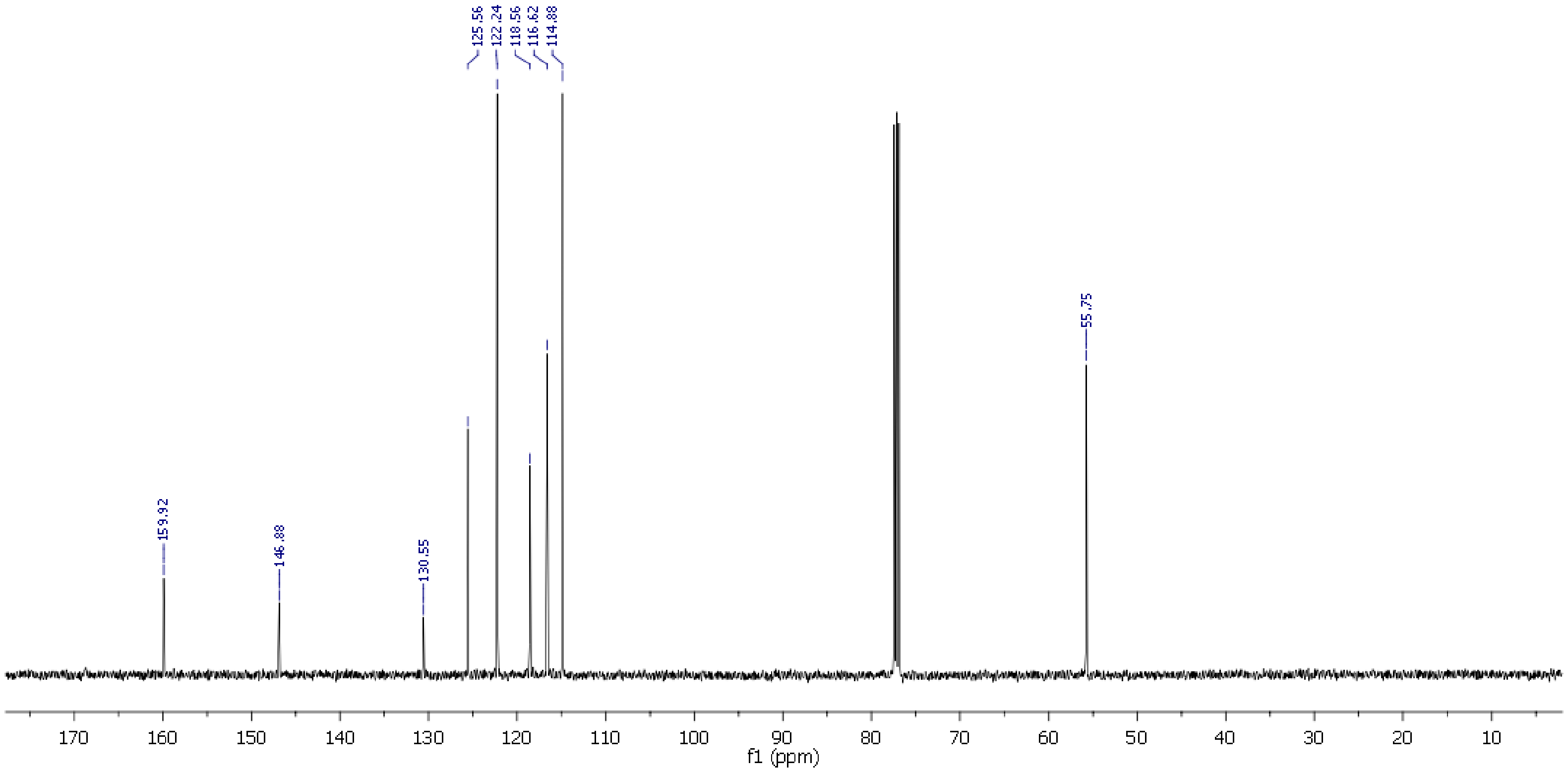

Figure S26. ^{13}C-NMR (125 MHz, $CDCl_3$) of compound **12e**.

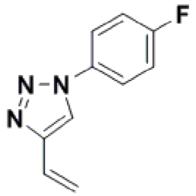

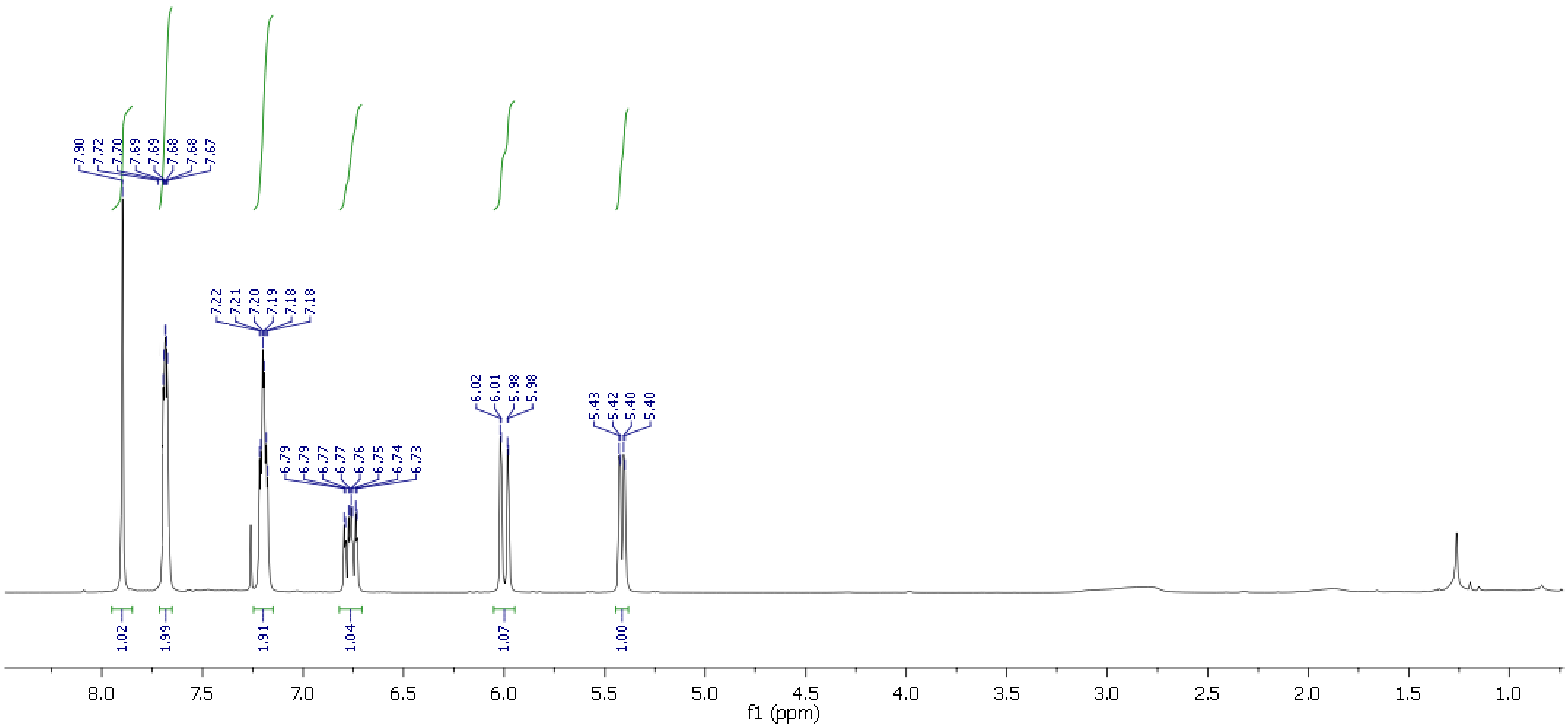

Figure S27. ^{1}H-NMR (500 MHz, $CDCl_3$) of compound **12f**.

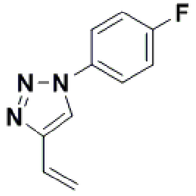

12f

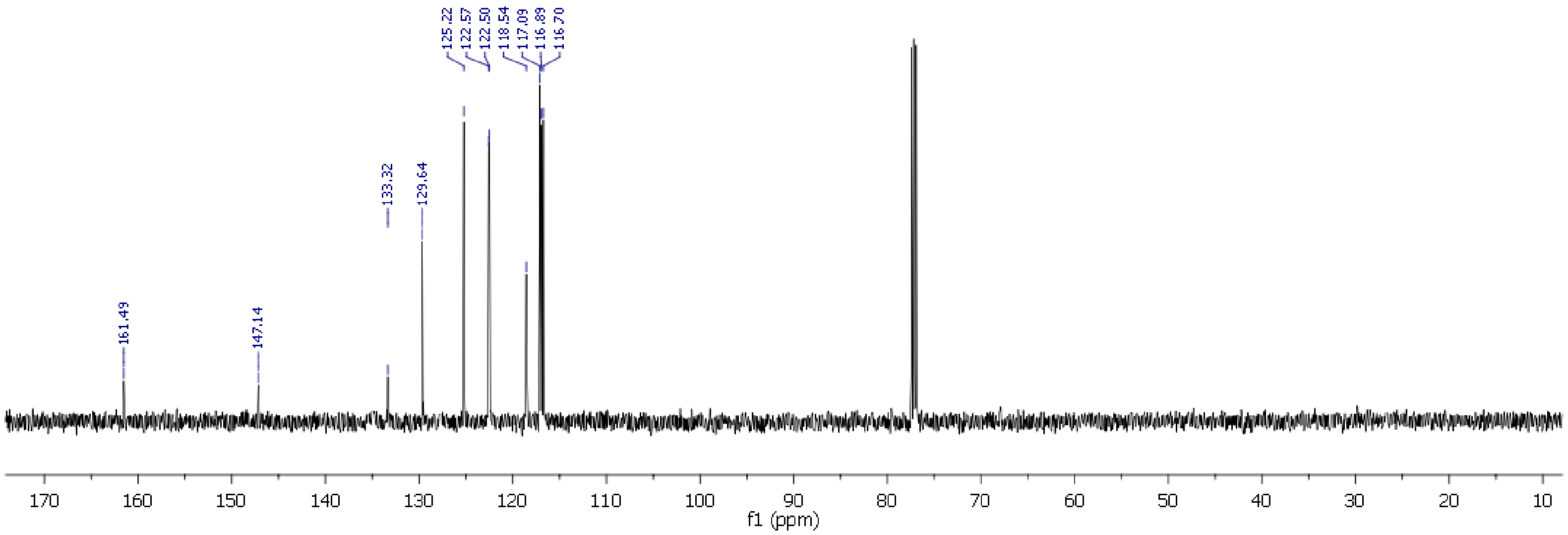

Figure S28. ^{13}C-NMR (125 MHz, $CDCl_3$) of compound **12f**.

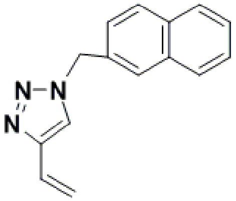

12g

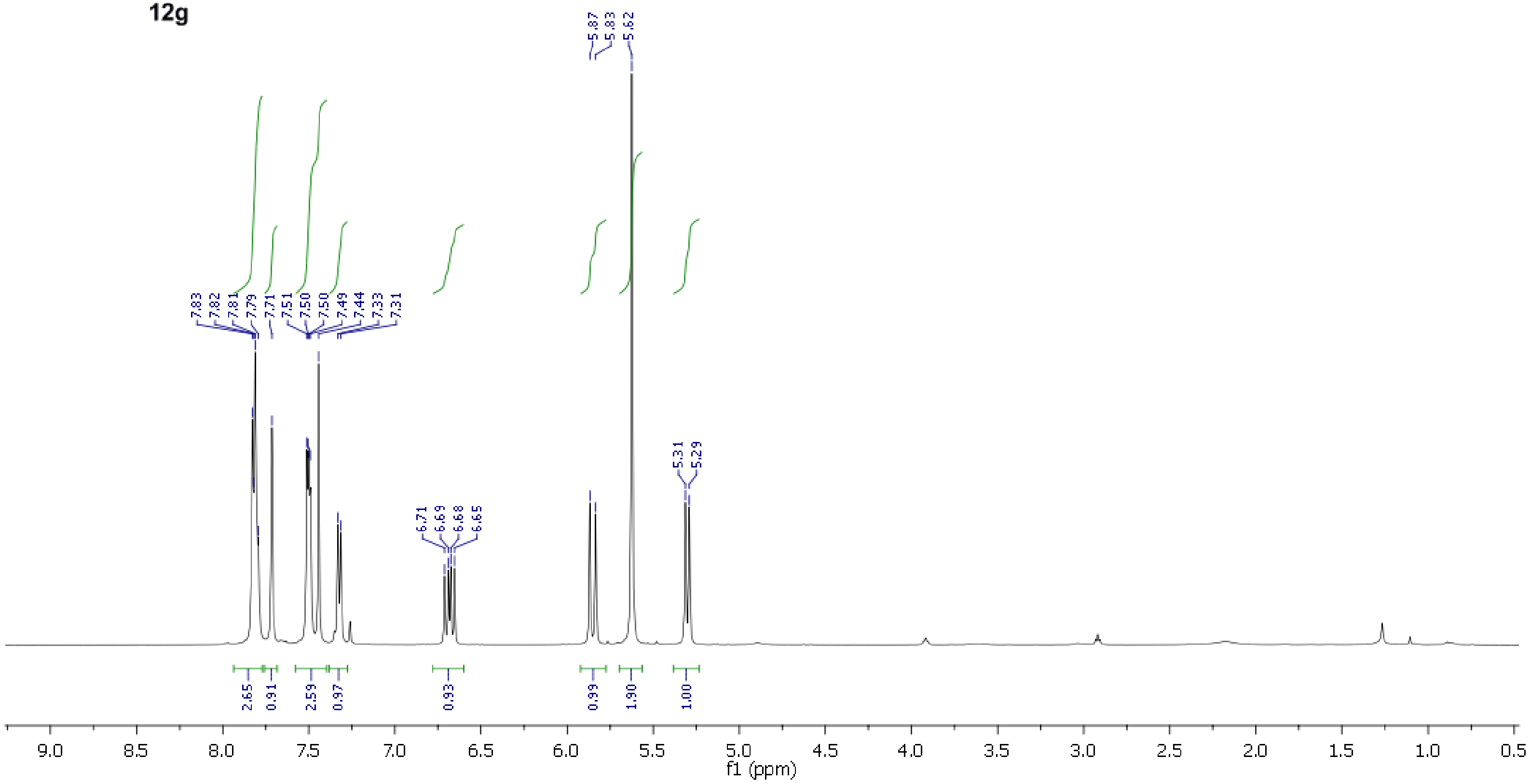

Figure S29. ^{1}H-NMR (500 MHz, $CDCl_3$) of compound **12g**.

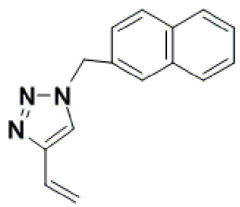

12g

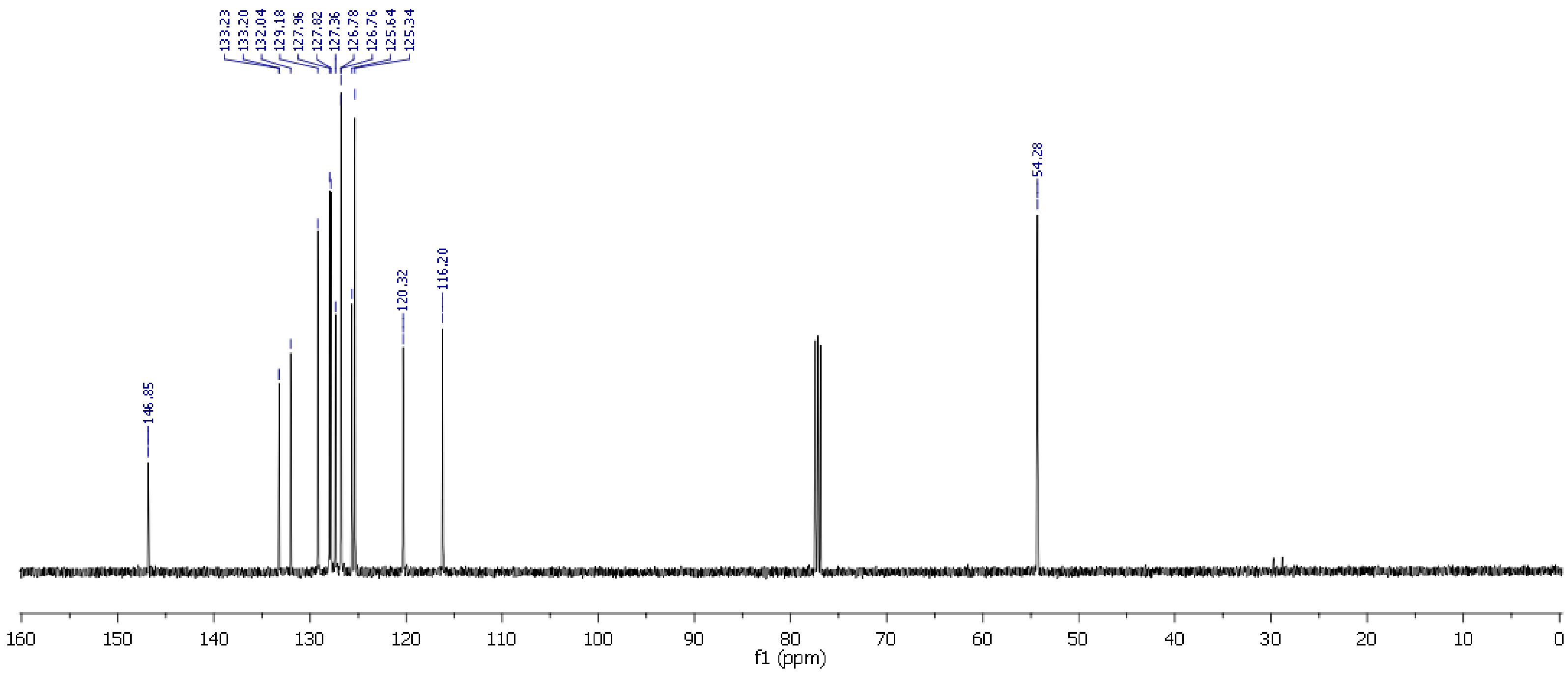

Figure S30. ^{13}C-NMR (125 MHz, $CDCl_3$) of compound **12g**.

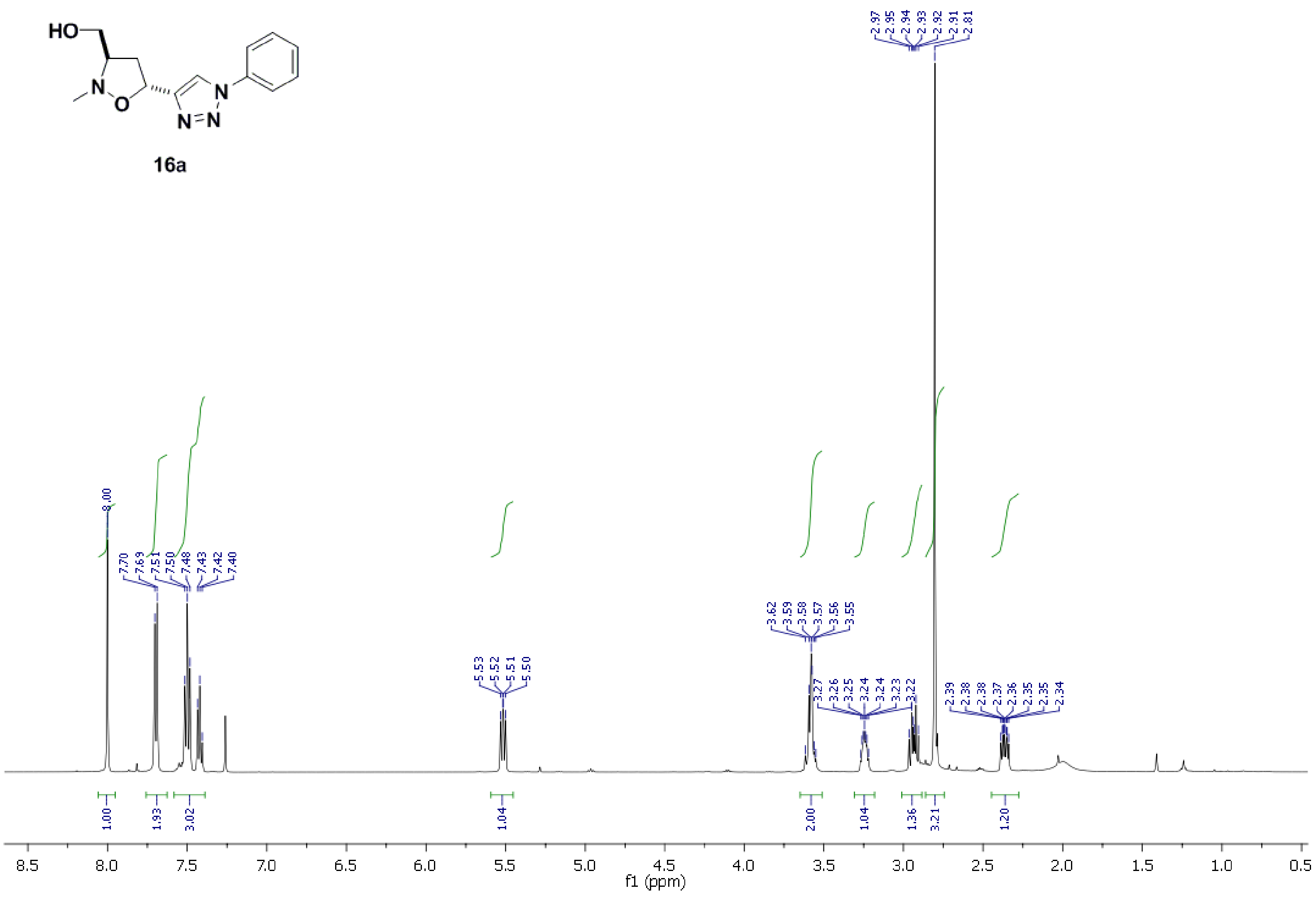

Figure S31. ^{1}H-NMR (500 MHz, $CDCl_3$) of compound **16a**.

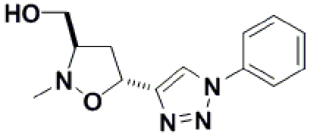

16a

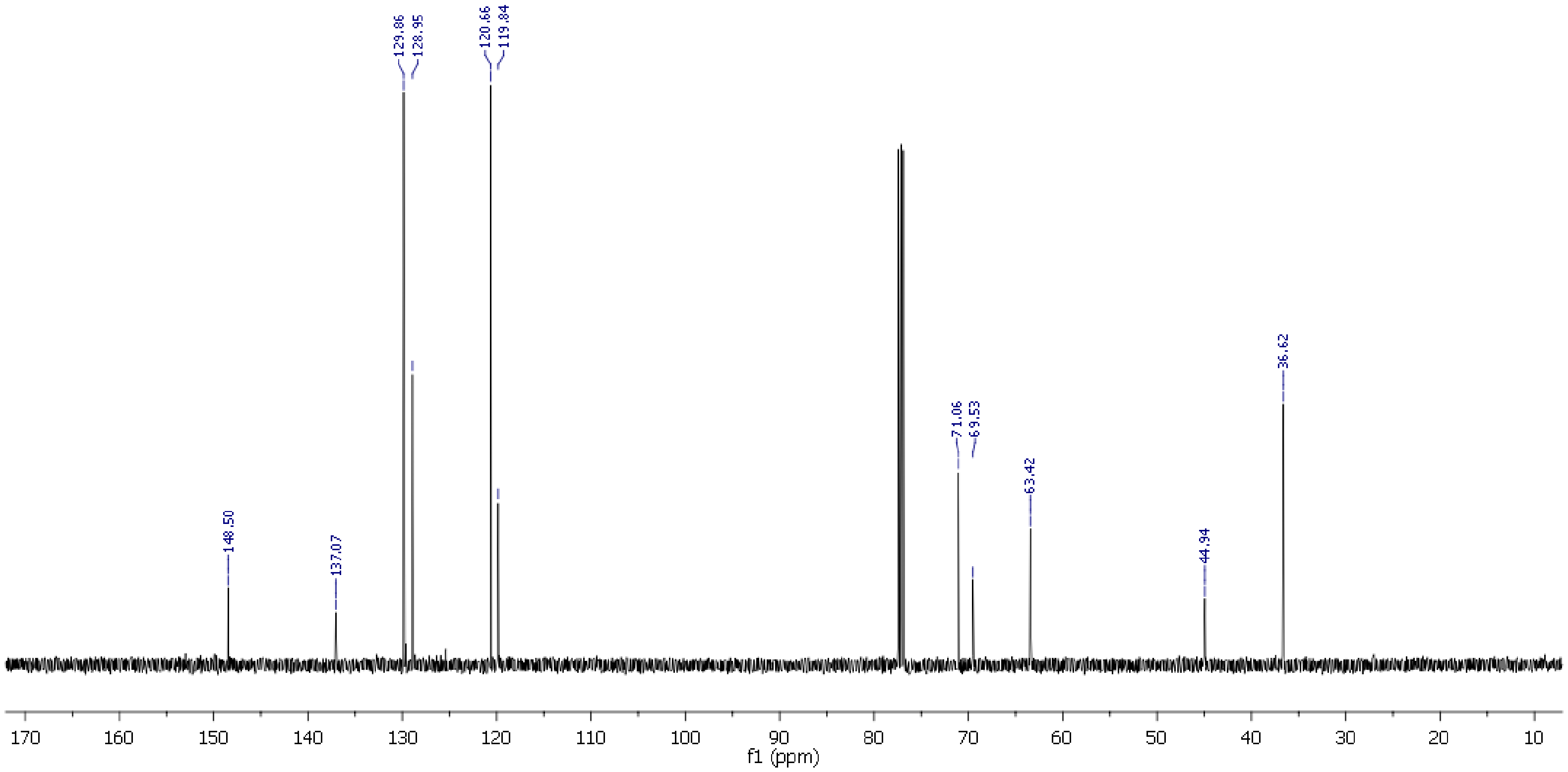

Figure S32. ^{13}C-NMR (125 MHz, $CDCl_3$) of compound **16a**.

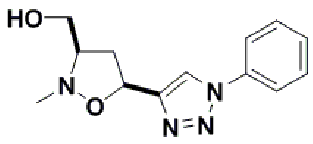

17a

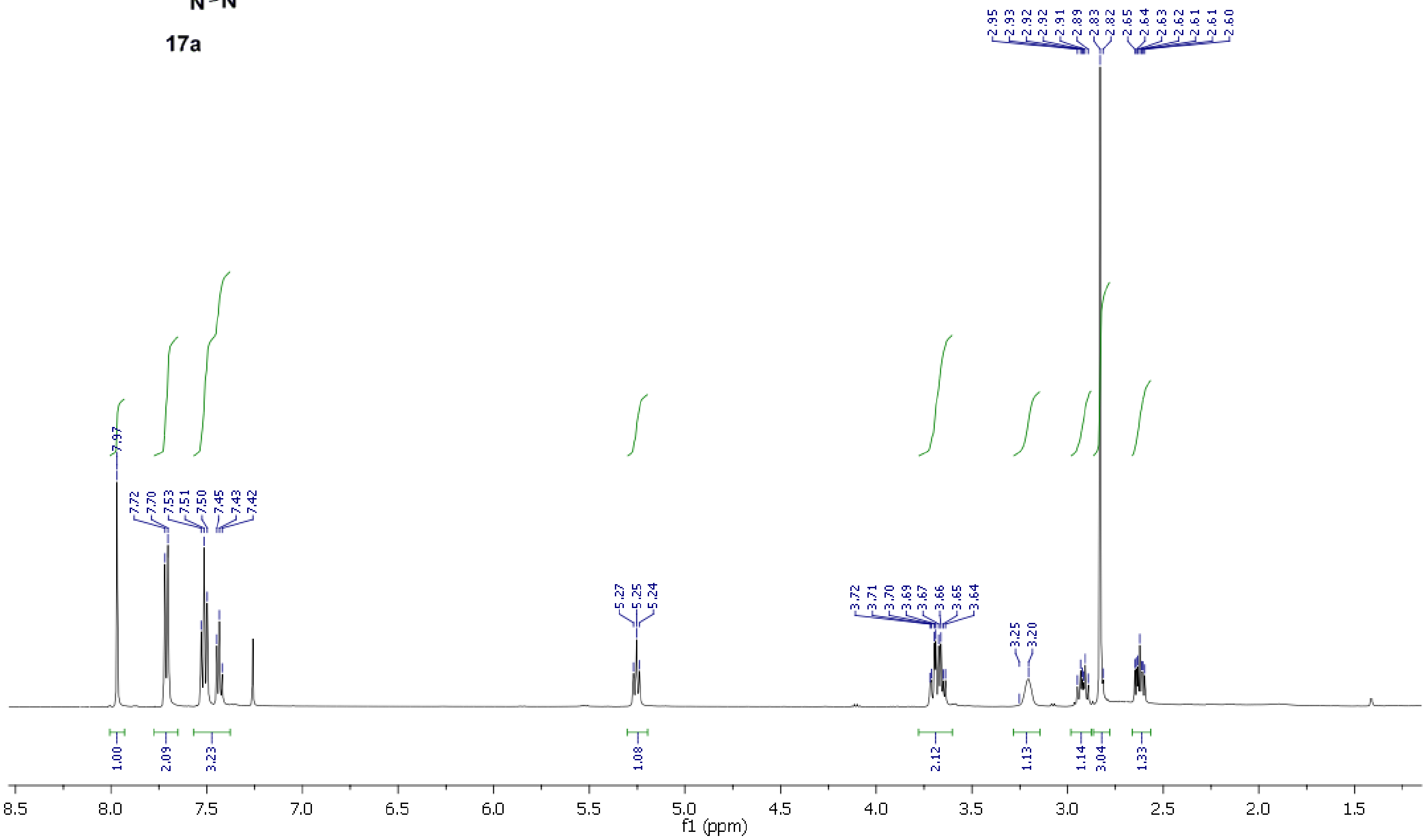

Figure S33. ^{1}H-NMR (500 MHz, $CDCl_3$) of compound **17a**.

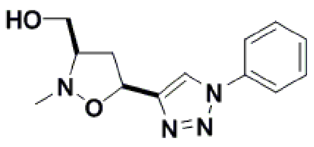

17a

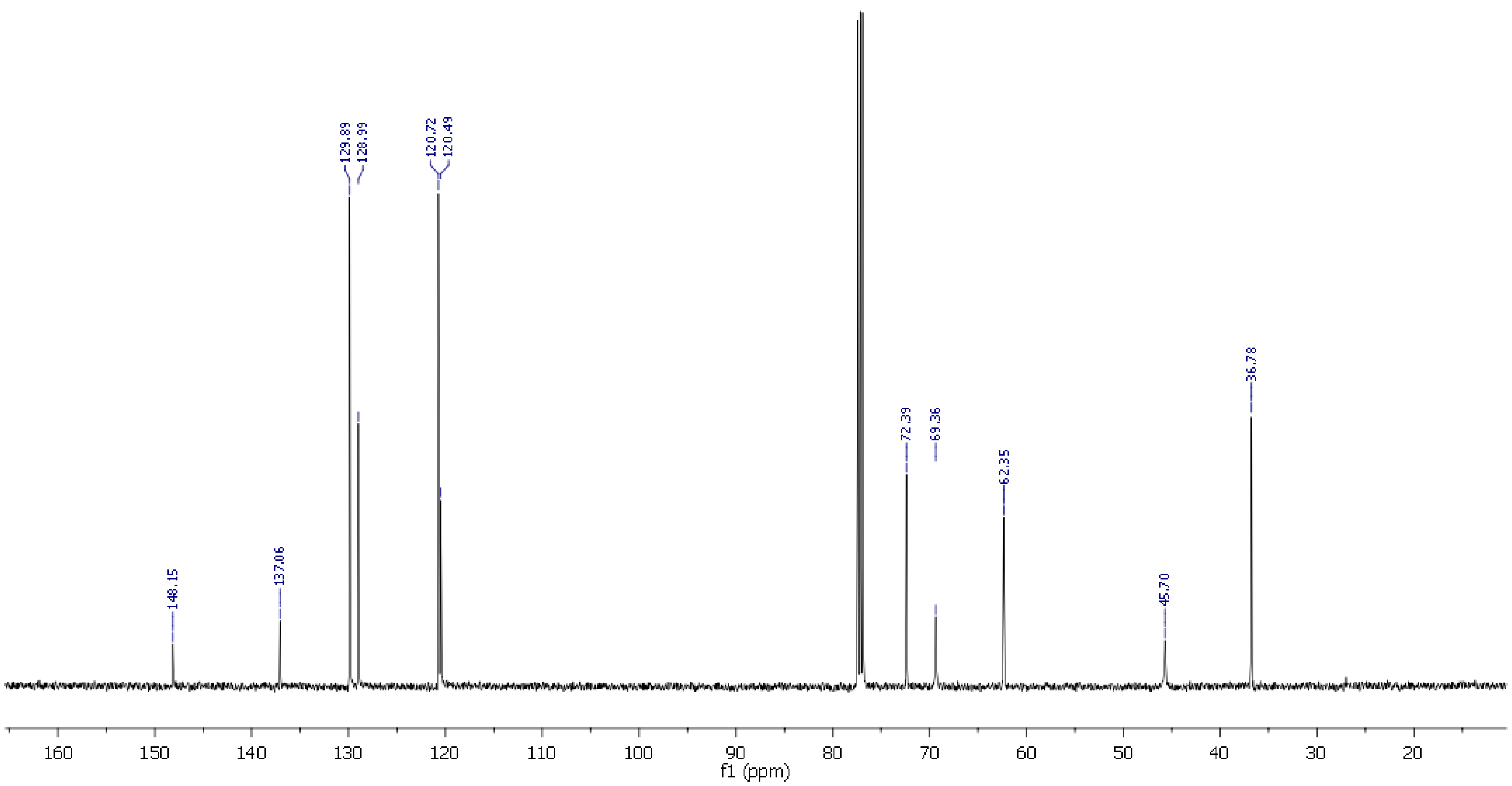

Figure S34. ^{13}C-NMR (125 MHz, $CDCl_3$) of compound **17a**.

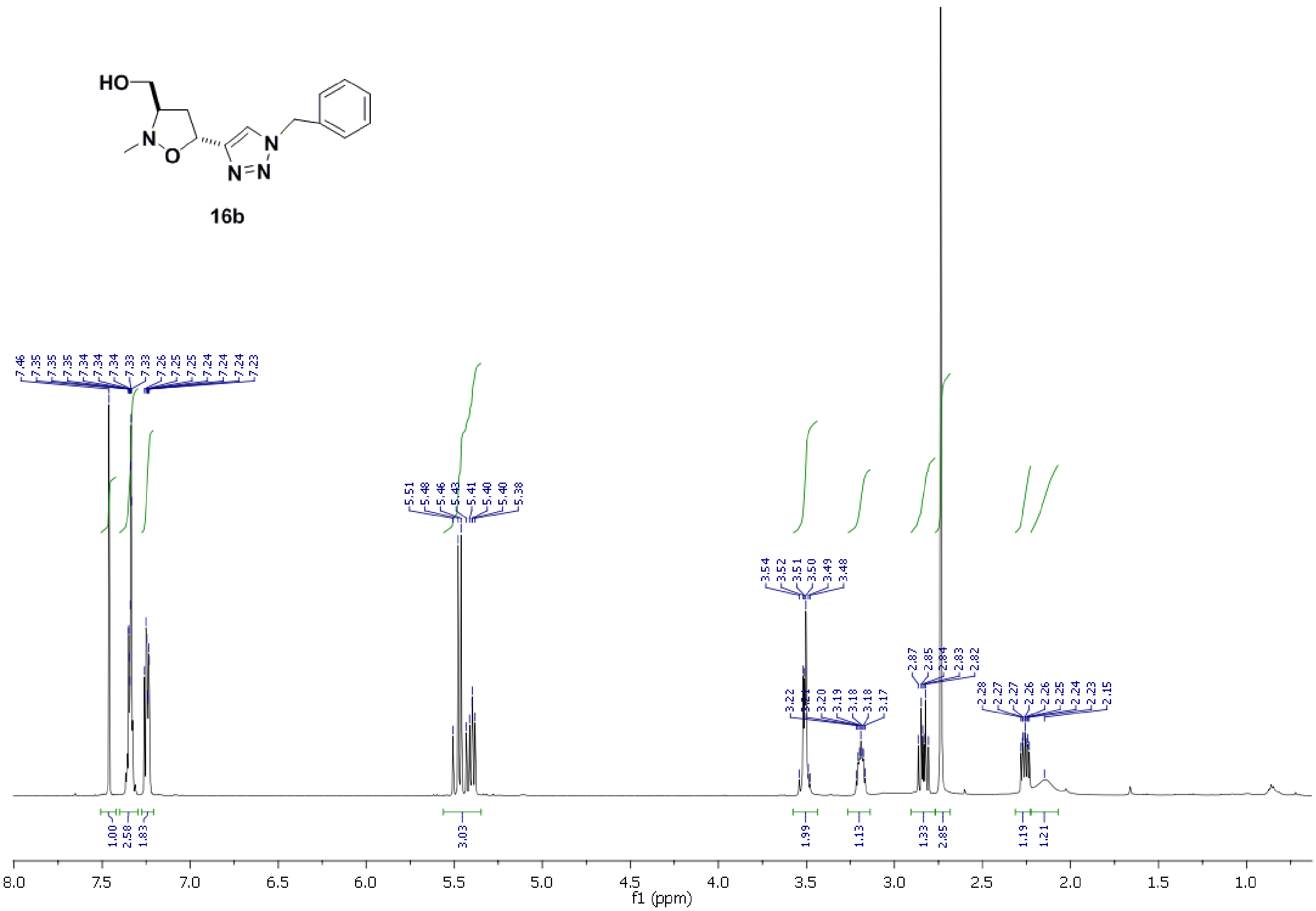

Figure S35. ^{1}H-NMR (500 MHz, $CDCl_3$) of compound **16b**.

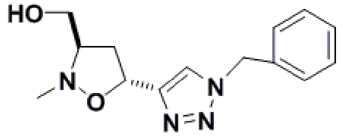

16b

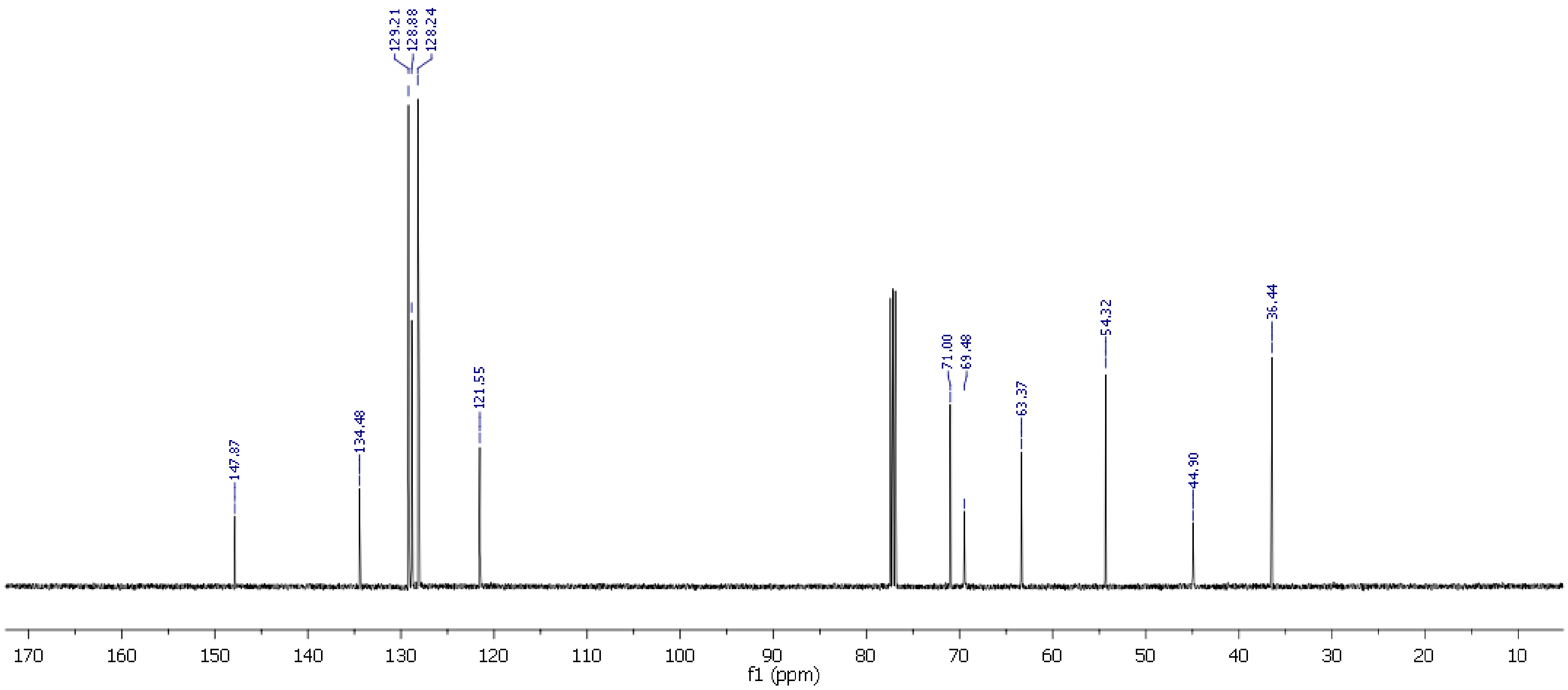

Figure S36. ^{13}C-NMR (125 MHz, $CDCl_3$) of compound **16b**.

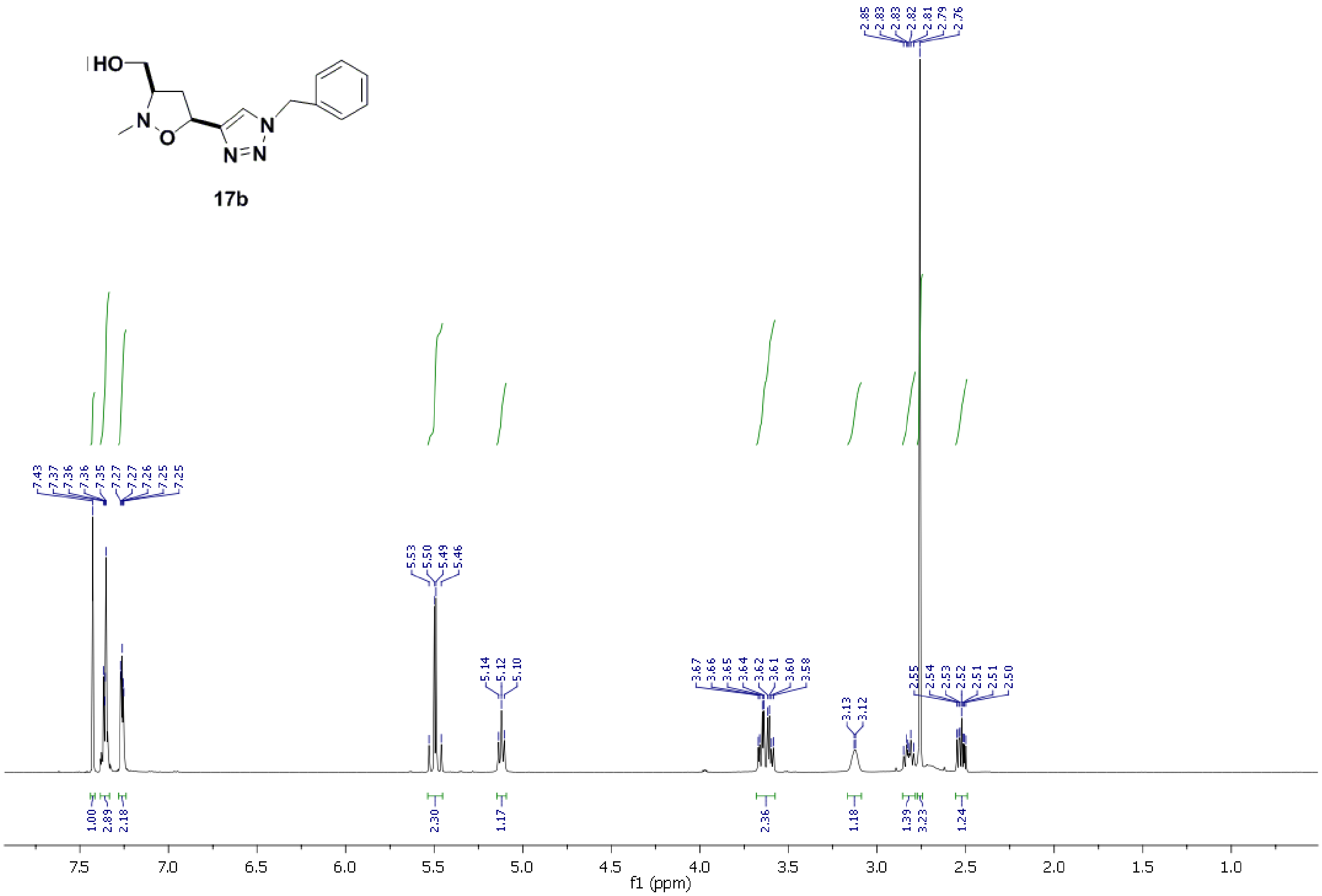

Figure S37. ^{1}H-NMR (500 MHz, $CDCl_3$) of compound **17b**.

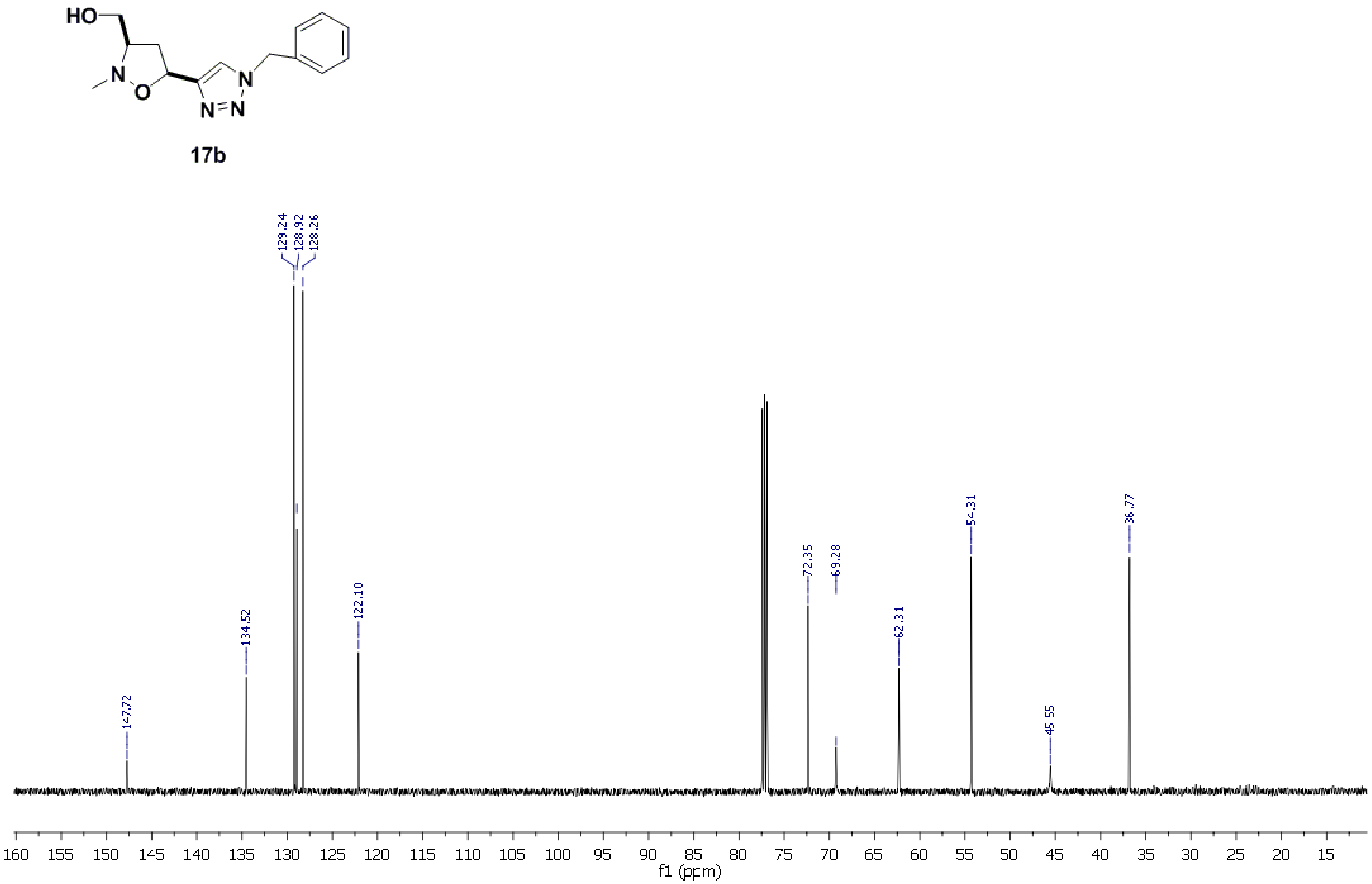

Figure S38. ^{13}C-NMR (125 MHz, $CDCl_3$) of compound **17b**.

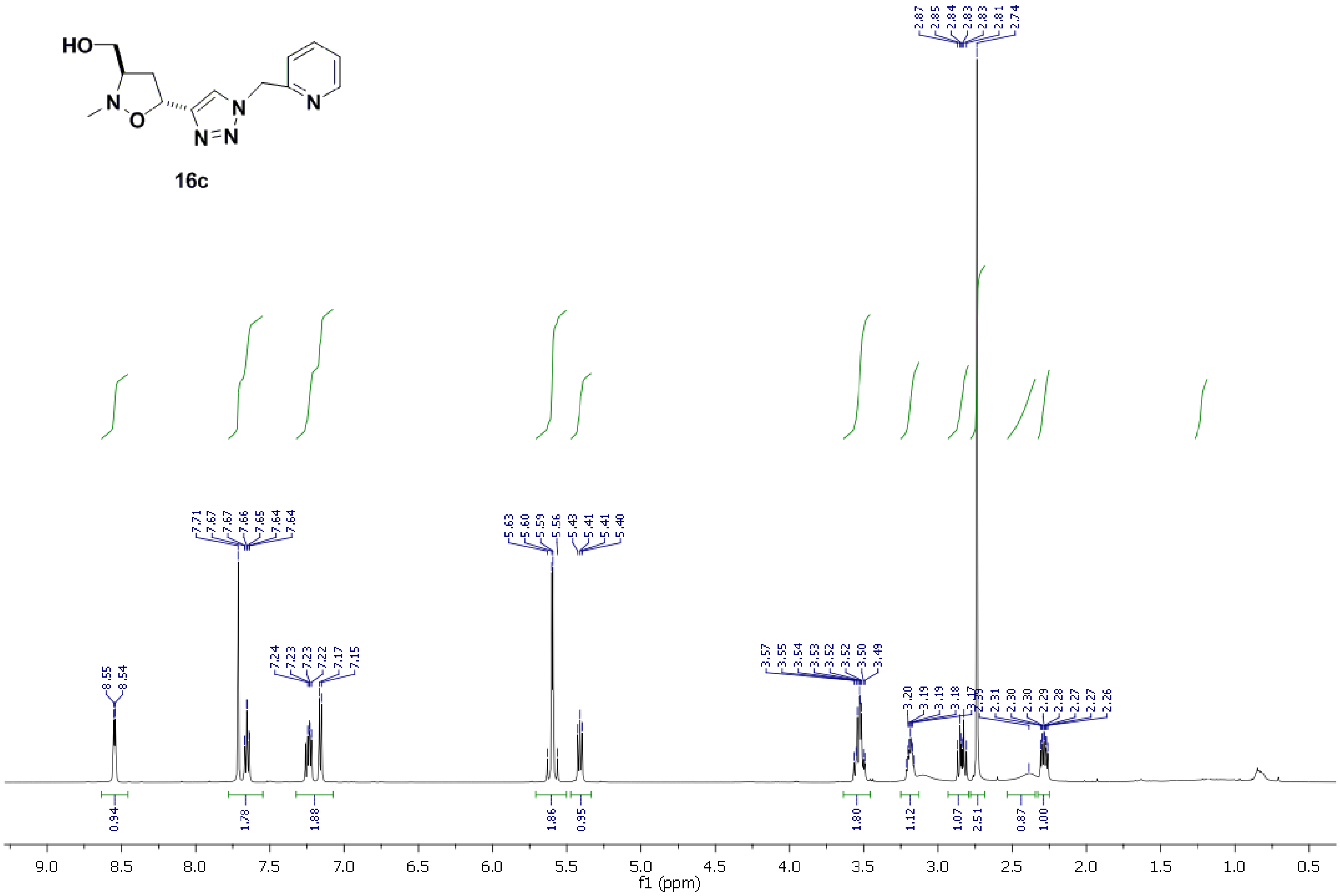

Figure S39. ^{1}H-NMR (500 MHz, $CDCl_3$) of compound 16c.

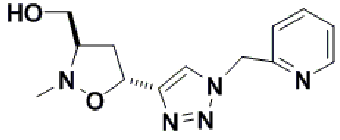

16c

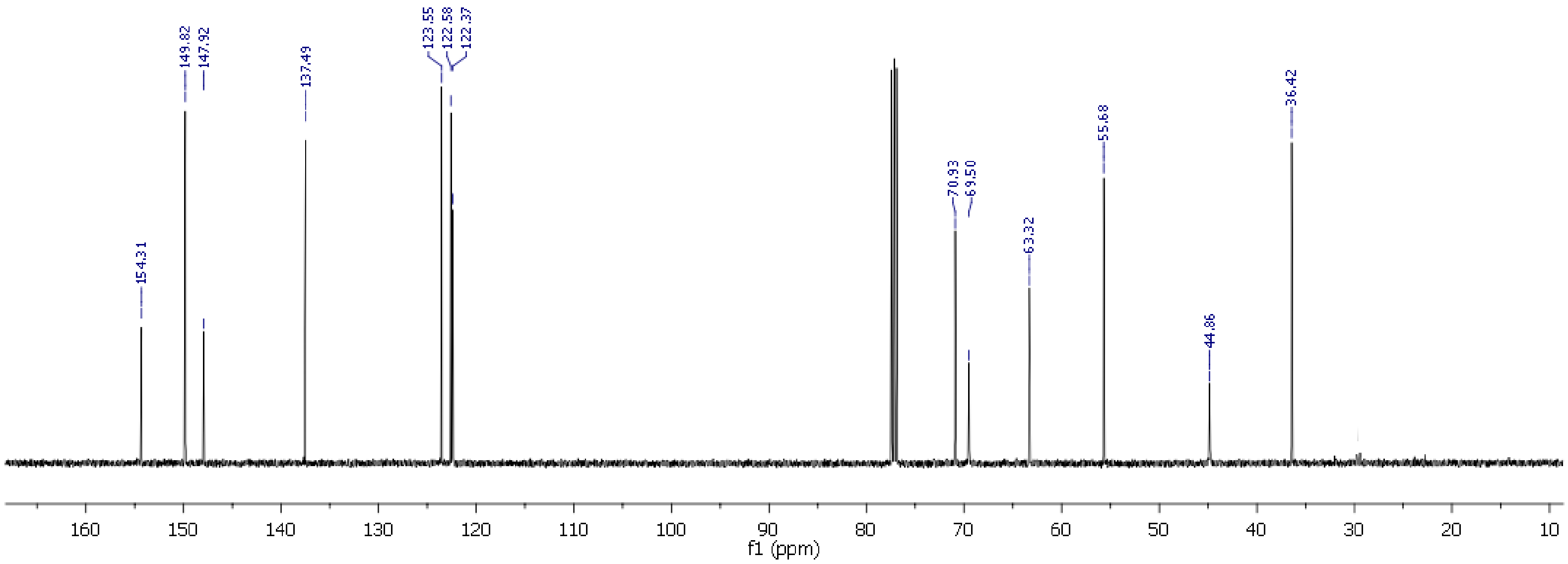

Figure S40. ^{13}C-NMR (125 MHz, $CDCl_3$) of compound **16c**.

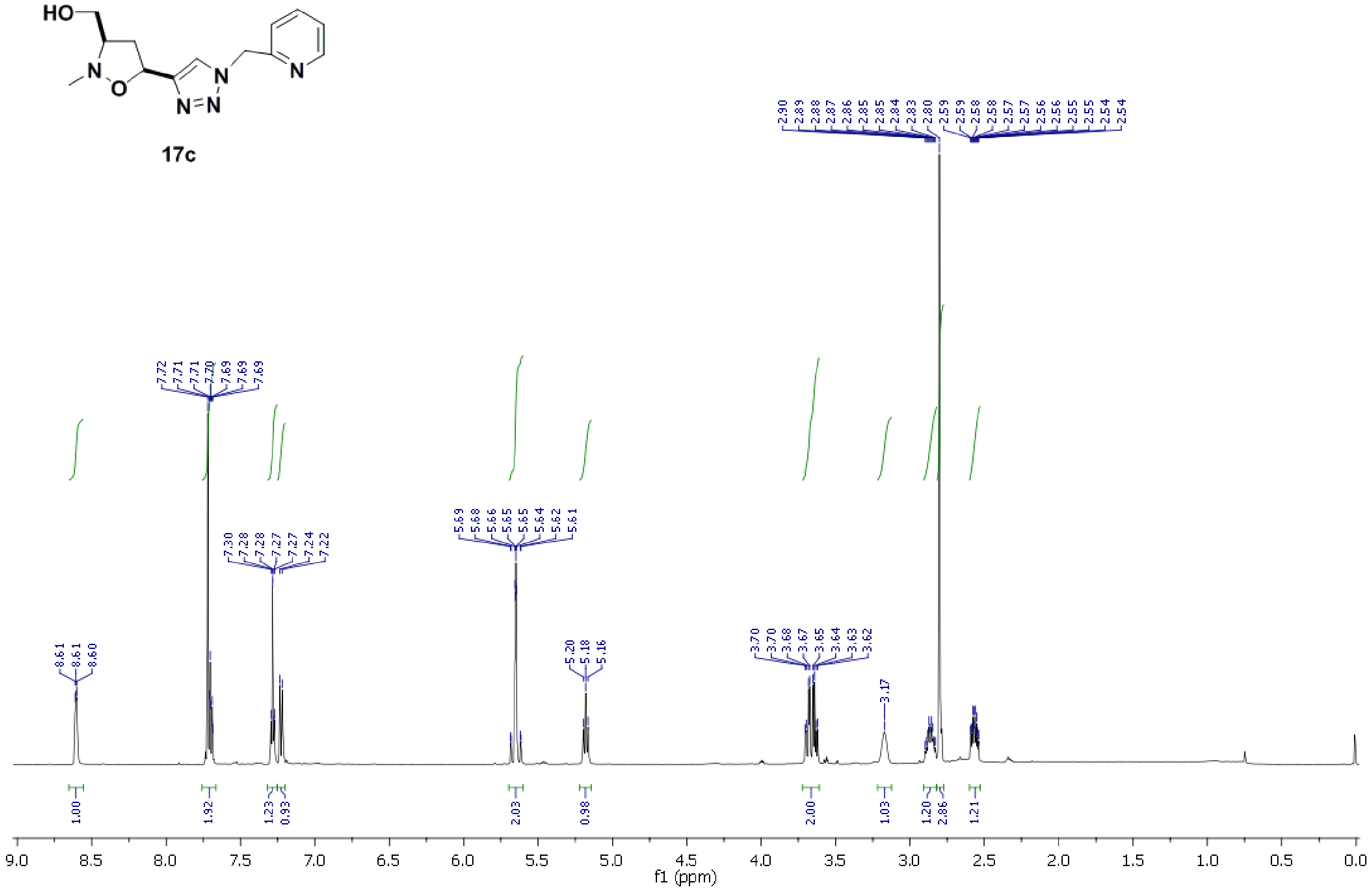

Figure S41. ^{1}H-NMR (500 MHz, $CDCl_3$) of compound **17c**.

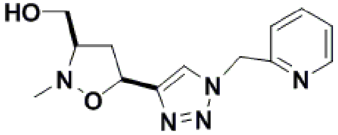

17c

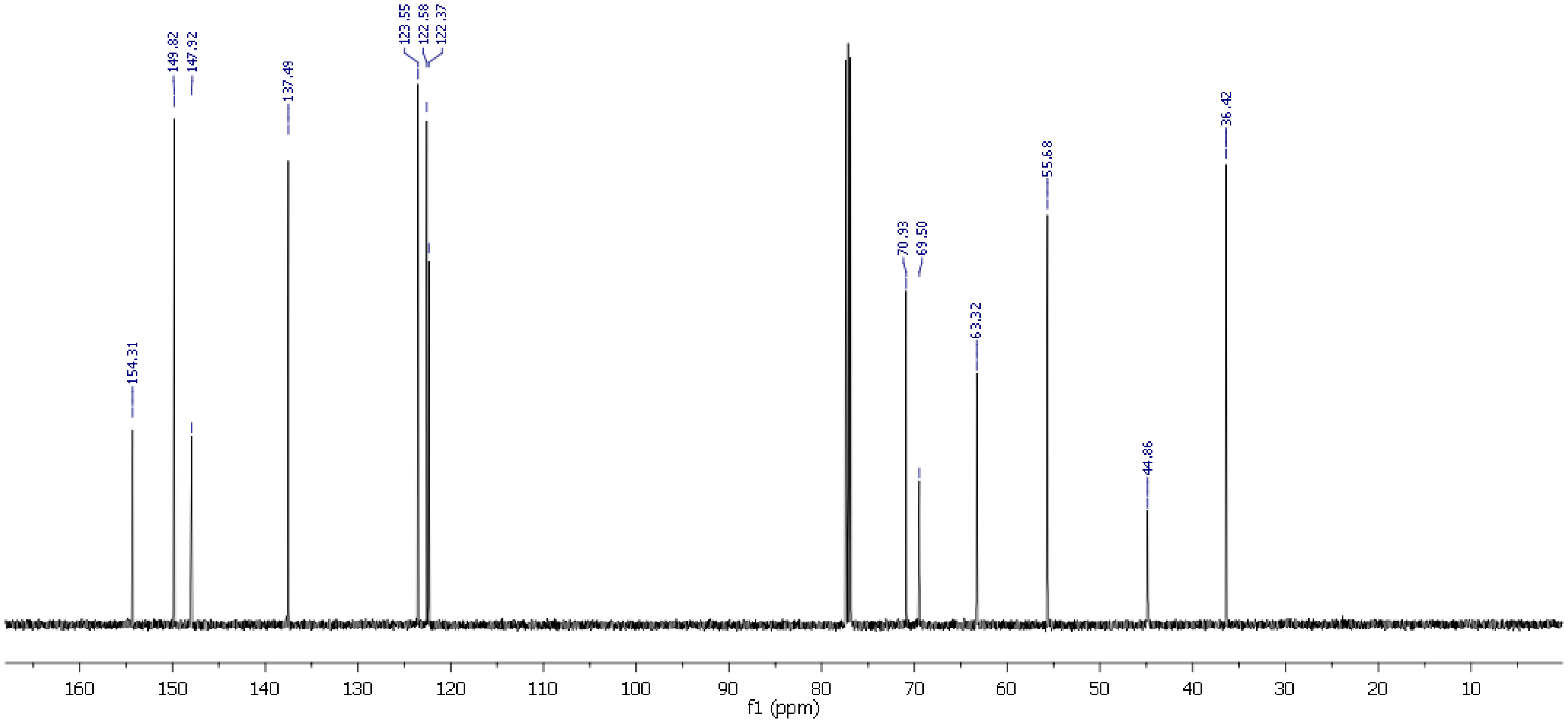

Figure S42. ^{13}C-NMR (125 MHz, $CDCl_3$) of compound **17c**.

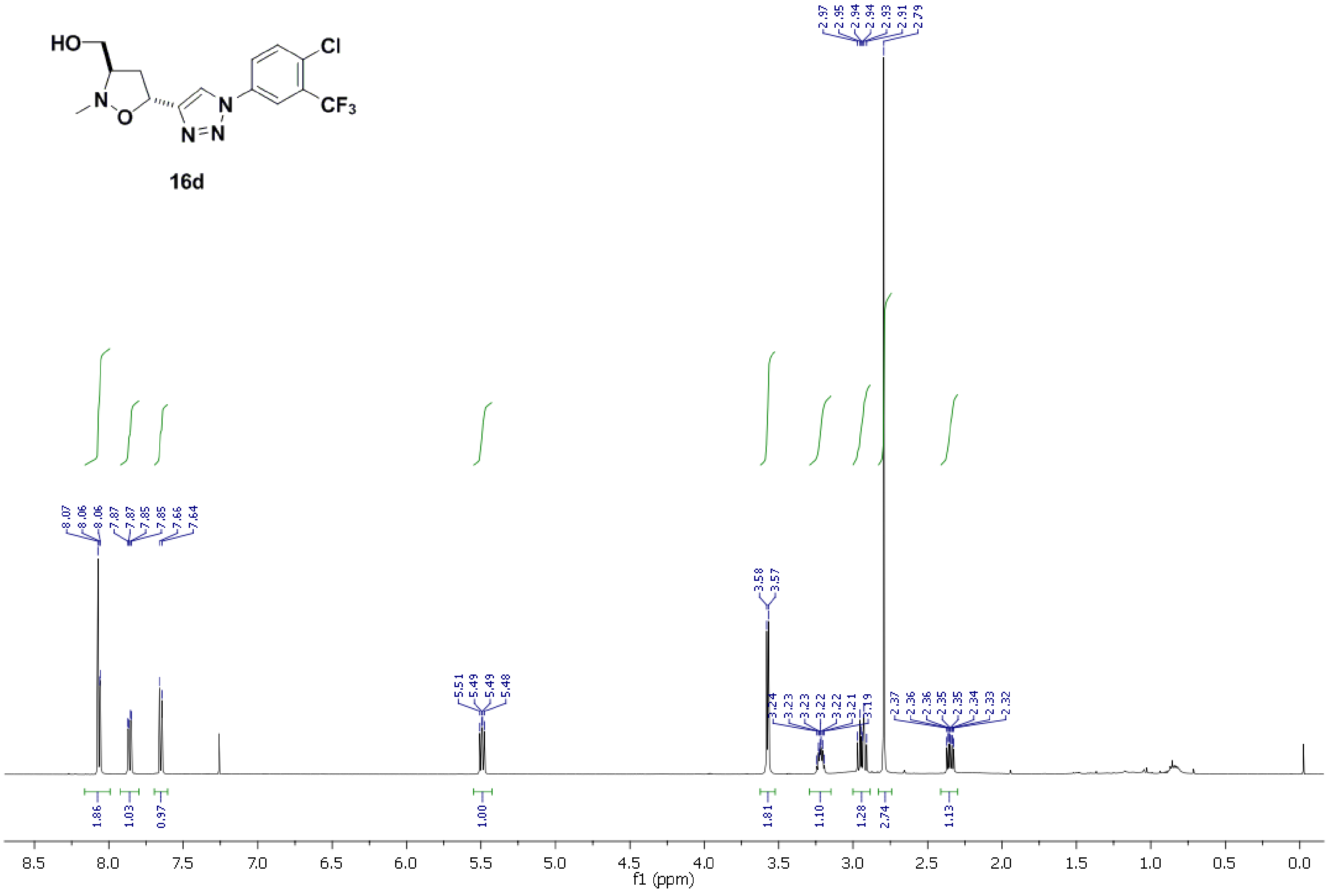

16d

Figure S43. ^{1}H-NMR (500 MHz, $CDCl_3$) of compound **16d**.

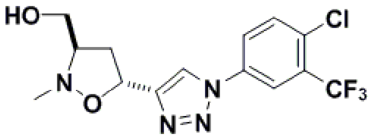

16d

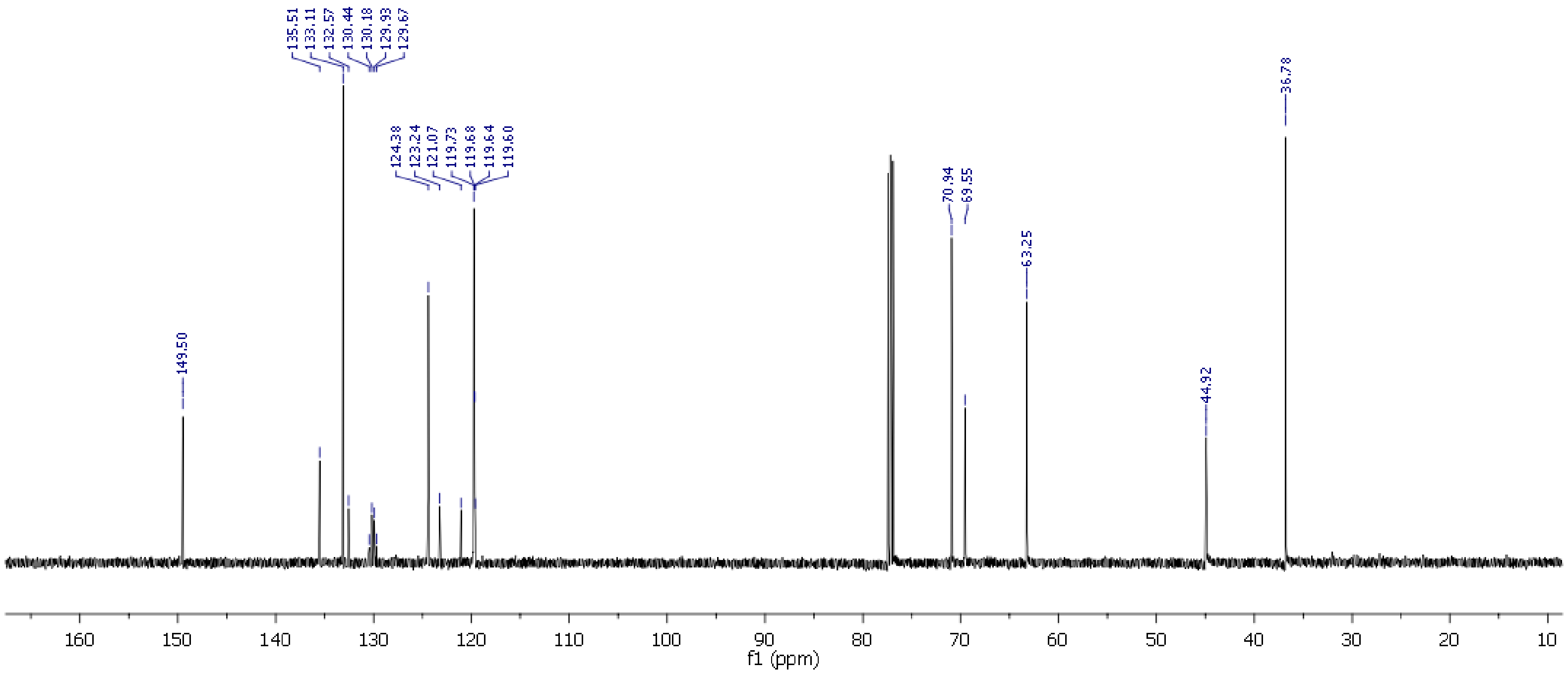

Figure S44. ^{13}C-NMR (125 MHz, $CDCl_3$) of compound **16d**.

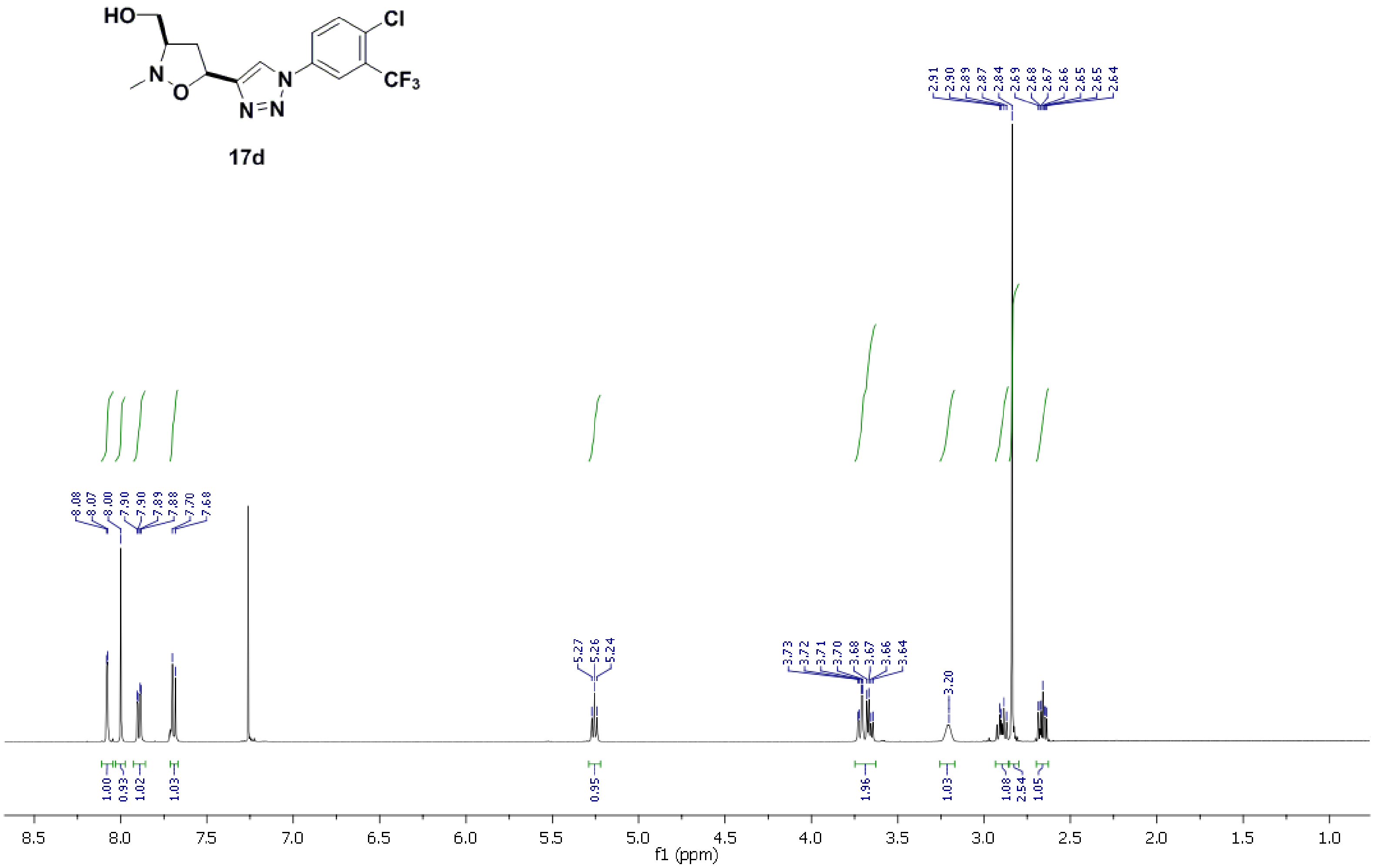

Figure S45. ^{1}H-NMR (500 MHz, $CDCl_3$) of compound **17d**.

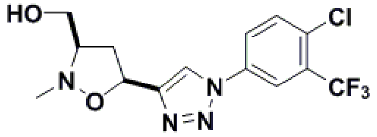

17d

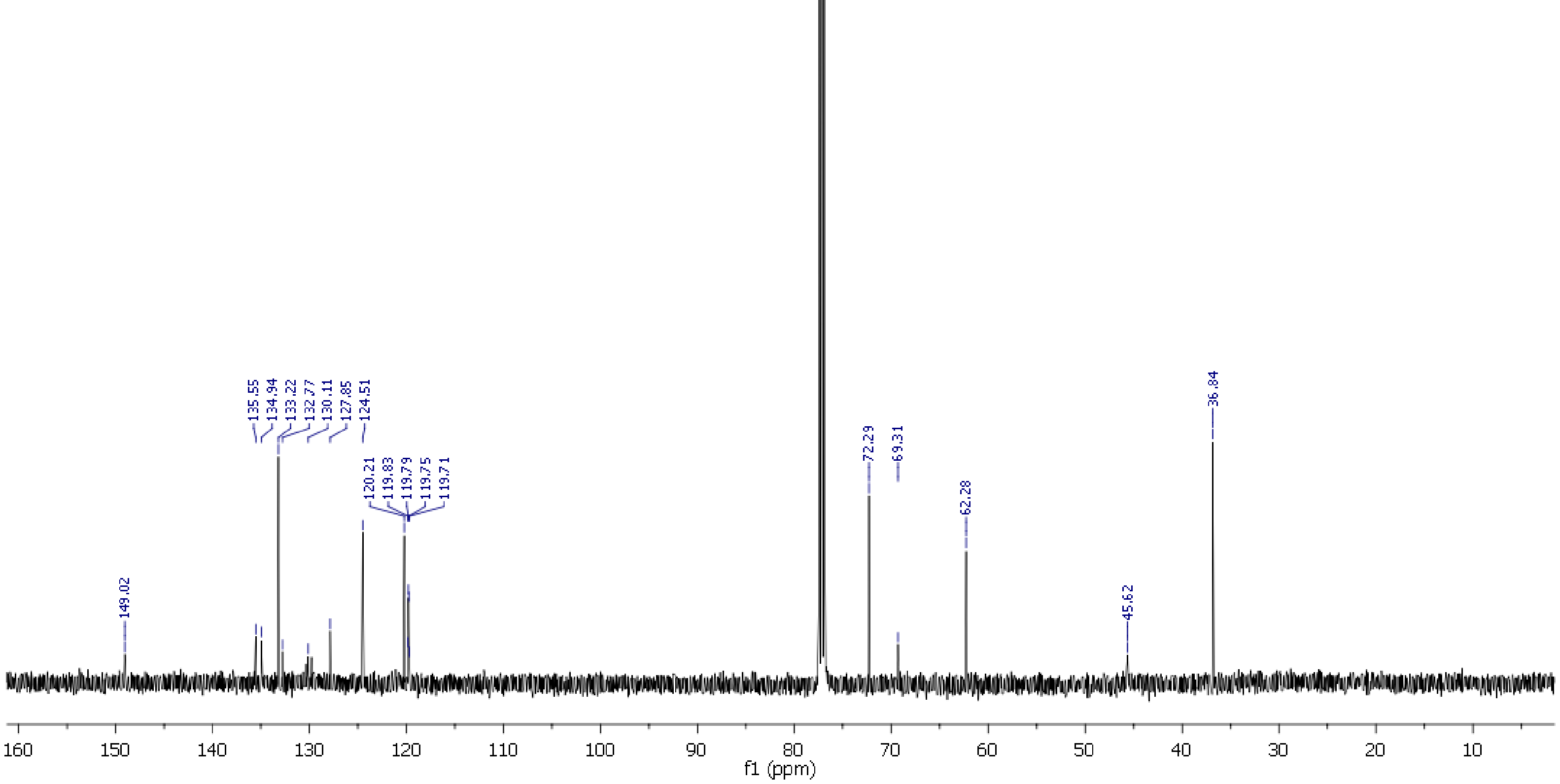

Figure S46. ^{13}C-NMR (125 MHz, $CDCl_3$) of compound **17d**.

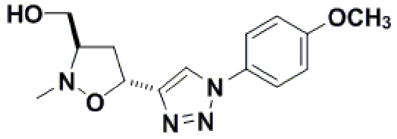

16e

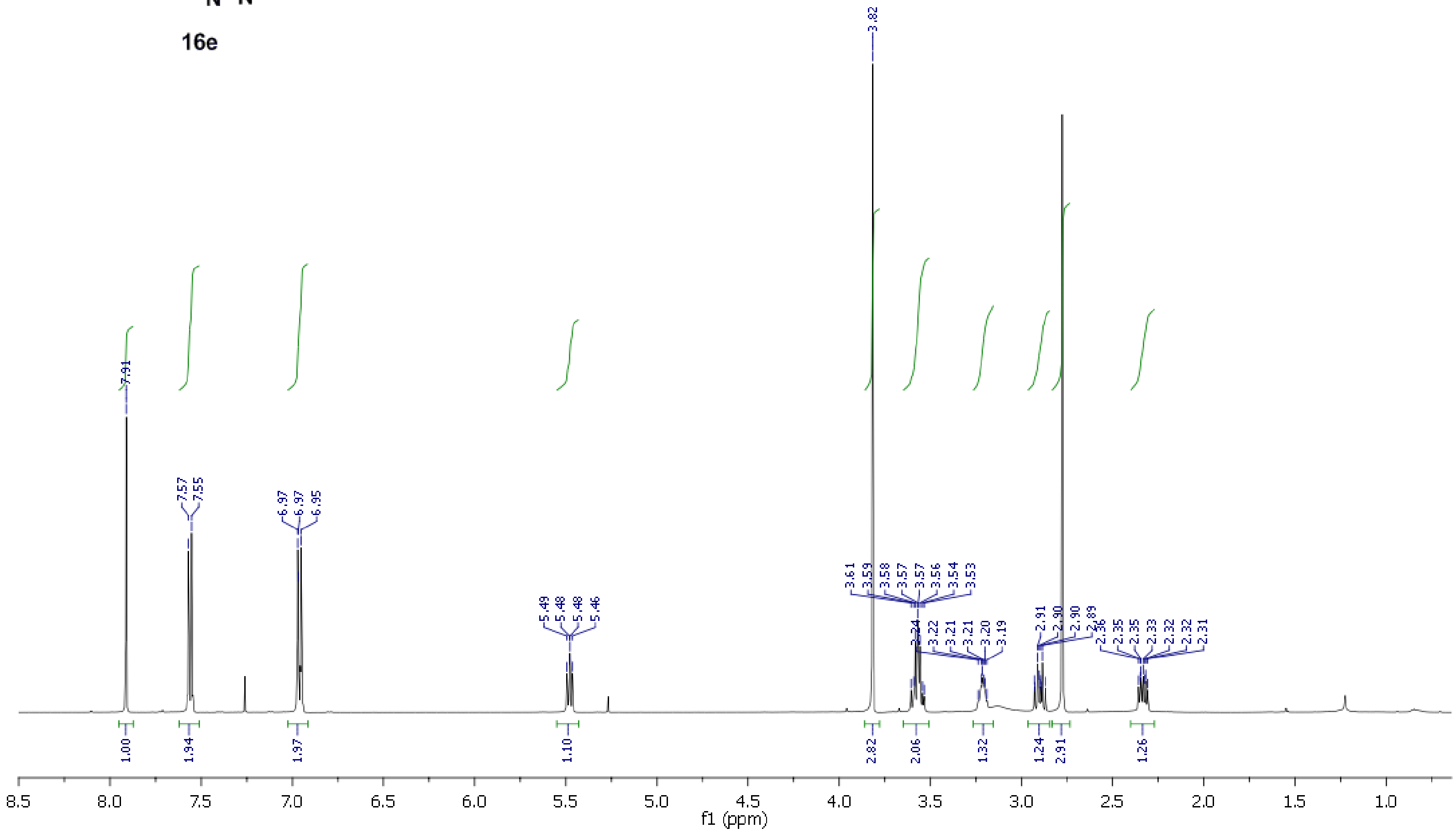

Figure S47. ^{1}H-NMR (500 MHz, $CDCl_3$) of compound **16e**.

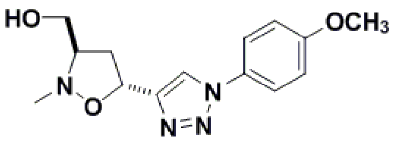

16e

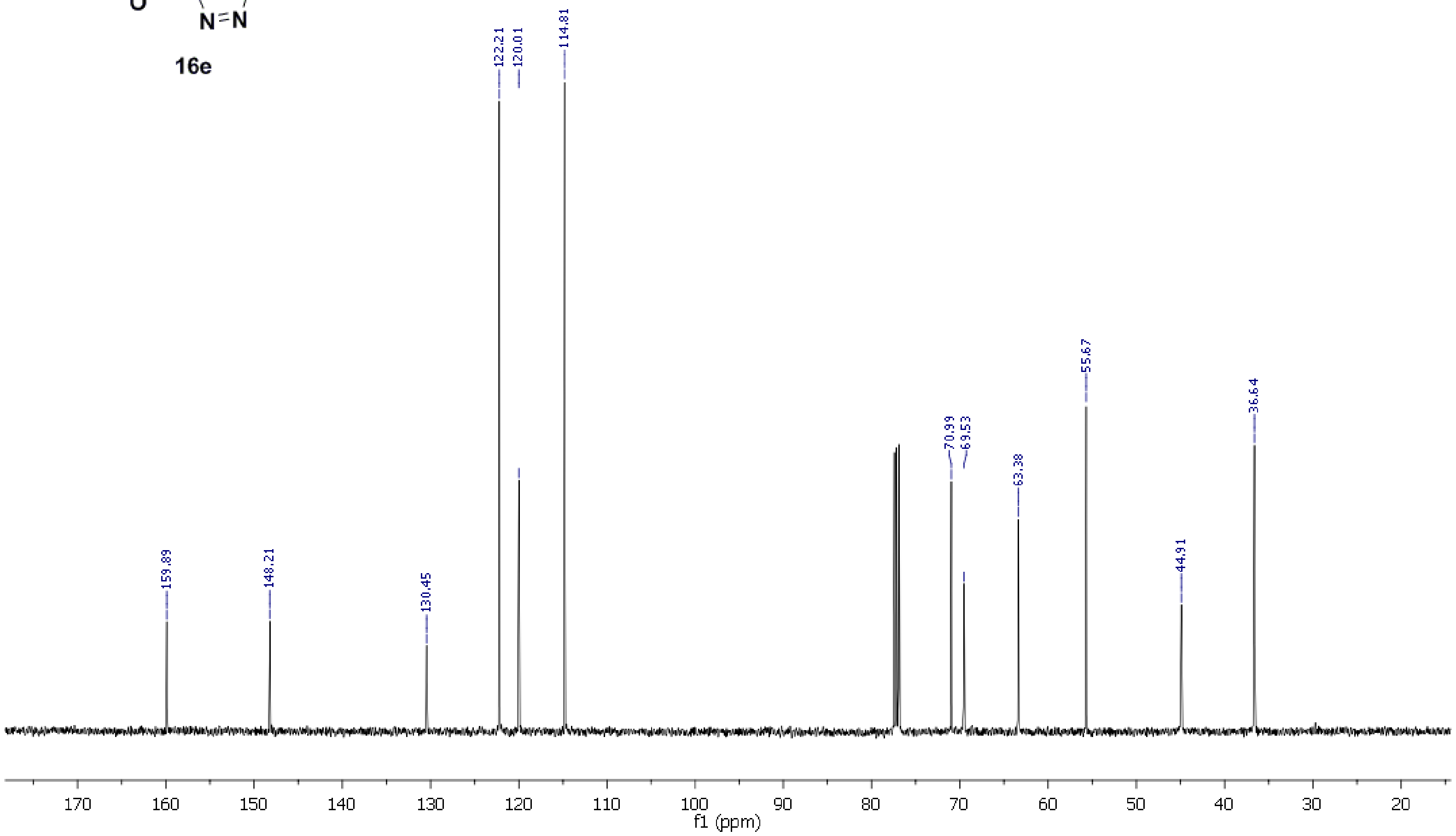

Figure S48. ^{13}C-NMR (125 MHz, $CDCl_3$) of compound **16e**.

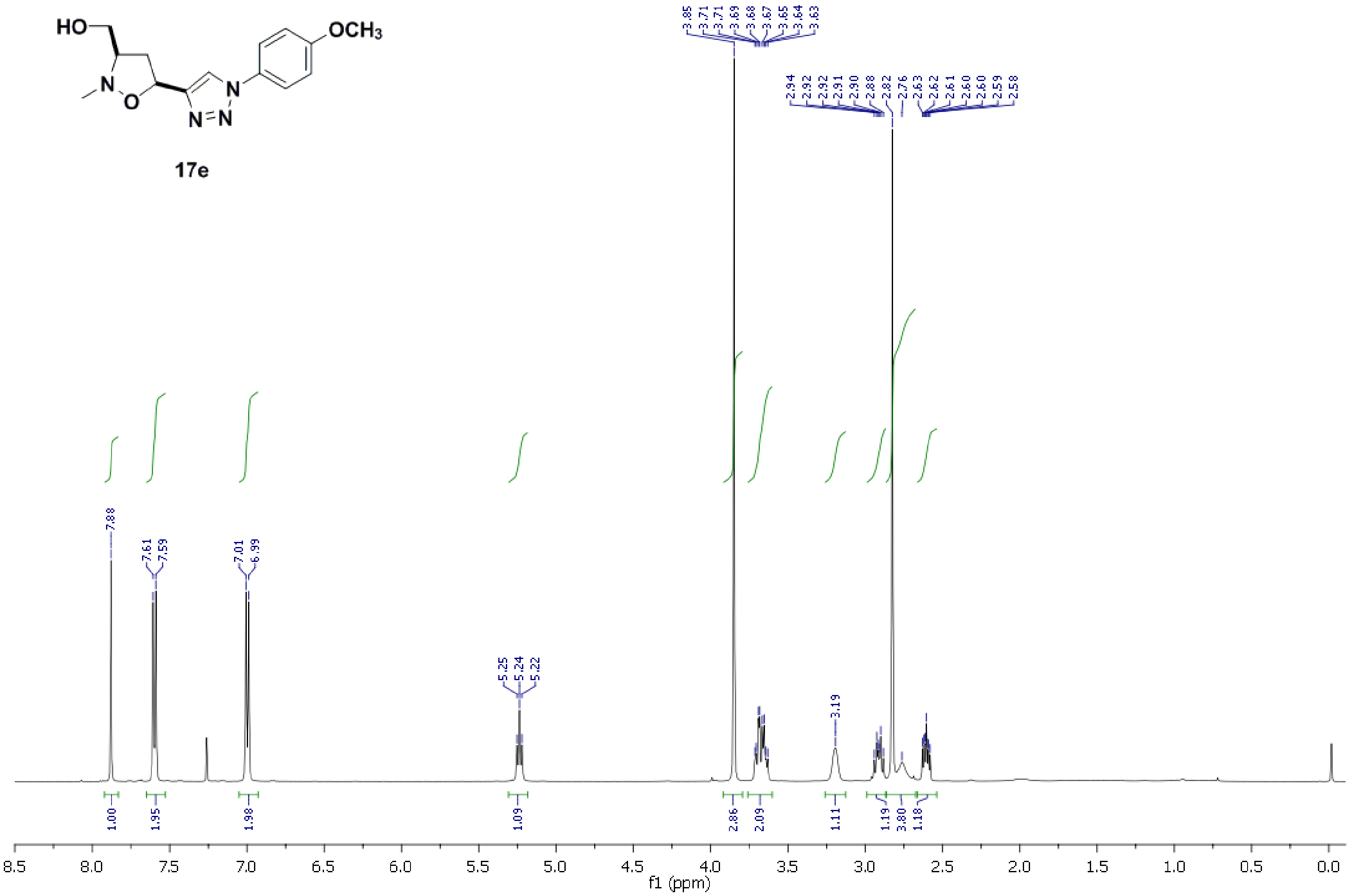

Figure S49. ^{1}H-NMR (500 MHz, $CDCl_3$) of compound **17e**.

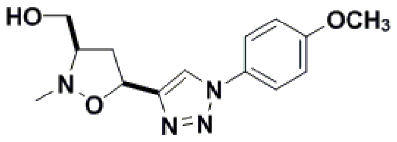

17e

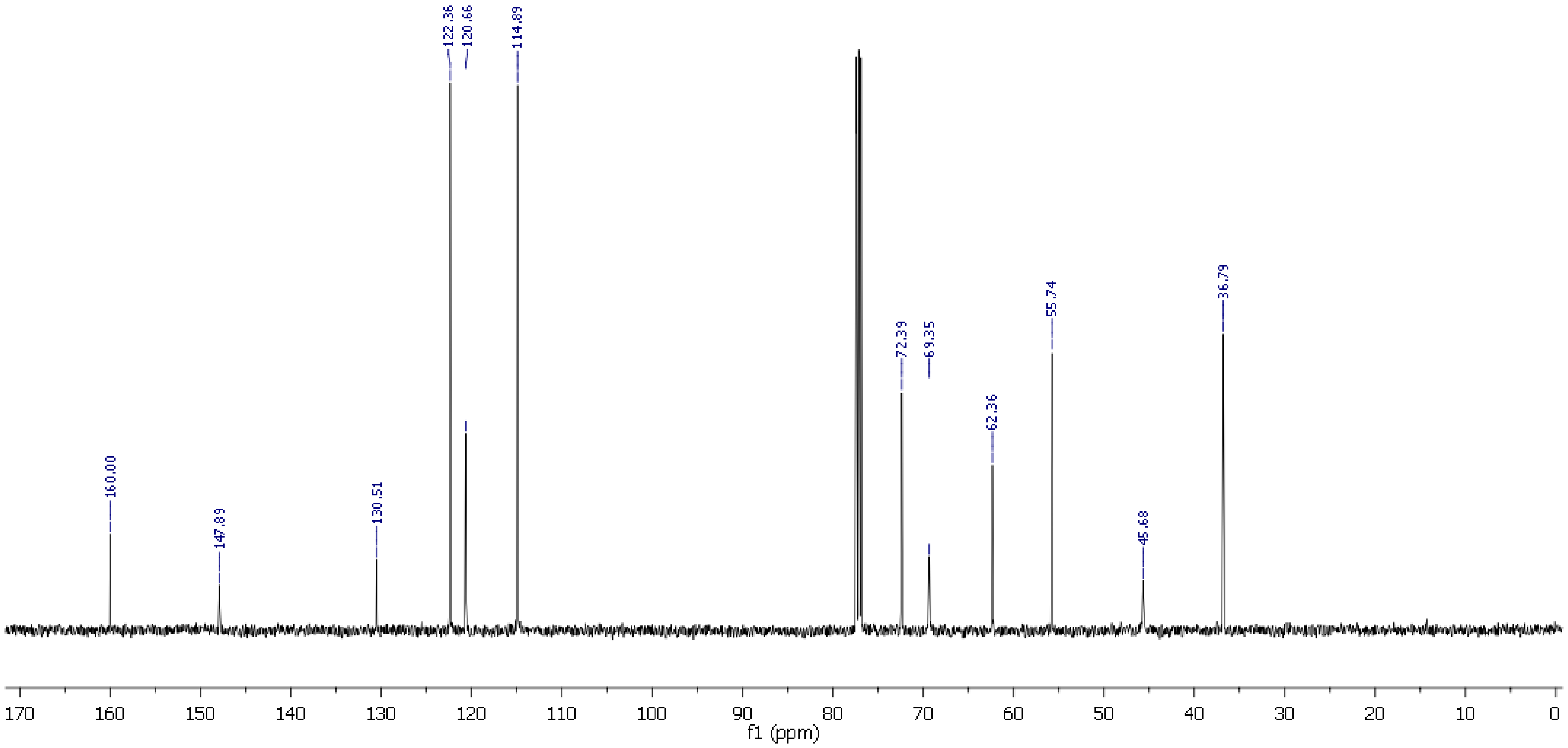

Figure S50. ^{13}C-NMR (125 MHz, $CDCl_3$) of compound **17e**.

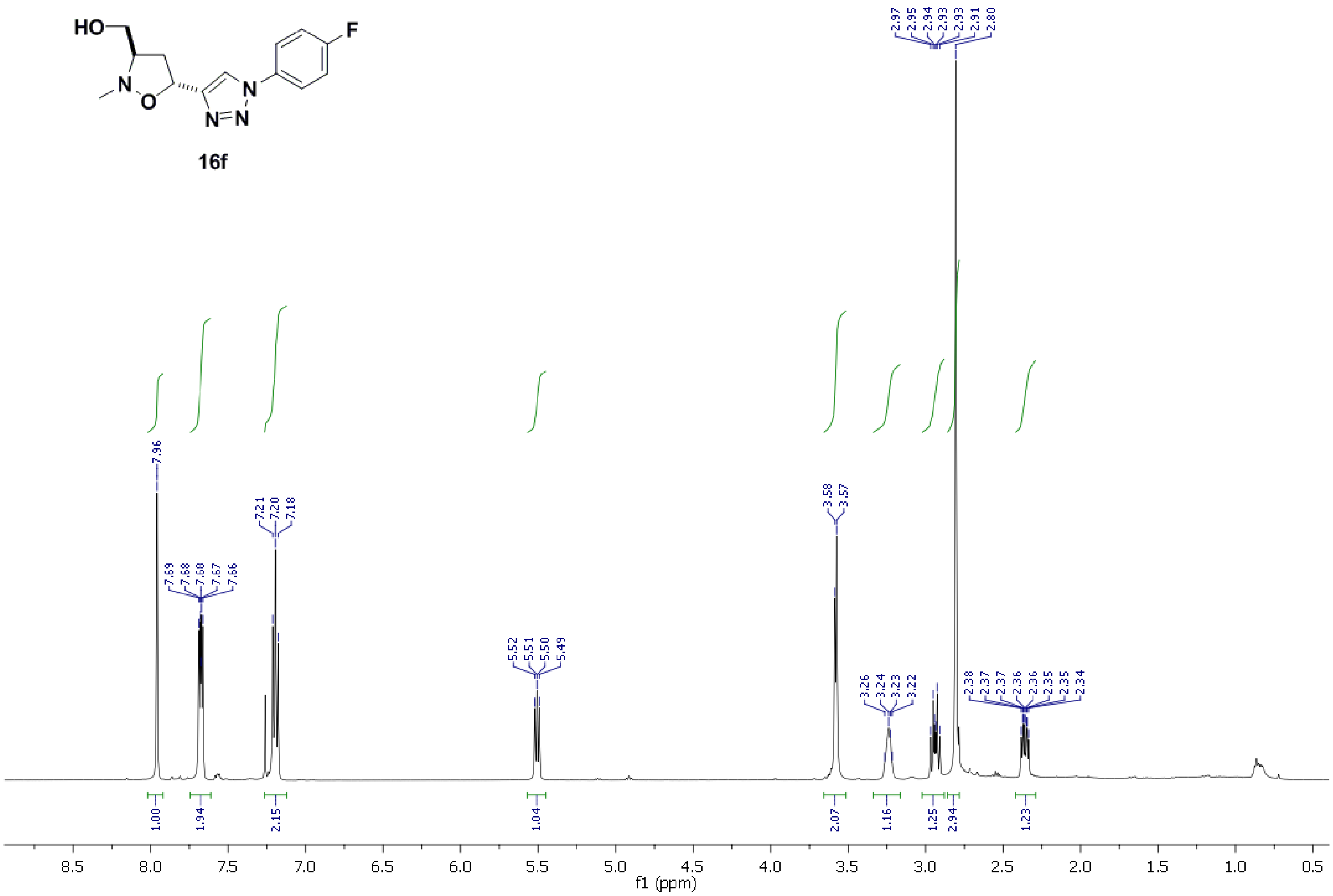

Figure S51. ^{1}H-NMR (500 MHz, $CDCl_3$) of compound **16f**.

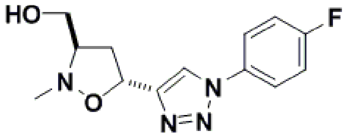

16f

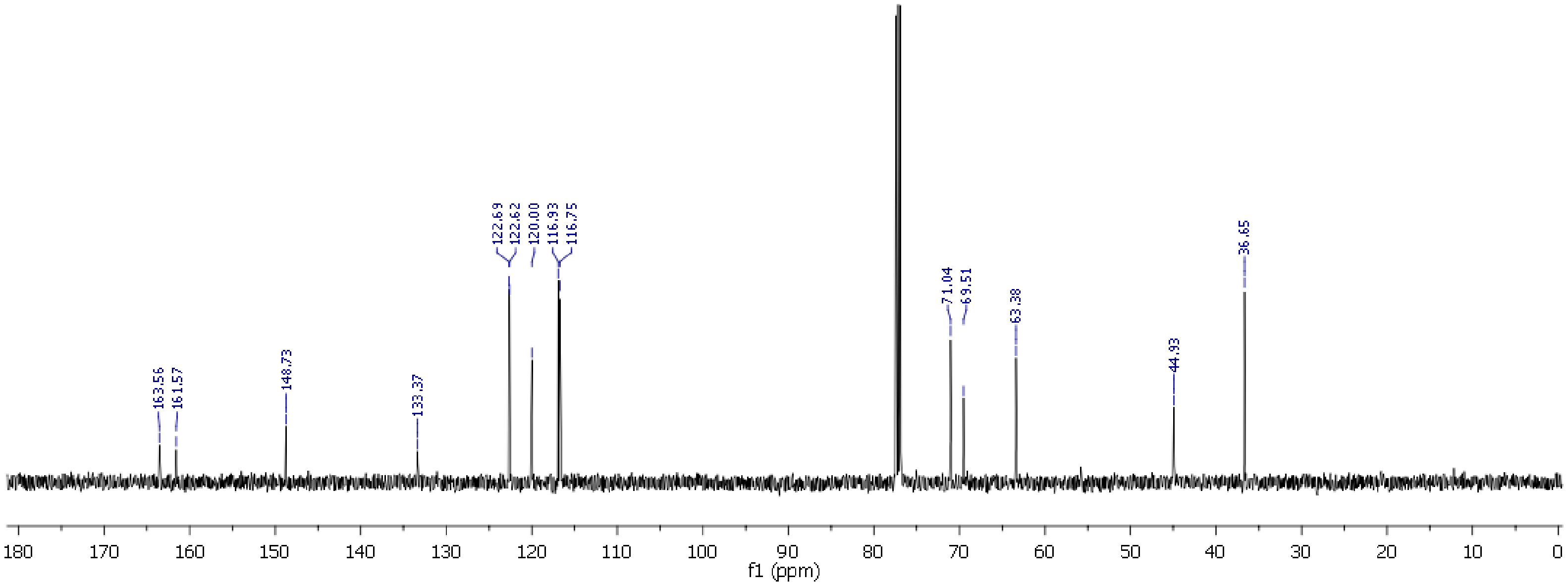

Figure S52. ^{13}C-NMR (125 MHz, $CDCl_3$) of compound **16f**.

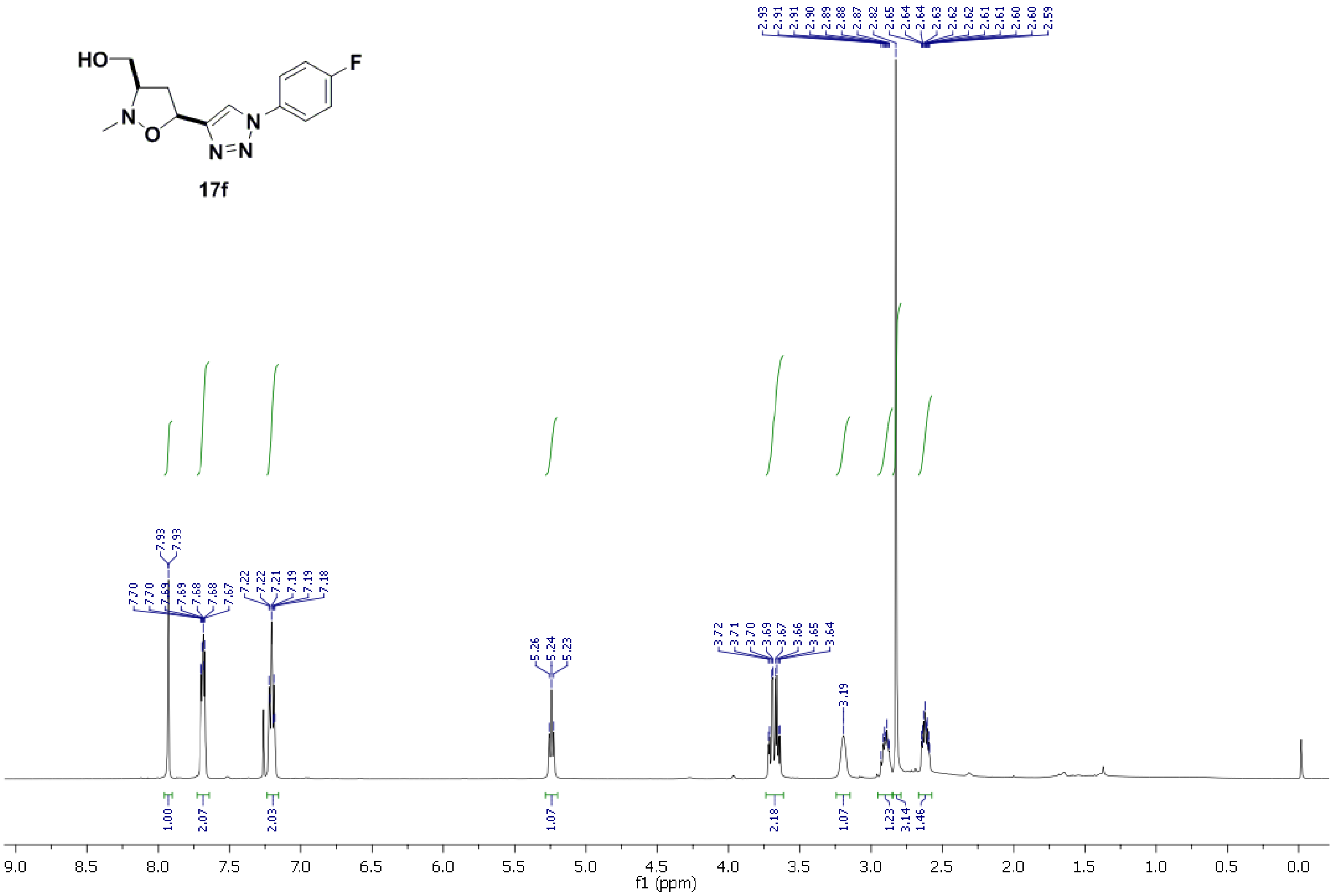

17f

Figure S53. ^{1}H-NMR (500 MHz, $CDCl_3$) of compound **17f**.

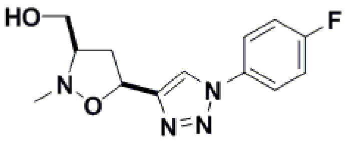

17f

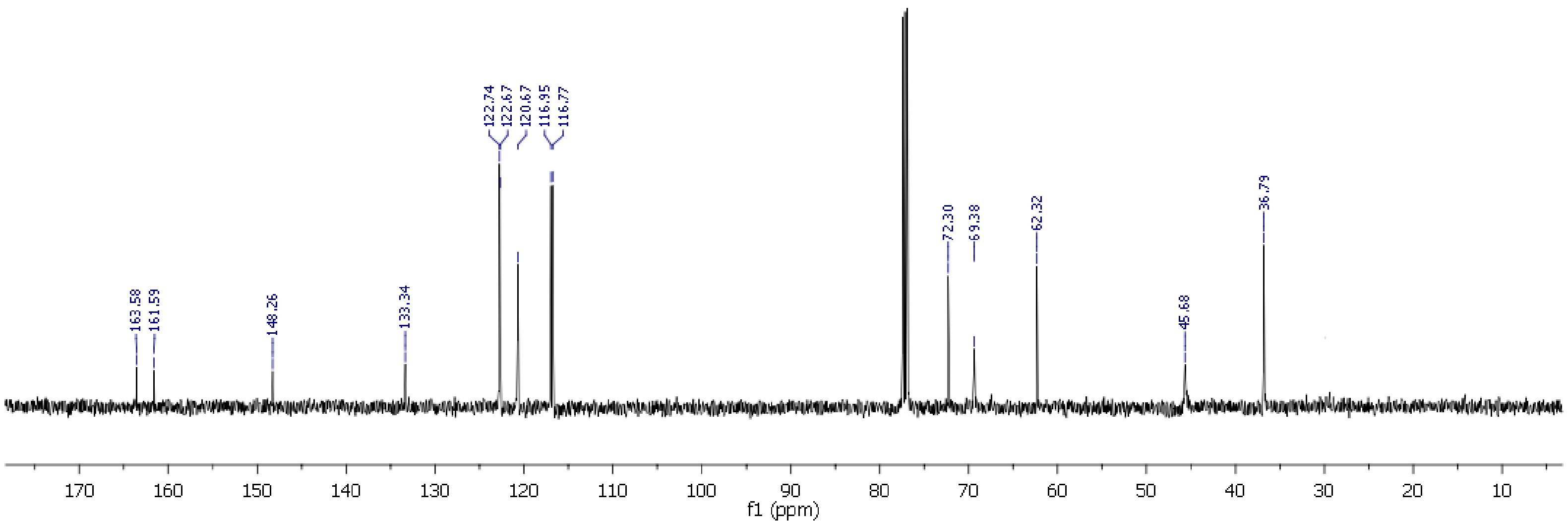

Figure S54. ^{13}C-NMR (125 MHz, $CDCl_3$) of compound **17f**.

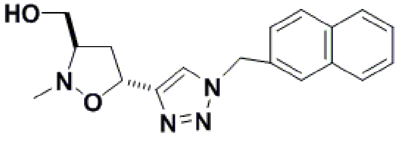

16g

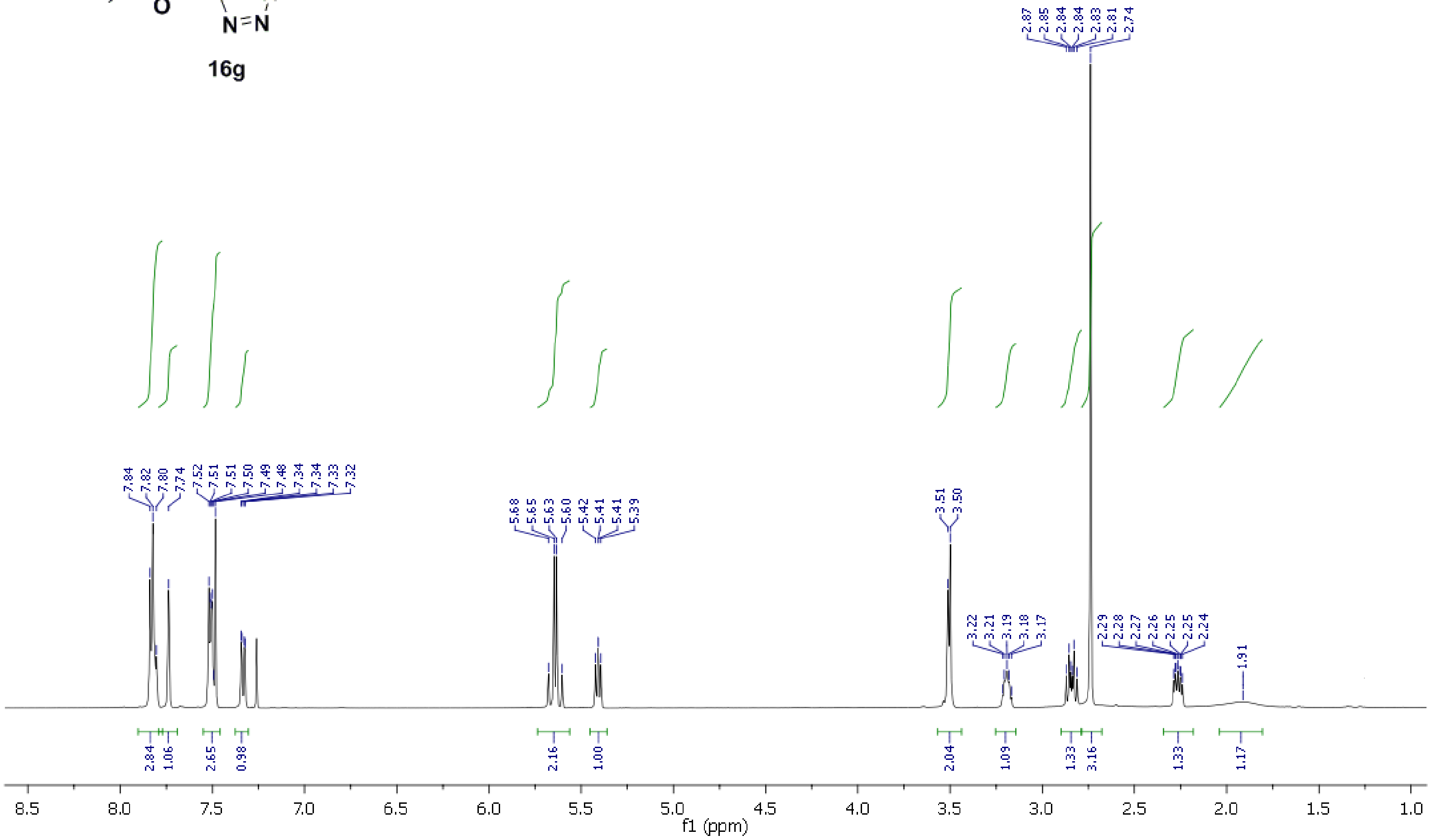

Figure S55. ^{1}H-NMR (500 MHz, CDCl$_3$) of compound **16g**.

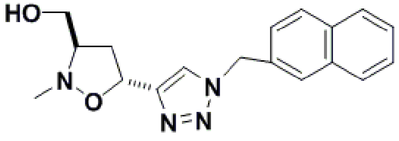

16g

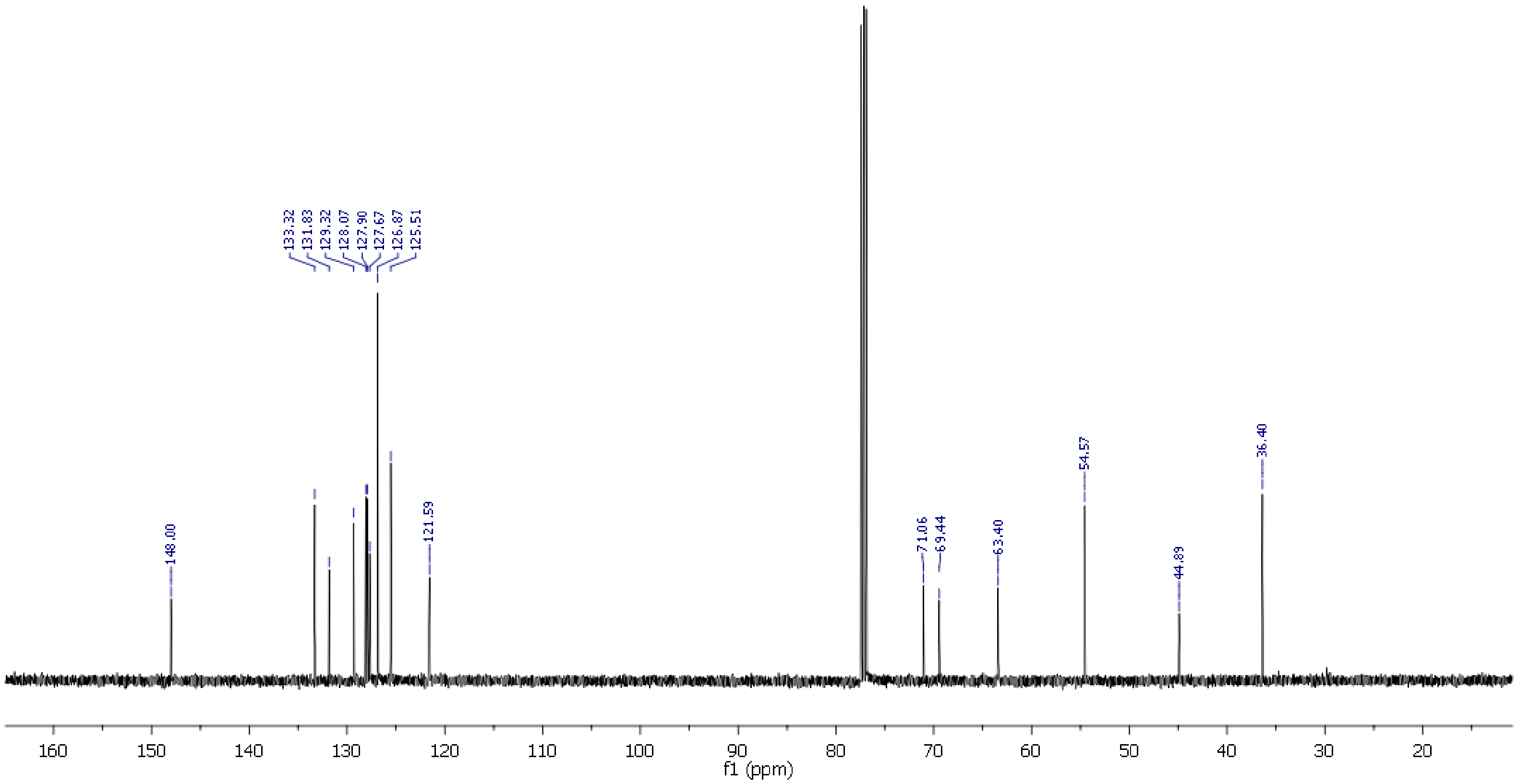

Figure S56. ^{13}C-NMR (125 MHz, $CDCl_3$) of compound **16g**.

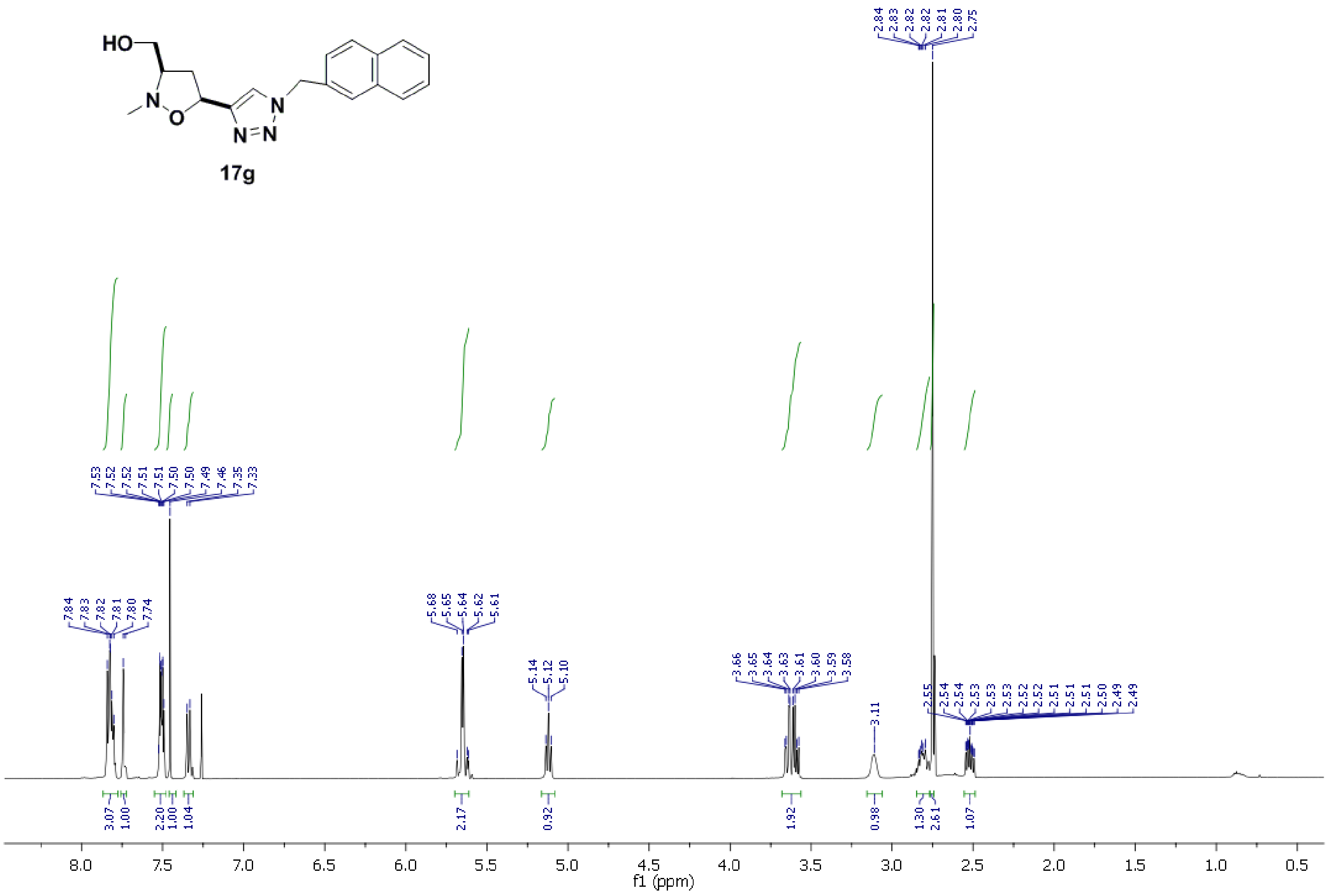

17g

Figure S57. ^{1}H-NMR (500 MHz, $CDCl_3$) of compound **17g**.

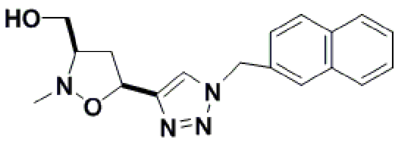

17g

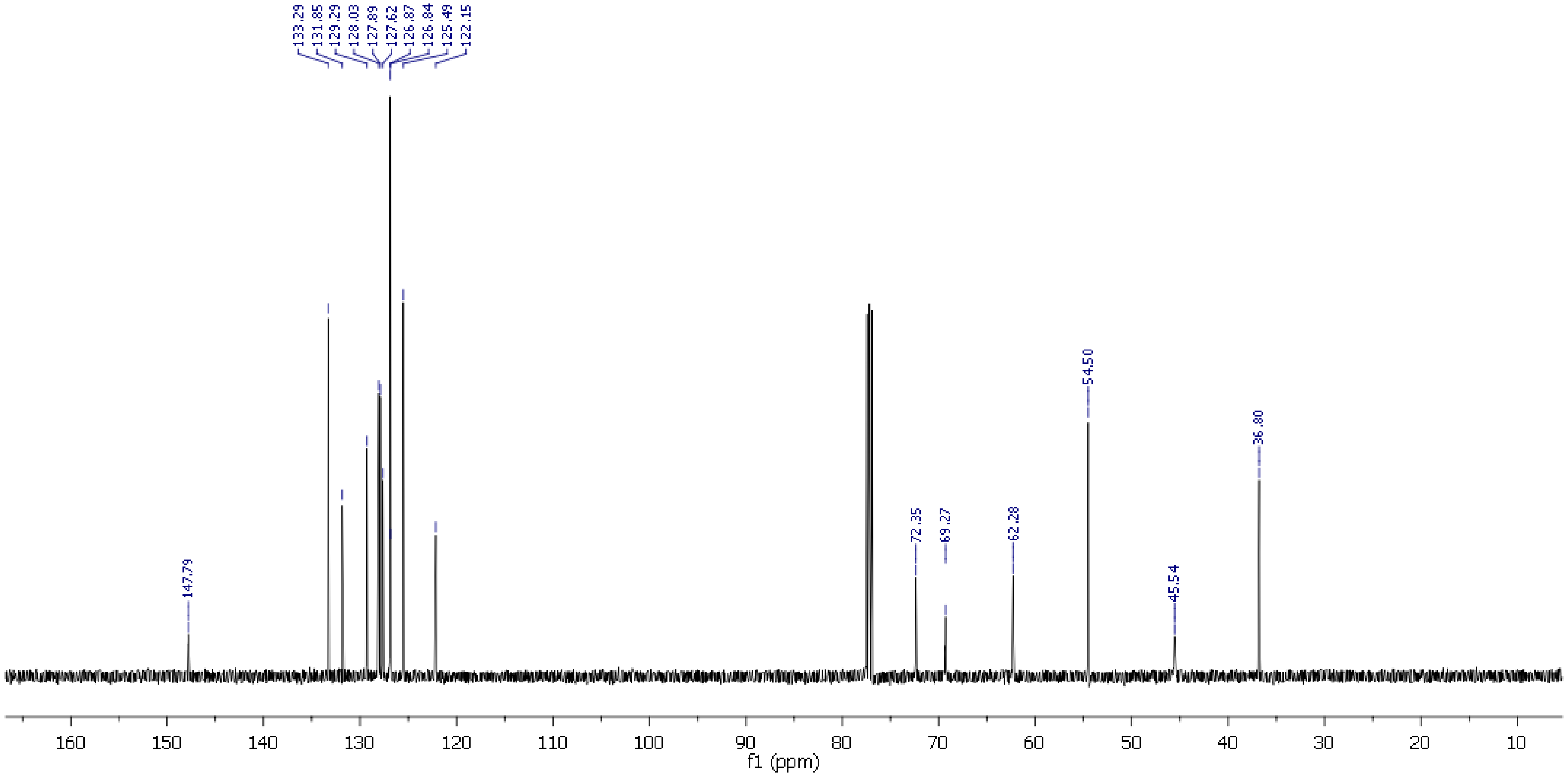

Figure S58. ^{13}C-NMR (125 MHz, $CDCl_3$) of compound **17g**.
